# A Review of the Genus *Ambulyx* Westwood, 1847 (Lepidoptera: Sphingidae) from China Based on Morphological and Phylogenetic Analyses, with the Description of a New Species

**DOI:** 10.3390/insects16020223

**Published:** 2025-02-18

**Authors:** Zhuo-Heng Jiang, Ian J. Kitching, Xiao-Dong Xu, Zhen-Bang Xu, Ming Yan, Wen-Bo Yu, Chang-Qiu Liu, Shao-Ji Hu

**Affiliations:** 1 Yunnan Key Laboratory of International Rivers and Transboundary Eco-Security, Yunnan University, Kunming 650500, China; jzhsphingidae@163.com (Z.-H.J.); zhenbangxumm@gmail.com (Z.-B.X.); 2 Institute of International Rivers and Eco-Security, Yunnan University, Kunming 650500, China; 3 School of Life Science, Westlake University, Hangzhou 310023, China; xuxiaodong@westlake.edu.cn; 4 Natural History Museum, Cromwell Road, London SW7 5BD, UK; i.kitching@nhm.ac.uk; 5 Anhui Provincial Key Laboratory of the Conservation and Exploitation of Biological Resources, College of Life Sciences, Anhui Normal University, Wuhu 241000, China; 15050598630@163.com; 6 College of Landscape Architecture, Nanjing Forestry University, Nanjing 210037, China; yuwenbo@njfu.edu.cn; 7 Guangxi Institute of Botany, Chinses Academy of Sciences, Guilin 541006, China; changqiuliu@whu.edu.cn

**Keywords:** *Ambulyx*, phylogeny, new species, new records, genital structure

## Abstract

The taxonomy of genus *Ambulyx* Westwood, 1847 from China is reviewed based on analysis of wing morphology, male and female genitalia and phylogenetic relationships derived from DNA barcodes. A new species, *Ambulyx wukong* sp. nov. is described from NW Yunnan, China. A male of the rare species, *A. zhejiangensis* from Yintiaoling Nature Reserve, Chongqing, China is examined and its male genitalia illustrated for the first time. Two taxa are newly recorded from China, *A. tattina tattina* from Xishuangbanna, Yunnan, and *A. semiplacida montana* from Pingbian, Yunnan. Distribution maps, biological notes, and ecological records are also given.

## Simple Summary

The genus *Ambulyx* Westwood, 1847 (Lepidoptera, Sphingidae, Smerinthinae, Macroglossini) currently comprises 57 species, 18 of which are found in China, among which complex and confusing taxonomic issues have long existed. We performed an analysis of the *Ambulyx* species of China based on a 658 bp region of the COI mitochondrial gene (DNA barcode) and morphological characters such as wing patterns and genital structure. We describe a new species, *Ambulyx wukong* Jiang & Kitching **sp. nov.**, belonging to the *placida*-group and newly record two taxa, *Ambulyx tattina tattina* and *A. semiplacida montana*, all from the province of Yunnan.

## 1. Introduction

The genus *Ambulyx* Westwood, 1847 (Lepidoptera, Sphingidae, Smerinthinae, Ambulycini) was established as a subgenus of *Sphinx* Linnaeus, 1758, with the type species *Sphinx substrigilis* Westwood, 1847 by monotypy [1]. Species of genus *Ambulyx* are distributed from the Eastern Palaearctic to the Oriental and Australian regions, and currently comprises 57 species, 16 of which have previously been found in China [2]: *A. canescens* (Walker, 1865), *A. japonica* Rothschild, 1894, *A. kuangtungensis* (Mell, 1922), *A. latifascia* Brechlin & Haxaire, 2014, *A. liturata* Butler, 1875, *A. maculifera* Walker, 1866, *A. moorei* Moore, 1858, *A. ochracea* Butler, 1885, *A. placida* Moore, 1888, *A. schauffelbergeri* Bremer & Grey, 1853, *A. semiplacida* Inoue, 1990, *A. sericeipennis* Butler, 1875, *A. tobii* Inoue, 1976, *A. siamensis* Inoue, 1991, *A. substrigilis*, and *A. zhejiangensis* Brechlin, 2009 [1,3,4,5,6,7,8,9,10,11,12,13,14,15,16].

**Figure 1:**
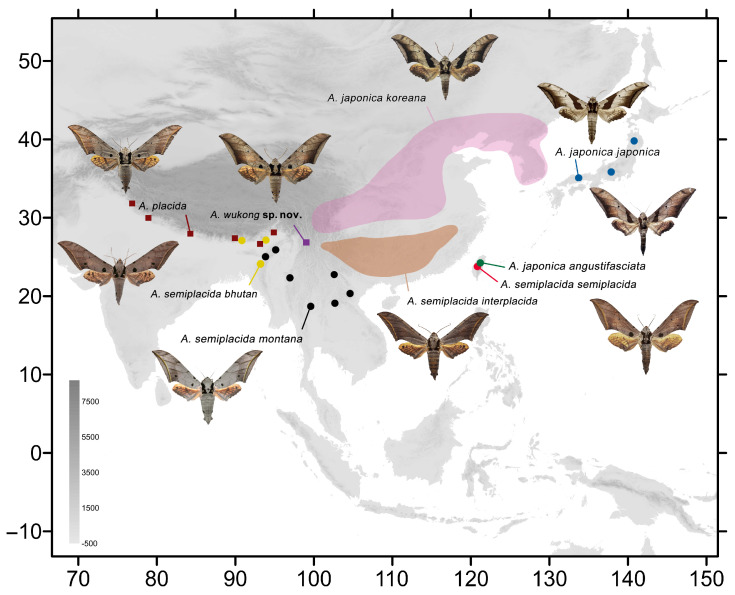
Distribution of species of the *Ambulyx placida*-group: *A. japonica japonica* (blue dots); *A. japonica koreana* (pink patch); *A. japonica angustifasciata* (green dots); *A*. *placida* (ochre squares); *A. semiplacida semiplacida* (red dots); *A. semiplacida montana* (black dots); *A. semiplacida interplacida* (orange patch); *A. semiplacida bhutana* (yellow dots); and *A*. *wukong* **sp. nov.** (purple squares).

The *placida*-group currently includes three species, namely *A. japonica* Rothschild, 1894, *A. placida* Moore, 1888, and *A. semiplacida* Inoue, 1990. *Ambulyx japonica* is a wide ranging species comprising three subspecies: the nominotypical subspecies, which is endemic to Japan; ssp. *koreana* Inoue, 1993, which is found in Korean Peninsula and continental China; and ssp. *angustifasciata* (Okano, 1959), which is endemic to the island of Taiwan [17,18]. *Ambulyx semiplacida* currently consists of four subspecies, some of which were originally described as species: the nominotypical subspecies endemic to Taiwan; ssp. *interplacida* Brechlin, 2006, found in SW and SE continental China; ssp. *bhutana* Brechlin, 2014, found in SW China, Bhutan and India; and ssp. *montana* Cadiou & Kitching, 1990, found in SW China, Vietnam, Laos, Thailand, Myanmar, and India [13,19,20]. The third species, *A. placida*, is found in SW China, Bhutan, Nepal, and India (Figure 1). Between 2016 and 2024, we collected *Ambulyx* specimens from numerous localities in China to study the taxonomic status of the various species and subspecies using morphological and phylogenetic approaches. In this study, by comparing differences in wing pattern and genitalia, together with DNA barcodes sequences, we discovered a fourth species in the *placida*-group and describe it below as *Ambulyx wukong* sp. nov., based on seven males and a female collected at high elevation at Tacheng, Weixi, Yunnan Province.

The rare species, *Ambulyx zhejiangensis* Brechlin, 2009, was described based on a female from Anji county, Zhejiang, China, together with a poor photograph of a possible male specimen that unfortunately lacked precise collecting data [16]. In 2022, we collected an unusual male *Ambulyx* species in Yintiaoling Nature Reserve, Chongqing, China. From morphological and phylogenetic analysis, we confirmed this to be a male of *A. zhejiangensis*. Thus, we redescribe this species and include an illustration of its male genitalia and details of its ecology for the first time.

**Figure 2:**
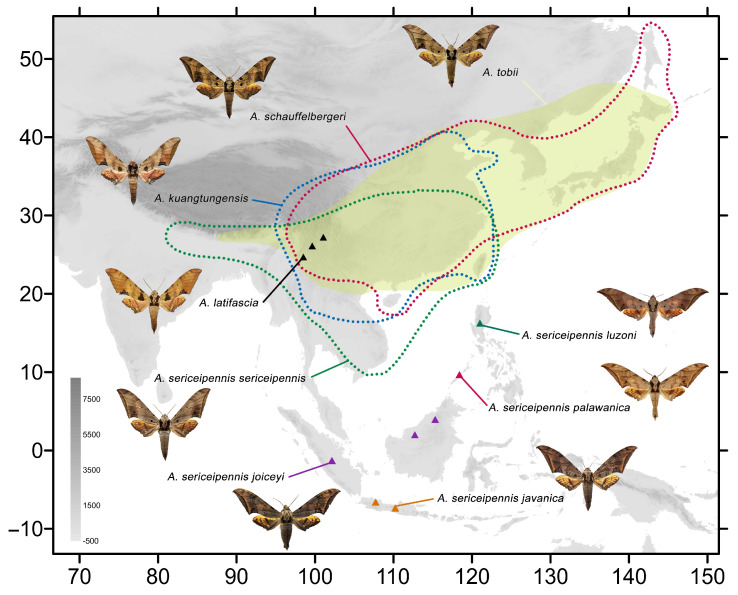
Distribution of species of the *Ambulyx schauffelbergeri*- and *kuangtungensis*-groups included in this study: *A*. *latifascia* (black triangles); *A*. *schauffelbergeri* (red dotted line); *A. sericeipennis sericeipennis* (green dotted line); *A. s*. *joiceyi* (purple triangles); *A. s*. *javanica* (orange triangles); *A*. *s. luzoni* (green triangles); *A. s. palawanica* (red triangles); and *A*. *tobii* (yellow patch).

**Figure 3:**
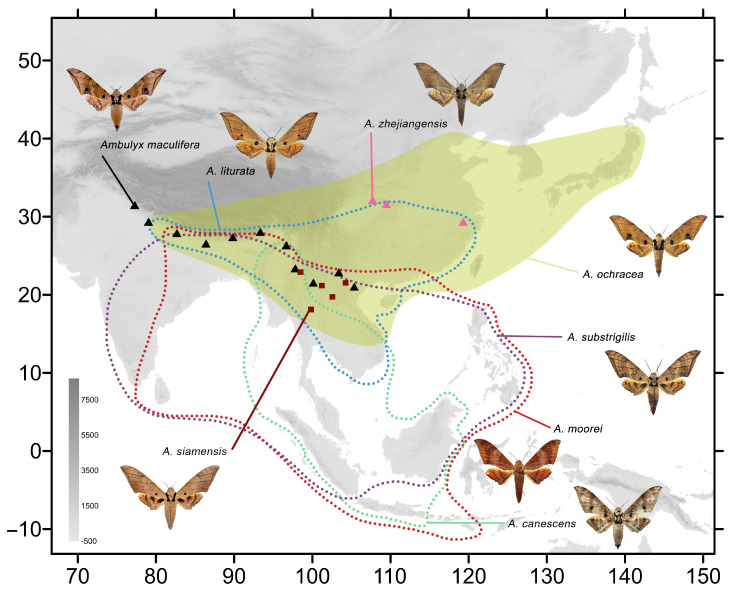
Distribution of the remaining species of *Ambulyx* from China: *A*. *substrigilis* (purple dotted line); *A. siamensis* (ochre squares); *A. liturata* (blue dotted line); *A. moorei* (red dotted line); *A*. *canescens* (green dotted line); *A. ochracea* (yellow patch); *A. maculifera* (black triangles); and *A*. *zhejiangensis* (pink triangles).

The remaining *Ambulyx* species from China are also here illustrated and discussed, with details of their distributional ranges(Figure 2 and Figure 3), and biological and ecological notes. They include two new (sub)species records for China: *A*. *tattina tattina* (Jordan, 1919) from Mengla, Xishuangbanna, Yunnan, China, which had previously been recorded from Laos, Thailand, Vietnam, Malaysia, Indonesia; and *A. semiplacida montana* from Pingbian, Yunnan, China, which was previously recorded from Laos, Vietnam, Thailand, Myanmar, and India. Details of several other *Ambulyx* species are illustrated for the first time: the females of *A*. *semiplacida interplacida* and *A. latifascia* and their genitalia; the male genitalia of *A. zhejiangensis*; the female genitalia of *A. semiplacida semiplacida* and *A. semiplacida montana*; and the larvae of *A. siamensis* and *A. semiplacida montana*. We also divide the species into various species-groups on the basis of a phylogenetic tree derived from maximum likelihood and Bayesian inference analyses of *cox1* (DNA barcode) sequences. We also calculate the evolutionary divergence time-node of the *placida*-group and the *schauffelbergeri*-group based on *cox1* sequences to determine the genetic relationships of the various species in these two interesting *Ambulyx* species-groups.

## 2. Materials and Methods

### 2.1. Taxon Sampling

Most of the *Ambulyx* specimens newly collected for this study were sampled for both morphological and molecular analysis. The majority were dried in paper triangles then stored at −20 °C until used, but some were directly spread after collection to avoid abrasion of the scales on the head, thorax, and abdomen.

For each individual used for molecular analysis, two legs from the same side were taken for DNA extraction before the specimens were rehydrated for spreading. Additional sequences were downloaded from the Barcode of Life Database v.4 (BOLD) (http://www.boldsystems.org (accessed on 13 December 2024)) to provide as complete as possible species coverage for phylogenetic analysis. Collecting data, BOLD sample IDs, and GenBank accession numbers are provided in Table S1, list of the examined *Ambulyx* specimens in this study are shown in Appendix A.

### 2.2. DNA Extraction and Amplification

The phenol–chloroform protocol was used to extract genomic DNA. The legs were-homogenized in protease buffer containing 450 µL STE (10 mmol/L Tris-HCl, 1 mmol/L EDTA, 100 mmol/L NaCl, pH = 8.0), 25 µL Proteinase K (20 mg/mL), and 75 µL SDS (10%) and incubated at 55 °C for 12 h to rehydrate and lyse the tissue. The subsequent extraction was carried out accordingly, and the resultant genomic DNA was preserved at −40 °C.

DNA amplification followed Xu et al. [19]. The polymerase chain reaction (PCR) was carried out in a 25 µL system using the TaKaRa Ex *Taq* Kit (TaKaRa Biotechnology Co., Ltd., Dalian, China). The system contained 2.5 µL 10× PCR buffer, 2.0 µL MgCl_2_ (2.5 mmol/L), and 2.0 µL dNTP mixture (2.5 mmol/L each). The mitochondrial *cox1* gene fragment (the DNA barcode) was amplified and sequenced with the primers LCO1490 (5′-GGT CAA ATC ATA AAG ATA TTG-3′) and HCO2198 (5′-TAA ACT TCA GGG TGA CCAAAA AAT CA-3′) [21]. The PCR thermal profile consisted of an initial denaturation at 95 °C for 3 min, 30 cycles of denaturation at 94 °C for 1 min, annealing at 50 °C for 1 min, and elongation at 72 °C for 1 min; then a final elongation at 72 °C for 5 min. Sequencing was undertaken using an ABI Prism 3730 sequencer (Applied Biosystems, Foster City, CA, USA).

### 2.3. Phylogenetic Analysis and Species Delimitation

We downloaded *cox1* sequences of additional species and subspecies of *Ambulyx* from NCBI, BOLD, and our own sequencing data. These sequences were aligned using MAFFT v 7.525 [21], followed by trimming with trimAL v 1.5.0 to refine the alignment [22]. Phylogenetic trees were constructed using IQ-TREE v 2.3.6 [23] with the options-m MFP-bb 1000-alrt 1000” to select the best-fit model and assess branch support through 1000 ultrafast bootstrap replicates and SH-aLRT tests.

To estimate the divergence times within the *placida*- and *schauffelbergeri*-groups, we employed MCMCTREE of PAML v 4.10.7 [24] with calibration points based on the time-calibrated molecular phylogeny of hawkmoths [25]. All MCMC runs were evaluated using Tracer v1.7.2 [26], ensuring effective sample size (ESS) values exceeded 200, indicating robust parameter estimation. Tree visualization and refinement were performed using Figtree v1.4.3, ITOL web server, and ggtree v3.20, with final graphical adjustments made in Adobe Illustrator 2022.

Additionally, genetic distances between species were calculated using the K2P model in MEGA v7.0 [27] to further assess species relationships of the *placida*- and *schauffelbergeri*-groups.

### 2.4. Morphological Comparison

Male and female forewing lengths were measured to 0.5 mm precision using a ruler. The whole abdomen was then removed and placed into a 1.5 mL microcentrifuge tube, treated with 1 mL 10% sodium hydroxide (NaOH) solution to digest soft tissue for 1 h at 70 °C. The treated abdomen was then neutralized with 2% acetic acid and dissected in a water-filled Petri dish under a stereomicroscope to remove residual tissues, scales, and hair. The genitalia were transferred to 80% glycerol for 12 h to render them transparent.

Habitus images were taken using a Canon 7D camera in conjunction with a Canon MP-E 65 mm f/2.8 1–5X Macro Lens, and a Canon MT-24EX Macro Twin Lite Flash as a light source. Images of the genitalia were taken using a Canon G9 camera mounted on an Olympus CX31 microscope under reflection or transmission lighting. Zerene Stacker (version 1.04) was used for image stacking. All images were further adjusted and annotated using Adobe Photoshop CS6. The dissected genital structures were stored in pure glycerol in plastic centrifuge tubes labelled with detailed information on the specimens.

## 3. Results

### 3.1. Molecular Phylogenetic Analysis

**Figure 4:**
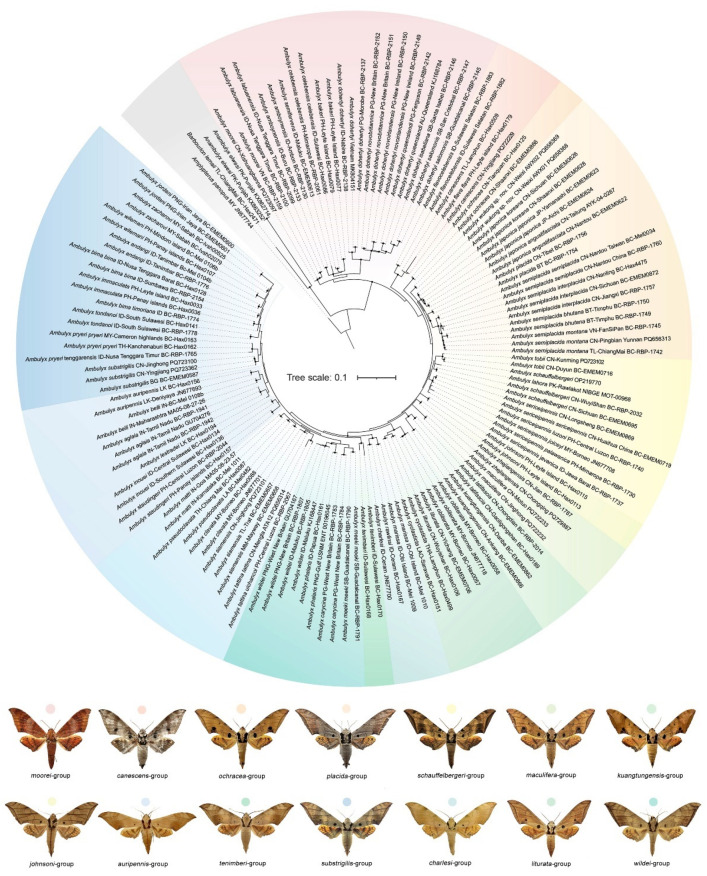
Maximum likelihood phylogenetic tree of the genus *Ambulyx* based on *cox1* DNA barcode sequences, reconstructed using IQ-TREE and rooted with *Barbourion lemaii*, *Amplypterus panopus*, and *Anambulyx elwesi* as outgroups. Nodes with SH-aLRT support values or ultrafast bootstrap (UFBoot2) values ≥90 are highlighted with black circles.

**Figure 5:**
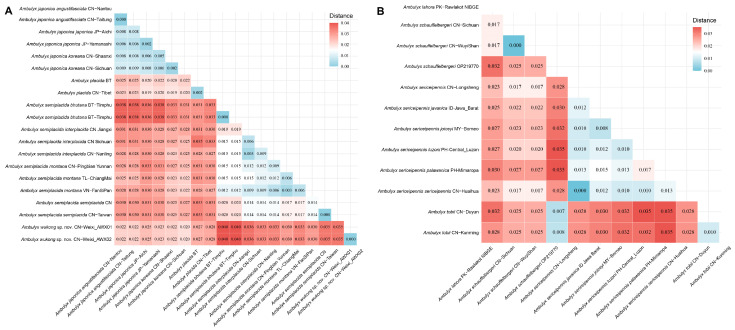
Kimura two-parameter (K2P) distances (in percentages) between taxa of the *placida*-group (**A**) and *schauffelbergeri*-group (**B**) calculated from DNA barcode sequences (*cox1*), with species identified as in the ggtree in Figure 4.

**Figure 6:**
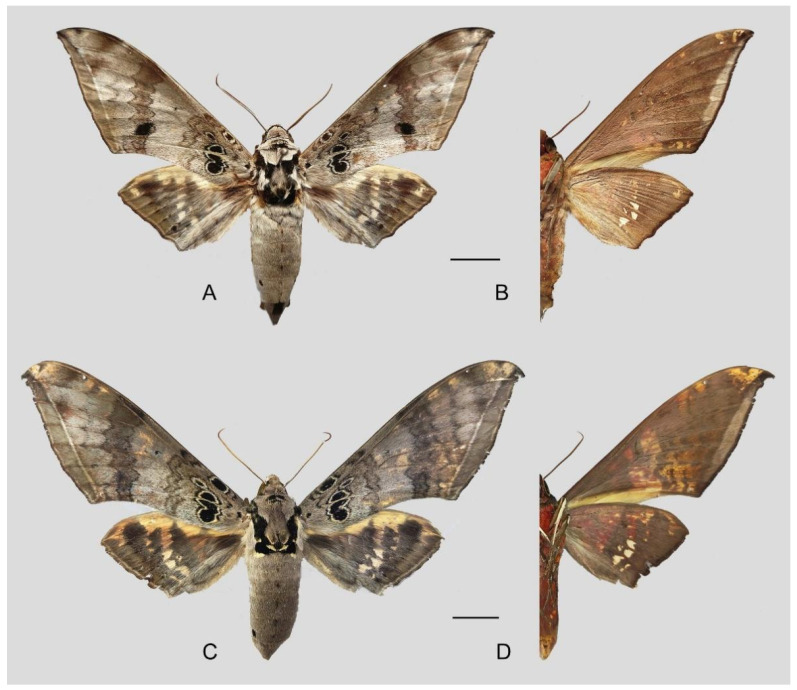
*Ambulyx canescens*. (**A**,**B**) Male, Yingjiang, Yunnan, China; (**C**,**D**) female, Chiang Mai, Thailand. Scale bar = 10 mm.

ModelFinder selected the best fit model as TIM2+F+I+R3. The IQTREE reconstruction converged well, and the phylogeny recovered the genus *Ambulyx* as monophyletic (Figure 4). Most species and species-groups were recovered as monophyletic with high bootstrap value but a few did have moderate to low bootstrap value, such as the *placida*- and *schauffelbergeri*-groups, probably due to the limited number of DNA barcode sequences (*cox1*) included in the analysis. The relationships among the taxa of the *placida*- and *schauffelbergeri*-groups are very close, which is shown by both the small Kimura 2-parameter (K2P) distances (Figure 5) (in *placida*-group within 0.04% and in *schauffelbergeri*-group within 0.035%). We included almost all species of *Ambulyx* in the phylogenetic analysis, rather than just the Chinese species, to maximize coverage and produce as comprehensive phylogenetic tree as possible. The species were grouped into 14 clades (Figure 4) as follows: 1. the *moorei*-group (*A*. *moorei*, *A*. *amboynensis*, *A*. *semifervens*, *A*. *dohertyi*, *A*. *bakeri*, *A*. *celebensis* and *A*. *labuanensis*); 2. the *canescens*-group, which is very close to the *moorei*-group (*A*. *canescens*, *A*. *flava* and *A*. *flavocelebensis*); 3. the *ochracea*-group (*A*. *ochracea* only, a very common species throughout E and SE Asia); 4. the *placida*-group (*A*. *wukong* sp. nov., *A*. *japonica*, *A*. *placida* and *A*. *semiplacida*); 5. the *schauffelbergeri*-group (*A*. *lahora*, *A*. *tobii*, *A*. *schauffelbergeri* and *A*. *sericeipennis*); 6. the *johnsoni*-group (*A*. *johnsoni* only, a species endemic to the islands of Philippines); 7. the *maculifera*-group (*A*. *maculifera* and *A*. *zhejiangensis*); 8. the *kuangtungensis*-group (*A*. *kuangtungensis* and *A*. *latifascia*); 9. the *liturata*-group (*A*. *liturata*, *A*. *obliterata* and *A*. *cyclasticta*); 10. the *charlesi*-group (*A*. *charlesi*, *A*. *marissa* and *A*. *rawlinsi*); 11. the *tenimberi*-group (*A*. *tenimberi* only, an endemic species on Sulawesi); 12. the *wildei*-group (*A*. *meeki*, *A*. *phalaris*, *A*. *carycina* and *A*. *wildei*); 13. the *auripennis*-group (*A*. *tattina*, *A*. *siamensis*, *A*. *clavata*, *A*. *pseudoclavata*, *A*. *matti*, *A*. *staudingeri*, *A*. *lestradei*, *A*. *inouei*, *A*. *belli*, *A*. *aglaia* and *A*. *auripennis*); and finally, 14. the *substrigilis*-group (*A*. *substrigilis*, *A*. *pryeri*, *A*. *tondanoi*, *A*. *zacharovi*, *A*. *bima*, *A*. *immaculata*, *A*. *andangi*, *A*. *wilemani* and *A*. *jordani*). Given the molecular evidence, we propose that *A. wukong* sp. nov. should be recognized as a good species that has a basal position within the *placida*-group (Figure 6A). We also maintain the subspecies of *A. sericeipennis* (Figure 6B), *A. dohertyi* and *A. bima* as valid, although some could be worthy of full species status, but this needs further study and new samples in the future analyses.

### 3.2. Taxonomic Review

#### 3.2.1. *Ambulyx canescens* (Walker, 1865) [灰带鹰翅天蛾]

*Basiana canescens* Walker, [1865]; List of the specimens of lepidopterous insects in the collection of the British Museum, 31 (suppl.): 38; **Type locality:** Cambodia.
  *Ambulyx argentata* Druce, 1882; Entomologist’s Monthly Magazine, 19: 17 [28].

**Diagnosis:** Male (Figure 6A,B): Head—grey; thorax—grey with wide black spots and a “V” shape black patch dorsally; abdomen—upperside greyish with a broken black line dorsally, tergum A8 with a dark patch dorsally and a tiny black spot on each side of segment 6 laterally. Forewing long, triangular with sharp apex, outer margin smooth, distal portion of inner margin slightly concave; upperside ground colour grey with scattered with pale brown scales, basal to medial area with three grey-brown zigzag lines, two separate and three connected to black circular subbasal spots edged in yellowish, medial area to submarginal area with a dark grey zigzag patch, a grey-black patch near the apex, subterminal line greyish-white, straight, meeting the inner margin just basad of the tornus, marginal area greyish-brown; underside—ground colour brown, with yellow spots dotted near apex and tornus, marginal area greyish. Hindwing— upperside dark greyish with two brown zigzag lines, a yellow broken patch in medial area and a thin black dotted line in submarginal area; underside—ground colour brown, a yellowish-white broken patch in medial area, with yellow spots near the apex.

Female (Figure 6C,D): Similar to the male but wings broader and pattern darker and more extensive; antennae are much more slender.

**Figure 7:**
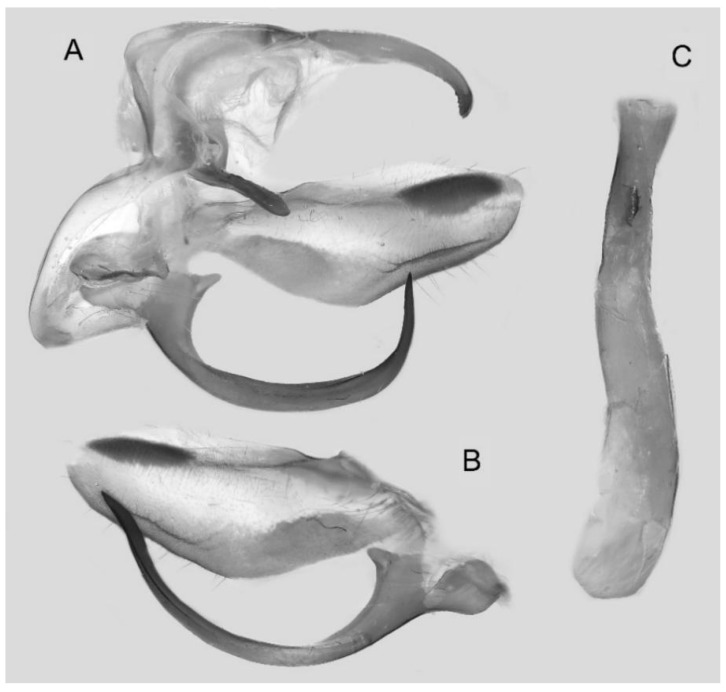
Male genitalia of *Ambulyx canescens*, Yingjiang, Yunnan, China. (**A**) Lateral view; (**B**) left valve; (**C**) phallus.

Male genitalia (Figure 7A–C): Uncus and gnathos form a typical Smerinthinae “bird-beak” structure. Uncus slender and downcurved, apical hook with tiny teeth. Gnathos straight, 1/2 as long as uncus, apex blunt. Valva long, tongue-shaped, with terminal part narrower than basal part, terminal part with a tongue-shaped patch of friction scales, apex blunt. Sacculus short, with a short, blunt dorsal lobe and a long, strongly upcurved hook-like harpe. Phallus long and slightly curved, anteriorly markedly narrower than posteriorly, with tiny spinules laterally near the apex.

**Figure 8:**
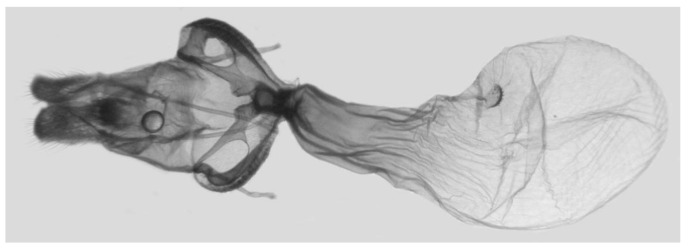
Female genitalia of *Ambulyx canescens*, Kao Yai, Thailand. © The Trustees of the Natural History Museum, London, UK (downloaded from Kitching, I.J. Sphingidae Taxonomic Inventory. http://sphingidae.myspecies.info/, accessed on accessed 20 April 2024.

Female genitalia (Figure 8): Anal papillae rounded. Lamella antevaginalis sclerotized, with two lateral annular structures that fuse to the bases of the anterior apophyses; lamella postvaginalis thicker and broad; antrum short and narrow. Ductus bursae tubular, membranous and broad. Corpus bursae membranous and oval, signum circular with tiny spinules around the edge.

**Figure 9:**
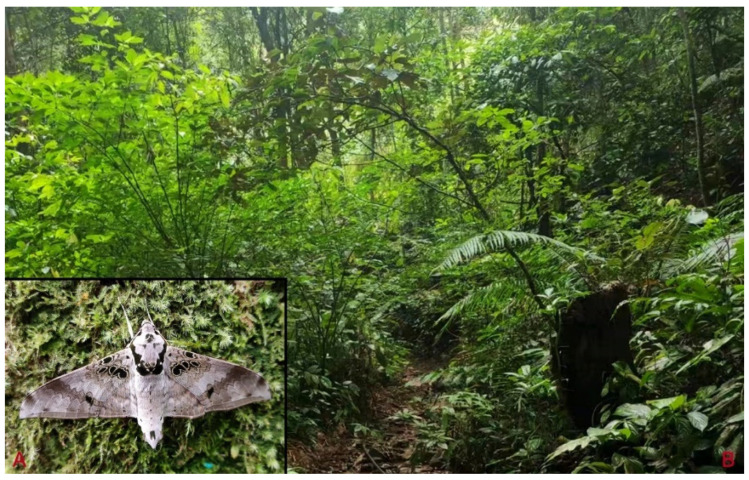
Habitat and living adult of *Ambulyx canescens*. (**A**) Male; (**B**) Yingjiang County, Yunnan, China.

**Biological notes:** This species was collected in mid-elevation subtropical forests, attracted to light at night or found at rest on plants during the day (Figure 9).

**Distribution:** China (W. Yunnan); India, Vietnam, Laos, Thailand, Cambodia, Malaysia, and Indonesia.

#### 3.2.2. *Ambulyx japonica japonica* Rothschild, 1894 [日本鹰翅天蛾指名亚种]

*Ambulyx japonica* Rothschild, 1894, Novit. Zool., 1: 87; **Type locality:** Kiushiu, Japan.

**Figure 10:**
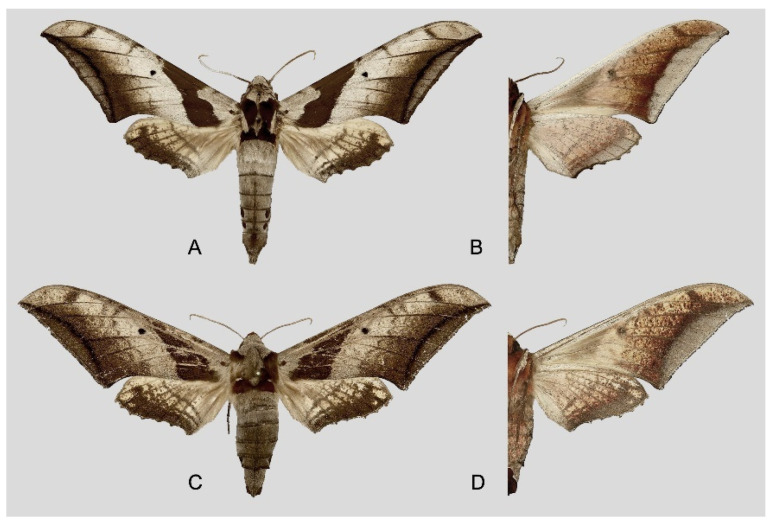
*Ambulyx japonica japonica*. (**A**,**B**) Male, Mikoboyama, Gumma, Japan; (**C**,**D**) female, Iruma, Saitama, Japan. © The Trustees of the Natural History Museum, London, UK (downloaded from Kitching, I.J. Sphingidae Taxonomic Inventory. http://sphingidae.myspecies.info, accessed on 20 April 2024.

**Diagnosis:** Male (Figure 10A,B): Head—grey; thorax—grey with wide “V” shaped black patch dorsally; abdomen—upperside greyish with a black dorsal line, a blackish green spot laterally on each side of segments 6 and 7. Forewing long, triangular with a sharp apex, outer margin smooth, distal part of inner margin strongly concave; upperside ground colour grey with scattered brown scales, a black basal spot, subbasal and basal costal spots enlarged and merged into a broad, blackish green, somewhat oblique transverse band, discal spot conspicuous, black, medial area to submarginal area with a brown zigzag line, a narrow brown patch near the apex, a strongly arched blackish subterminal line that ends at the tornus, with a large area of brown scales along its inner edge, marginal area grey; underside—almost the same as the upperside but colour and pattern paler and more diffuse. Hindwing—outer margin scalloped, upperside pale yellow with scattered tiny brown scales, a dark brown curved line in medial area and a black-brownish patch from the submarginal to the marginal area; underside—almost the same as the upperside but colour and pattern paler.

Female (Figure 10C,D): Similar to male but blackish green spot on each side of abdominal segment 6 only, wings broader and pattern darker and more extensive, antennae much slenderer.

**Figure 11:**
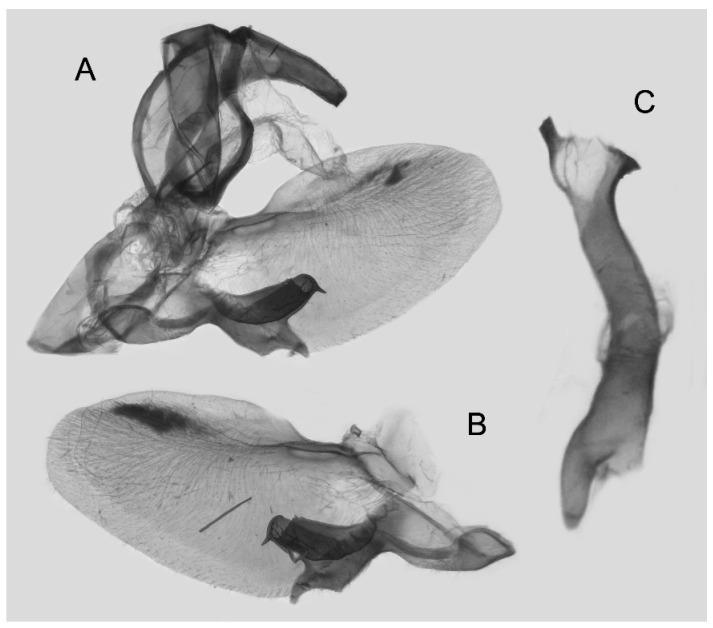
Male genitalia of *Ambulyx japonica japonica*, Namezawa Spa, Gumma, Japan. (**A**) Lateral view; (**B**) left valve; (**C**) phallus. © The Trustees of the Natural History Museum, London, UK (downloaded from Kitching, I.J. Sphingidae Taxonomic Inventory. http://sphingidae.myspecies.info/, accessed on 20 April 2024.

Male genitalia (Figure 11A–C): Uncus and gnathos form a typical Smerinthinae “bird-beak” structure. Uncus thick and downcurved, with an apical hook. Gnathos much shorter than uncus and strongly bilobed medially. Valva long, tongue-shaped, terminal part with a narrow tongue-shaped patch of friction scales, apex blunt. Sacculus with two curved lobes, the upper lobe thicker with apically downcurved apex, lower lobe broad with an apical downcurved, and blunt hook. Phallus long and strongly curved, anteriorly markedly wider than posteriorly, with a thick, sclerotized apical bar on one side and a sharp lateral tooth with tiny spinules on the other.

**Figure 12:**
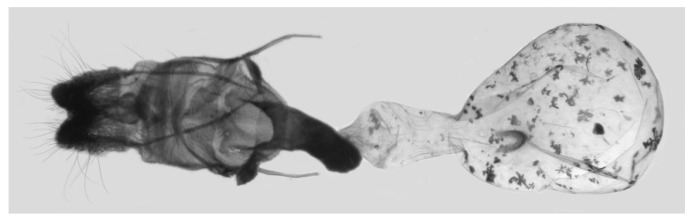
Female genitalia of *Ambulyx japonica japonica*, Iruma, Saitama, Japan. © The Trustees of the Natural History Museum, London, UK (downloaded from Kitching, I.J. Sphingidae Taxonomic Inventory. http://sphingidae.myspecies.info, accessed on 20 April 2024.

Female genitalia (Figure 12): Anal papillae rounded. Lamella antevaginalis with lateral sclerotized bars that loop round to fuse with the bases of the anterior apophyses; lamella postvaginalis broad; antrum thick and long. Ductus bursae short, membranous and broad. Corpus bursae membranous and oval, signum U-shaped with tiny marginal spinules.

**Figure 13:**
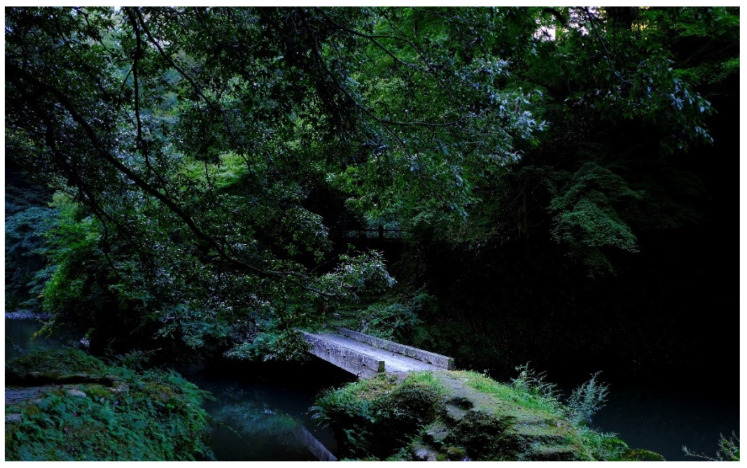
Habitat of *Ambulyx japonica japonica*, Kaga, Ishikawa-ken, Japan.

**Biological notes:** This species was collected in mid-elevation monsoon evergreen broad-leaved forest (Figure 13), attracted to light at night.

**Distribution:** Endemic to Japan.

#### 3.2.3. *Ambulyx japonica angustifasciata* Okano, 1959 [日本鹰翅天蛾台湾亚种]

*Oxyambulyx japonica angustifasciata* Okano, 1959; Rep. Gakigei Fac. Iwate Univ., 14: 40; **Type locality:** Puli—Wushe, Taiwan, China.

**Figure 14:**
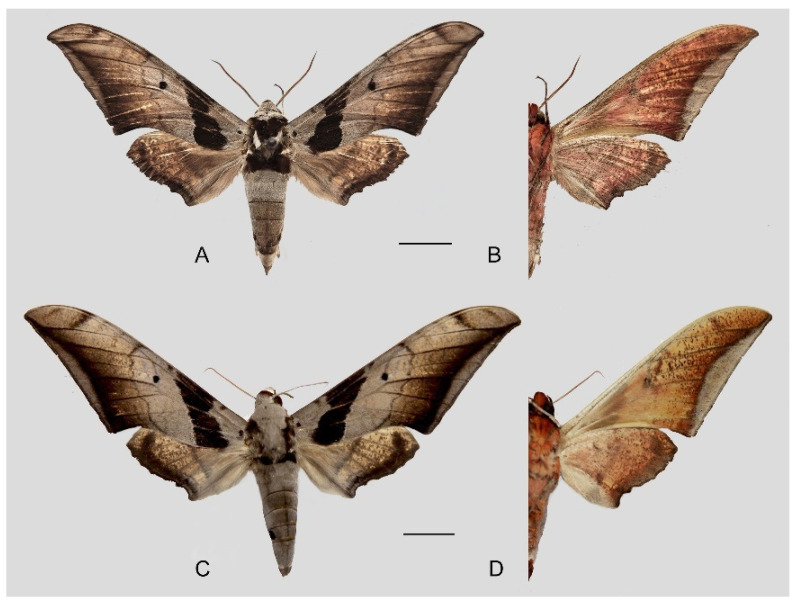
*Ambulyx japonica angustifasciata*. (**A**,**B**) Male, Donghe Township, Taitung County, Taiwan, China; (**C**,**D**) female, Puli, Nantou County, Taiwan, China. © Sphingidae Museum, Czech Republic. Scale bar = 10 mm.

**Diagnosis:** Male (Figure 14A,B): Similar to *A*. *j*. *japonica* but paler, the blackish green lateral spots on abdominal segment 7 much smaller, forewing narrower and longer, the black-green basal band on the upperside tending to be narrower.

Female (Figure 14C,D): Similar to the male, but a blackish green lateral spot on each side abdominal segment 6 only, wings broader and ground colour darker, antennae much slenderer.

**Figure 15:**
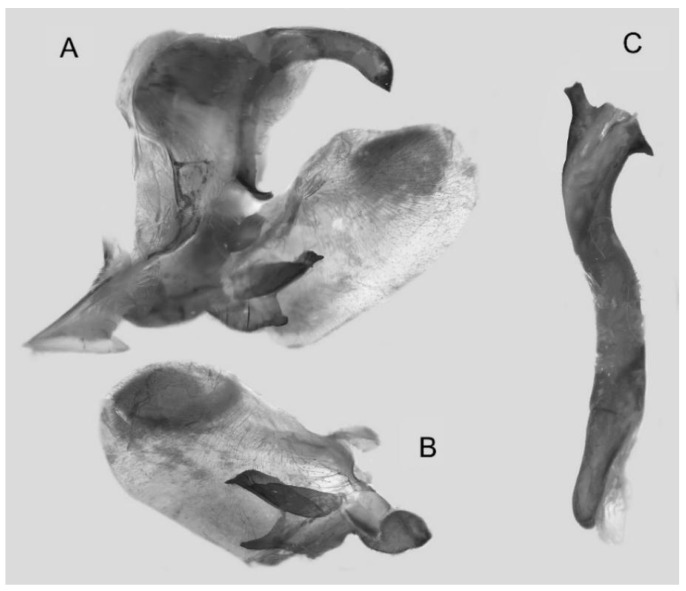
Male genitalia of *Ambulyx japonica angustifasciata*, Donghe Township, Taitung County, Taiwan, China. (**A**) Lateral view; (**B**) left valve; (**C**) phallus.

Male genitalia (Figure 15A–C): Similar to *A*. *j*. *japonica* but uncus thicker and more curved, upper sacculus lobe tending to be straighter, and apex of the lower lobe curved slightly upward. Phallus longer than *A*. *j*. *japonica*.

Female genitalia: Not examined.

**Figure 16:**
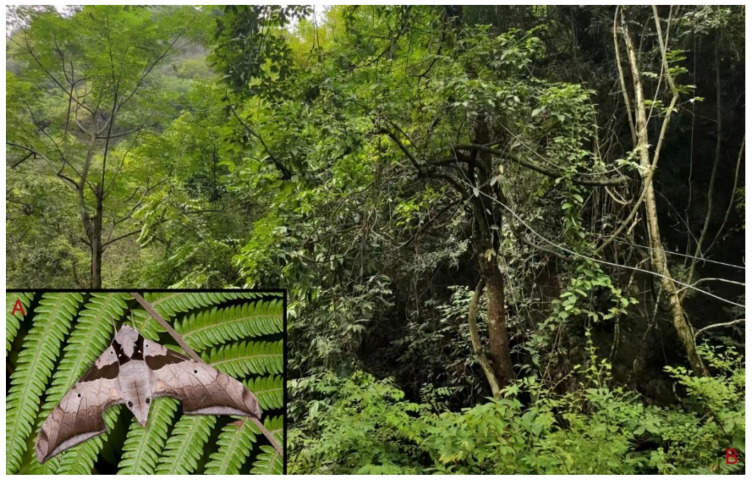
Habitat and living adult of *Ambulyx japonica angustifasciata*. (**A**) Female; (**B**) Taitung County, Taiwan, China.

**Biological notes:** This subspecies was collected in mid-elevation monsoon evergreen broad-leaved forest, attracted to light at night (Figure 16).

**Distribution:** Endemic to the island of Taiwan.

#### 3.2.4. *Ambulyx japonica koreana* Inoue, 1993 [日本鹰翅天蛾朝鲜亚种]

*Ambulyx japonica koreana* Inoue, 1993; Insecta Koreana, 10: 50; **Type locality:** Mt. Odae, Kangwon Province, South Korea.

**Figure 17:**
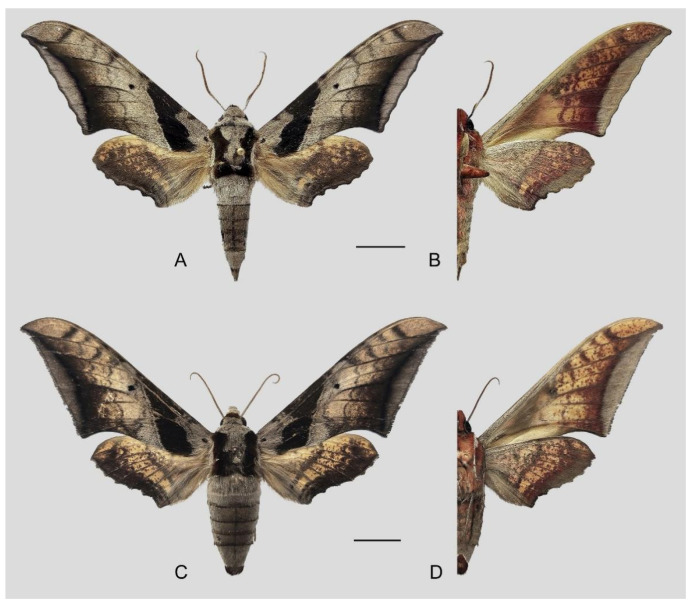
*Ambulyx japonica koreana*. (**A**,**B**) Male, Wuxi County, Chongqing, China; (**C**,**D**) female, Mentougou, Beijing, China. Scale bar = 10 mm.

**Diagnosis:** Male (Figure 17A,B): Similar to *A*. *j*. *japonica* but larger and ground colour tending to be more yellow, the blackish green spot with on the side of the segment 7 much smaller, forewing broader.

Female (Figure 17C,D): Similar to male but blackish green spot with only on each side of the segment 6, wings broader and ground colour darker, antennae much slenderer.

**Figure 18:**
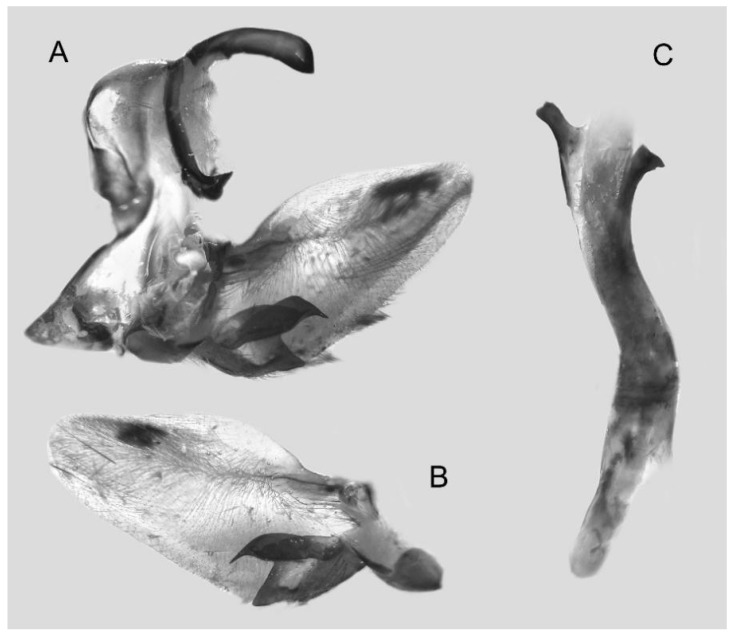
Male genitalia of *Ambulyx japonica koreana*, Changyang County, Hubei, China. (**A**) Lateral view; (**B**) left valve; (**C**) phallus.

Male genitalia (Figure 18A–C): Similar to *A*. *j*. *japonica* but uncus more strongly curved, upper lobe of sacculus tending to be broader and straighter and lower lobe reduced with a broad apical tooth.

**Figure 19:**
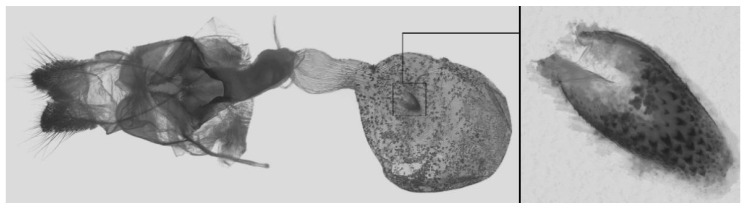
Female genitalia of *Ambulyx japonica koreana*, Wuxi County, Chongqing, China.

Female genitalia (Figure 19): Similar to *A*. *j*. *japonica* but signum circular and leaf-shaped, covered with tiny teeth.

**Figure 20:**
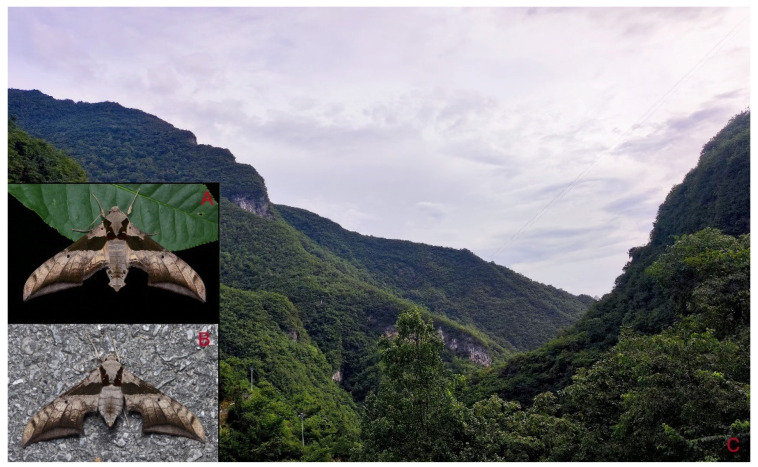
Habitat and living adults of *Ambulyx japonica koreana*. (**A**) Male; (**B**) female; (**C**) Wuxi County, Chongqing, China.

**Biological notes:** This subspecies was collected in mid- to high elevation monsoon evergreen broad-leaved forest, attracted to light at night (Figure 20).

**Distribution:** China (Jilin, Liaoning, Beijing, Hebei, Tianjin, Shaanxi, Henan, Anhui, Hubei, Jiangxi, Sichuan, Guizhou, and Chongqing); North Korea, South Korea.

#### 3.2.5. *Ambulyx wukong* Jiang & Kitching **sp. nov.** [悟空鹰翅天蛾]

Article registration: urn:lsid:zoobank.org:pub:C35EA7C0-4F7A-4572-A204-BDBDB36F8E2E

Species registration: urn:lsid:zoobank.org:act:3B8A06A9-D158-4B8F-BD47-44F4F8F02810

**Type data: HOLOTYPE:** ♂, Tacheng (2350 m), Weixi County, Yunnan, China, 2023 V-13, local collector leg. [KIZAS 0133744]. **PARATYPES:** 4♂♂, Tacheng (2510 m), Weixi County, Yunnan, China, 2023 V-17, local collector *leg*. [ZHJ]; 2♂♂, ♀, Tacheng (2510 m), Weixi County, Yunnan, China, 2024 V-12, local collector *leg*. [ZHJ].

**Figure 21:**
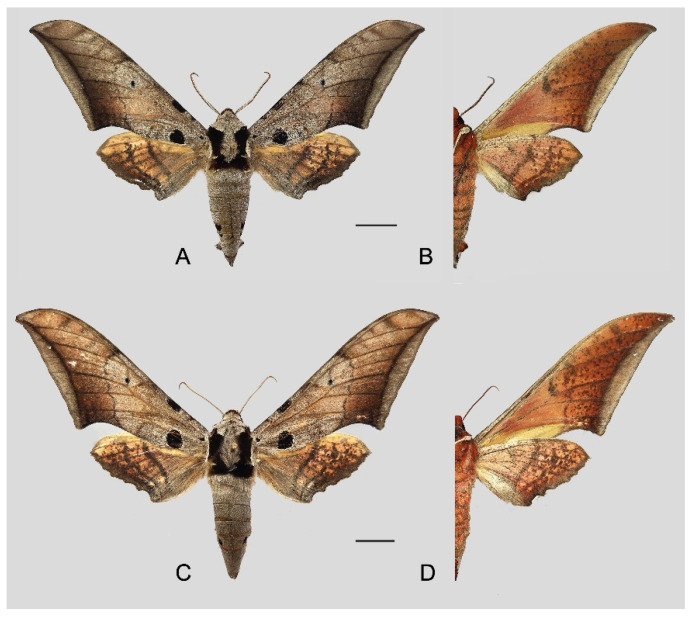
*Ambulyx wukong* **sp. nov.** (**A**,**B**) Male, **HOLOTYPE**, Tacheng, Weixi County, Yunnan, China; (**C**,**D**) Female, **PARATYPE**, Tacheng, Weixi County, Yunnan, China. Scale bar = 10 mm.

**Diagnosis:** Male (Figure 21A,B): Similar to *A. placida* but larger and the colour tending to be darker. Forewing broader and wider, upperside ground colour brownish-grey, the subbasal black spots much larger than in *A. placida*, black discal spot conspicuous, medial area to submarginal area with scattered greyish-brown scales, a black line with brownish-black scales along its margins runs from apex to tornus, marginal area grey; underside—almost the same as the upperside but ground colour ochre, with dense scattered greyish-black scaling. Hindwing—upperside yellow with dense scattered black scales, marginal area greyish-yellow; underside—almost the same as the upperside but ground colour ochre and pattern paler.

Female (Figure 21C,D): Similar to the male, but wings broader and ground colour paler, antennae much slenderer.

**Figure 22:**
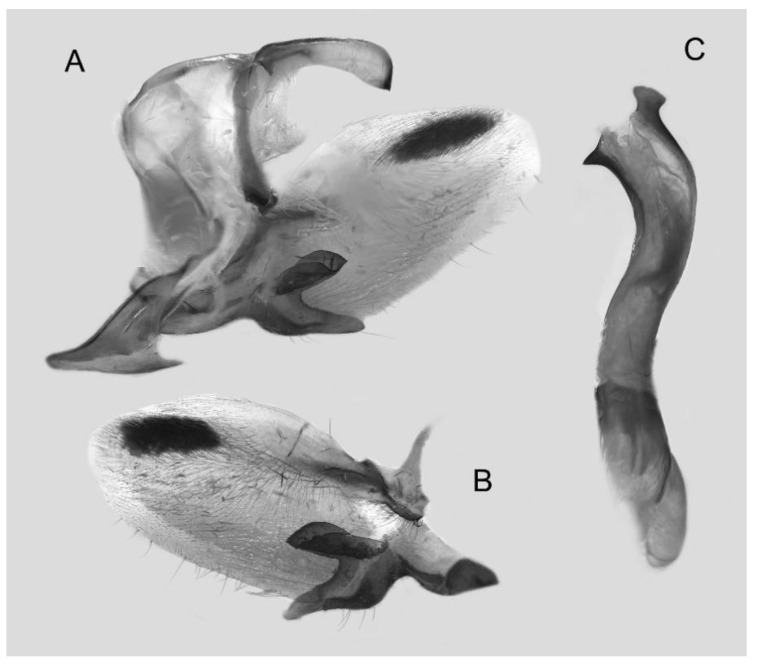
PARATYPE, male genitalia of *Ambulyx wukong* **sp. nov.**, Tacheng, Weixi County, Yunnan, China. (**A**) Lateral view; (**B**) left valve; (**C**) phallus.

Male genitalia (Figure 22A–C): Similar to *A. placida* but gnathos bilobed, the two lobes of the sacculus slightly shorter and broader, and on the phallus the sclerotized posterior lobe is sharp and shorter and the tooth on the opposite side is lateral rather than terminal.

**Figure 23:**
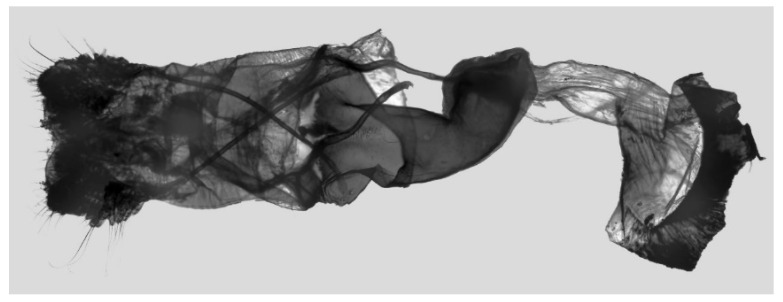
PARATYPE, female genitalia of *Ambulyx wukong* **sp. nov.**, Tacheng, Weixi County, Yunnan, China.

Female genitalia (Figure 23): Similar to *A. placida* but antrum thicker and broad.

**Figure 24:**
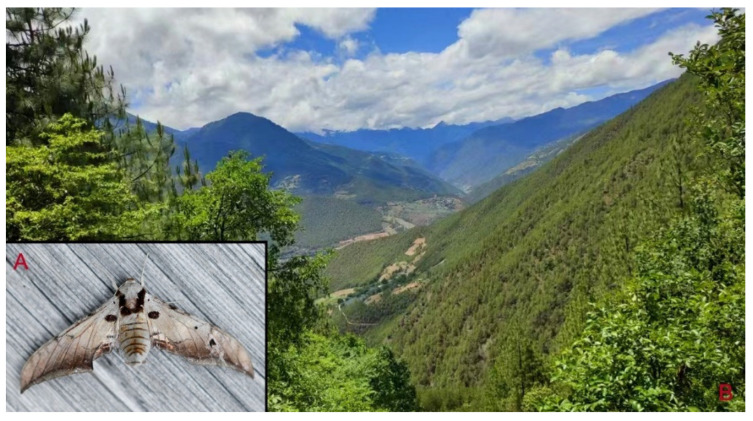
Habitat and living adult of *Ambulyx wukong* **sp. nov.** (**A**) Male; (**B**) Weixi County, Yunnan, China.

**Biological notes:** This species was collected in high elevation monsoon evergreen broad-leaved forest, attracted to light at night (Figure 24).

**Distribution:** Currently known only from NW Yunnan.

**Note:** The name of new species derives from The Great Sage, Heaven’s Equal, Sun Wukong, a character from the famous Chinese myth, Journey to the West. It is to be treated as a noun in apposition.

#### 3.2.6. *Ambulyx kuangtungensis* (Mell, 1922) [华南鹰翅天蛾]

*Oxyambulyx kuangtungensis* Mell, 1922; Dt. ent. Z., 1922: 114; **Type locality:** northwest Kwangtung, China.
  *Oxyambulyx kuangtungensis formosana* Clark, 1936; Proceedings of the New England Zoological Club, 15: 73 [29].
  *Oxyambulyx kuangtungensis hoenei* Mell, 1937; Deutsche Entomologische Zeitschrift, Berlin, 1937: 4 [30].
  *Oxyambulyx kuangtungensis melli* Gehlen, 1942; Entomologische Zeitschrift, 56: 73 [31].
  *Oxyambulyx takasago* Okano, 1964; Tohoku Konchu Kenkyu, 1: 41 [17].
  *Ambulyx adhemariusa* Eitschberger, Bergmann & Hauenstein, 2006; Atalanta, 37: 483 [32].

**Figure 25:**
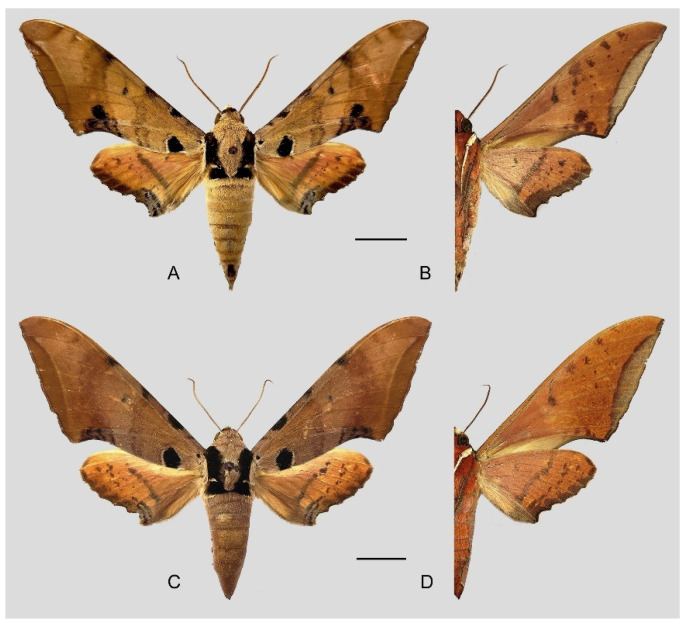
*Ambulyx kuangtungensis*. (**A**,**B**) Male, Maolan Nature Reserve, Libo, Guizhou, China; (**C**,**D**) female, Yintiaoling Nature Reserve, Wuxi County, Chongqing, China. Scale bar = 10 mm.

**Diagnosis:** Male (Figure 25A,B): Very similar to *A. ochracea* but generally smaller. Forewing upperside subbasal and basal costal spots usually separate but can be enlarged and merged to form a transverse band that looks similar to that seen in *A. latifascia*; medial area with more scattered brown scales and a conspicuous curved and broken, brown postmedial line; subterminal line weak, greyish, its greenish-yellow edge obviously fading before reaching the tornus; when present, the blackish-green spot in the submarginal area between veins CuA_1_ and CuA_2_ generally smaller than in *A. ochracea*; marginal area brown; underside basal to medial area with scattered conspicuous pink scales. Hindwing upperside with conspicuous dark brown scattered scales, basal to medial area covered with pink scales.

Female (Figure 25C,D): Similar to male but wings broader and forewing ground colour ochraceous, patterns faded, antennae much slenderer.

**Figure 26:**
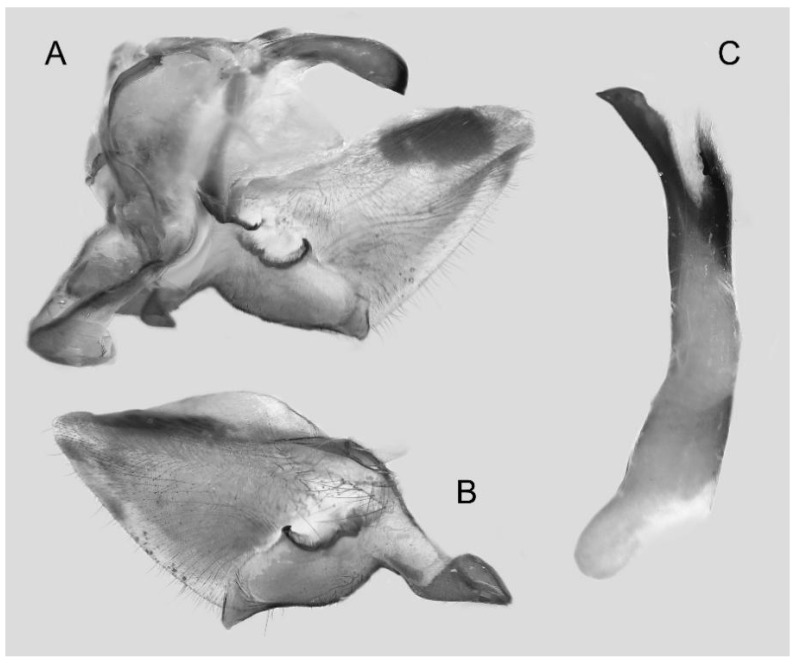
Male genitalia of *Ambulyx kuangtungensis*, Mt. Tianmushan, Zhejiang, China. (**A**) Lateral view; (**B**) left valve; (**C**) phallus.

Male genitalia (Figure 26A–C): Similar to *A. ochracea*, with patch of friction scales on valve narrower. Sacculus broader; dorsal margin with an irregularly excavated concavity ending in a distal recurved hook; harpe broader, triangular, and somewhat downcurved. Phallus shorter and thicker than *A. ochracea*, sclerotized posterior lobe strongly curved and apically sharply pointed, with tiny teeth laterally.

**Figure 27:**
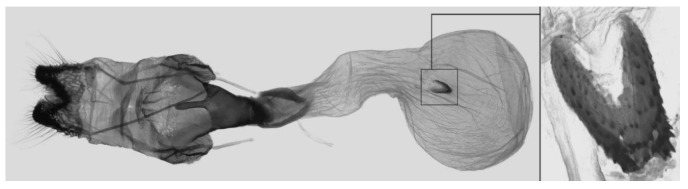
Female genitalia of *Ambulyx kuangtungensis*, Yingshan County, Hubei, China.

Female genitalia (Figure 27): Similar to those of *A. maculifera*. Antrum short, funnel- shaped, posterior margin with a long, slender, finger-like lobe centrally. Ductus bursae short. Signum tongue-shaped, covered with tiny teeth, and a triangle membranous gap centrally and posteriorly.

**Figure 28:**
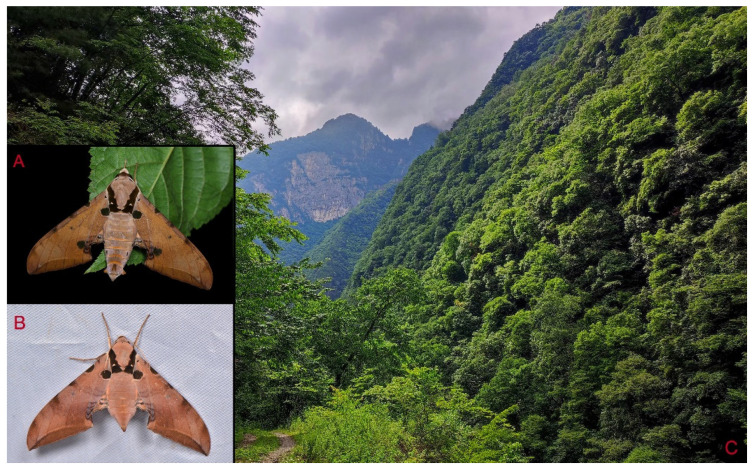
Habitat and living adults of *Ambulyx kuangtungensis*. (**A**) Male; (**B**) female; (**C**) Wuxi County, Chongqing, China.

**Biological notes:** This species was collected in low to mid-elevation monsoon evergreen broad-leaved forest, attracted to light at night (Figure 28).

**Distribution:** China (Beijing, Hebei, Shaanxi, Hubei, Sichuan, Gansu, Henan, An-hui, Jiangsu, Zhejiang, Fujian, Taiwan, Jiangxi, Hunan, Chongqing, Guangdong, Guangxi, Yunnan, Xizang, Guizhou); Myanmar, Thailand, Laos, and Vietnam.

#### 3.2.7. *Ambulyx latifascia* Brechlin & Haxaire, 2014 [连斑鹰翅天蛾]

*Ambulyx latifascia* Brechlin & Haxaire, 2014; Entomo-Satsphingia, 7(2): 46; **Type locality:** Hutiaoxia, Northwestern Yunnan, China.

**Figure 29:**
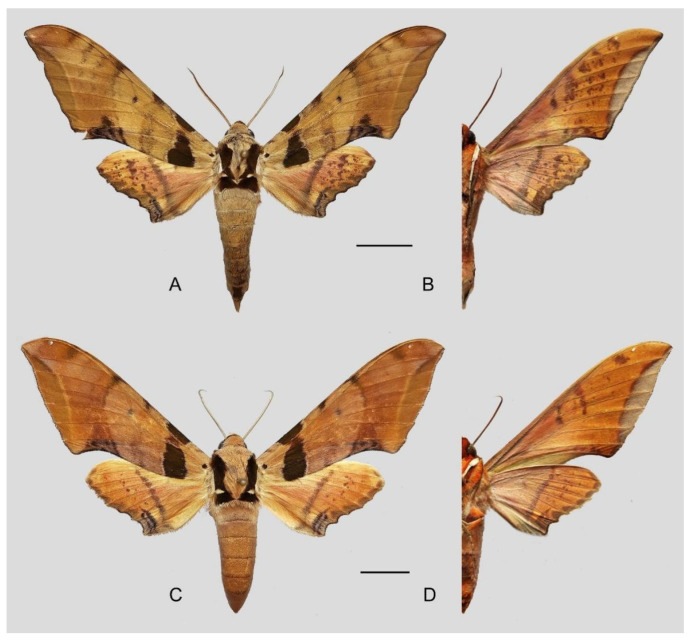
*Ambulyx latifascia*. (**A**,**B**) Male, Hutiaoxia, Lijiang, Yunnan, China; (**C**,**D**) female, Hutiaoxia, Lijiang, Yunnan, China. Scale bar = 10 mm.

**Diagnosis:** Male (Figure 29A,B): Very similar to *A*. *kuangtungensis*. Forewing with greenish-black subbasal spot and costal spot enlarged, approaching each another or even fused into a single transverse band (as in some specimens of *A*. *kuangtungensis*); blackish green spot on submarginal area between veins CuA1 and CuA2 with generally smaller than in *A. kuangtungensis*. Forewing and hindwing undersides with scattered and more conspicuous pinkish-orange scales.

Female (Figure 29C,D): Similar to male but wings broader and forewing ground colour ochraceous and pattern paler, brown curved dotted postmedial line darker, antennae much slenderer.

**Figure 30:**
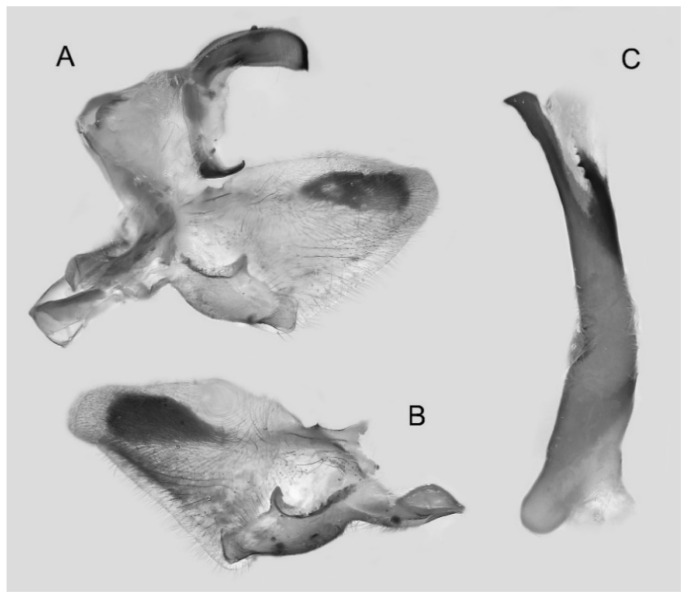
Male genitalia of *Ambulyx latifascia*, Renhe Village, Lijiang, Yunnan, China. (**A**) Lateral view; (**B**) left valve; (**C**) phallus.

Male genitalia (Figure 30A–C): Very similar to *A. kuangtungensis*. Harpe broader, much blunter and more shovel-shaped; concavity on dorsal edge with a broader and blunter distal tooth than that in *A. kuangtungensis*. Phallus posterior lobe slender and not as curved as in *A. kuangtungensis*, with more conspicuous tiny teeth laterally.

**Figure 31:**
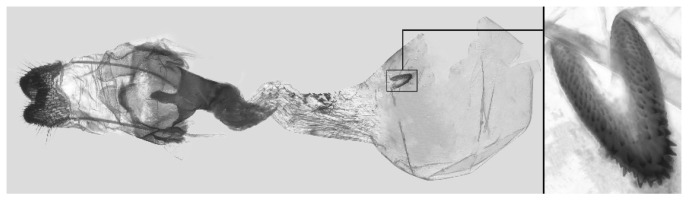
Female genitalia of *Ambulyx latifascia*, Hutiaoxia, Lijiang, Yunnan, China.

Female genitalia (Figure 31): Similar to those of *A. kuangtungensis*. Antrum broader, central finger-like lobe on the posterior margin shorter and thicker. Signum tongue-shaped and covered with tiny teeth, narrower than in *A. kuangtungensis*.

**Figure 32:**
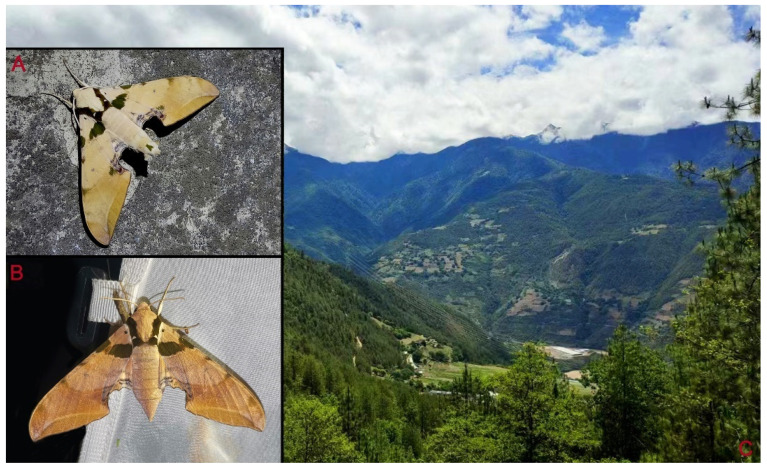
Habitat and living adults of *Ambulyx latifascia*. (**A**) Male; (**B**) female; (**C**) Weixi County, Yunnan, China.

**Biological notes:** This species was collected in high elevation monsoon evergreen broad-leaved forest, attracted to light at night (Figure 32).

**Distribution:** Currently known from SW China (south Sichuan, northwestern Yunnan).

#### 3.2.8. *Ambulyx liturata* Butler, 1875 [栎鹰翅天蛾]

*Ambulyx liturata* Butler, 1875; Proc. zool. Soc. Lond., 1875: 250; **Type locality:** Not stated.
  *Ambulyx rhodoptera* Butler, 1875; Proc. zool. Soc. Lond., 1875: 251 [7].

**Figure 33:**
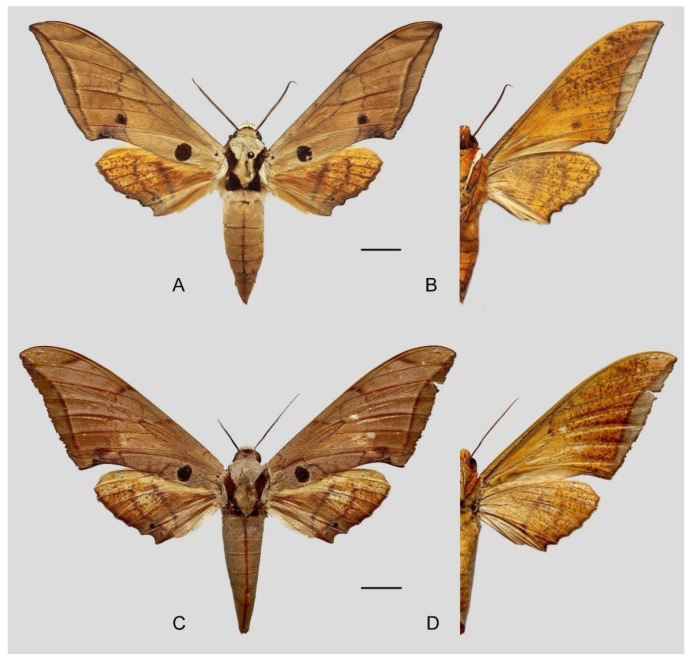
*Ambulyx liturata*. (**A**,**B**) Male, Mt. Jianfengling, Ledong County, Hainan; (**C**,**D**) female, Youxi County, Sanming, Fujian, China. Scale bar = 10 mm.

**Diagnosis:** Male (Figure 33A,B): Similar to *A. substrigilis*. Forewing upperside pattern paler and more indistinct, especially the blackish-brown zigzag lines and patches, subbasal spot generally larger than in *A. substrigilis*, subterminal line narrow, blackish, arched and fading before reaching the tornus, a conspicuous black-greenish spot on the submarginal area between veins CuA_1_ and CuA_2_; underside subterminal line indistinct, brownish. Hindwing pattern paler and more indistinct than in *A. substrigilis* with fewer scattered black scales, basal patch small, indistinct and brownish, rather than large and black.

Female (Figure 33C,D): Similar to male but wings broader and forewing ground colour slightly ochraceous, pattern more indistinct, antennae much slenderer.

**Figure 34:**
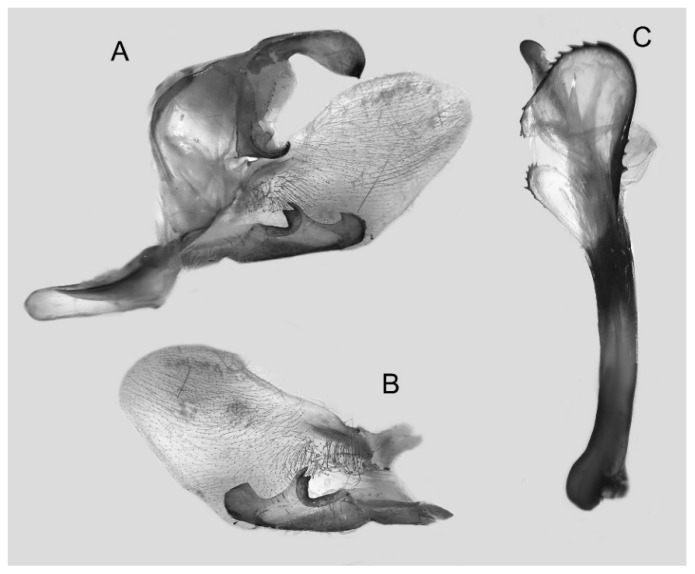
Male genitalia of *Ambulyx liturata*, Mt. Tianmushan, Zhejiang, China. (**A**) Lateral view; (**B**) left valve; (**C**) phallus.

Male genitalia (Figure 34A–C): Similar to *A. substrigilis*. Sacculus slender, dorsal margin a heavy curved, sharply pointed bifurcate lobe, harpe broader, spatulate, with a short but sharp dorsal hook. Phallus slenderer and more curved than *A. substrigilis*, posterior lobe much thicker and apically blunt, with two semi-circular serrated lobes laterally, one much larger than the other.

**Figure 35:**
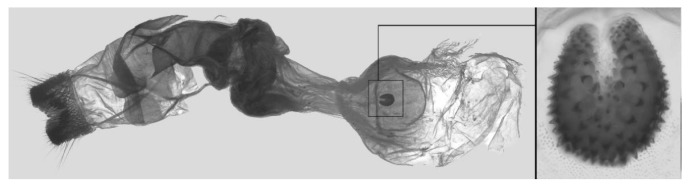
Female genitalia of *Ambulyx liturata*, Youxi County, Sanming, Fujian, China.

Female genitalia (Figure 35): Similar to *A. substrigilis* but ductus bursae shorter. Signum oval with a conspicuous gap in the central area.

**Figure 36:**
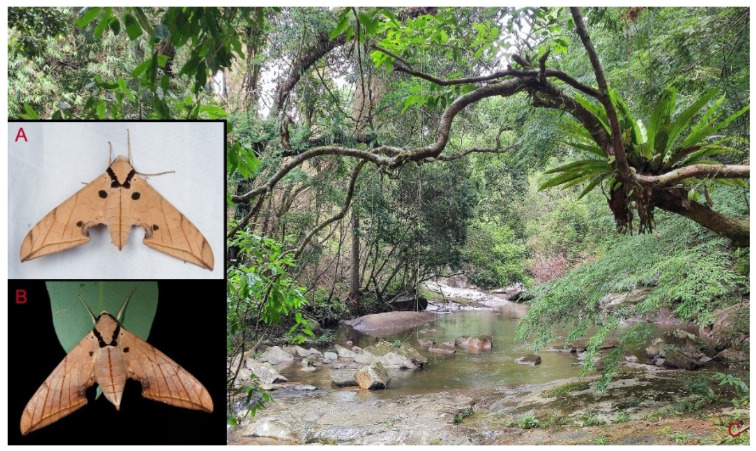
Habitat and living adults of *Ambulyx liturata*. (**A**) Male; (**B**) female; (**C**) Lingshui, Hainan, China.

**Biological notes:** This species was collected in low to mid-elevation monsoon evergreen broad-leaved forest and subtropical forests, attracted to light at night (Figure 36).

**Distribution:** China (Anhui, Zhejiang, Hubei, Sichuan, Yunnan, Xizang, Hunan, Fujian, Chongqing, Guangdong, Hongkong, Guangxi, and Hainan); Nepal, Bhutan, India, Myanmar, Thailand, Laos, and Vietnam.

#### 3.2.9. *Ambulyx maculifera* Walker, 1866 [杂斑鹰翅天蛾]

*Ambulyx maculifera* Walker, 1866; List of the specimens of lepidopterous insects in the collection of the British Museum, 35: 185; **Type locality:** Darjiling, West Bengal, India.
  *Ambulyx consanguis* Butler, 1881; Illustrations of typical specimens of Lepidoptera Heterocera in the collection of the British Museum, 5: 11 [33].

**Figure 37:**
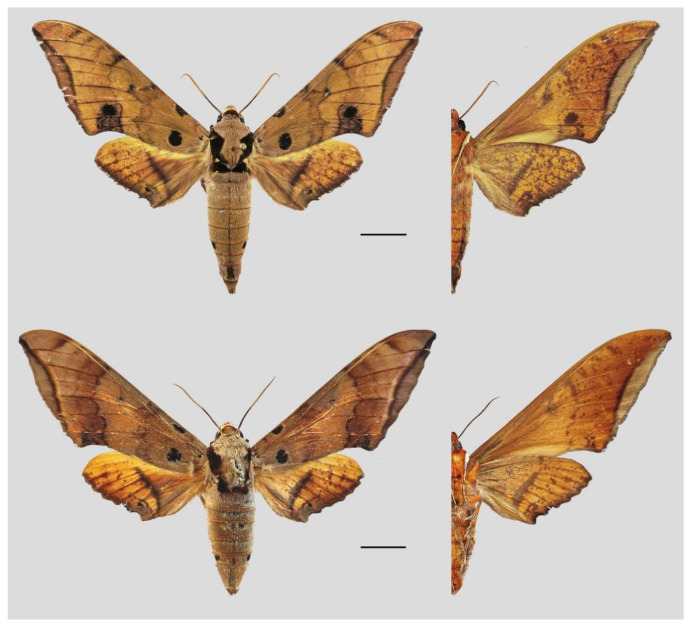
*Ambulyx maculifera*. (**A**,**B**) Male, Pu’er, Yunnan, China; (**C**,**D**) female, Xima Town, Yingjiang County, Yunnan, China. Scale bar = 10 mm.

**Diagnosis:** Male (Figure 37A,B): Similar to *A. s. sericeipennis* but ground colour tends to be more brownish-yellow. Forewing narrower than *A. s. sericeipennis*, upperside ground colour brownish-yellow, black-green subbasal spot generally larger than in *A. s. sericeipennis*, basal to medial area with conspicuous black-brown zigzag lines, subterminal line blackish, strongly arched, inner edge narrowly highlighted in yellow, ending on the outer margin rather than at the tornus or on the inner margin; the blackish-green spot in the submarginal area between veins CuA_1_ and CuA_2_ generally larger than in *A. s. sericeipennis*; underside marginal area grey.

Female (Figure 37C,D): Similar to male but wings broader and forewing ground colour ochraceous, subterminal line broader and yellowish-green, marginal area ochraceous, antennae much slenderer.

**Figure 38:**
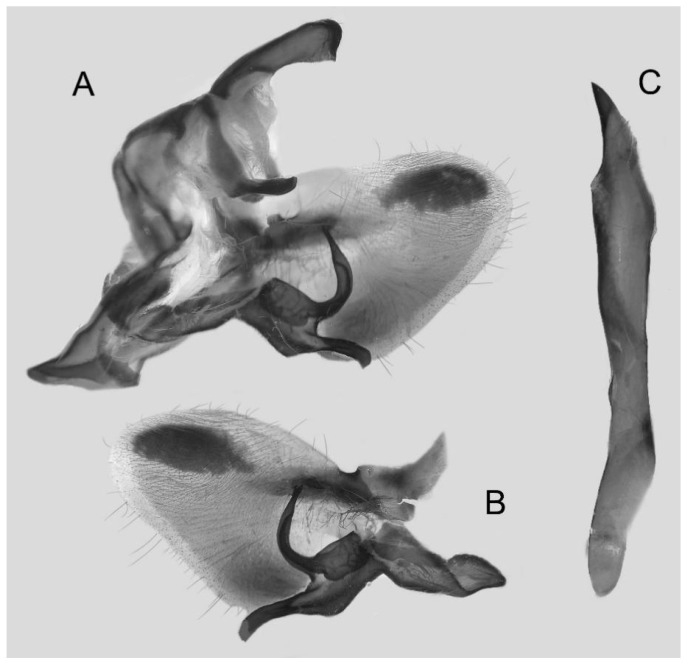
Male genitalia of *Ambulyx maculifera*, Motuo County, Yunnan, China. (**A**) Lateral view; (**B**) left valve; (**C**) phallus.

Male genitalia (Figure 38 A–C): Uncus and gnathos similar to *A. s. sericeipennis*. Sacculus with a long, strongly recurved hook on the dorsal margin, harpe thicker and curved downward with a blunt apex. Phallus slender, posterior lobe sharply pointed apically, with tiny teeth dorsally and ventrally.

**Figure 39:**
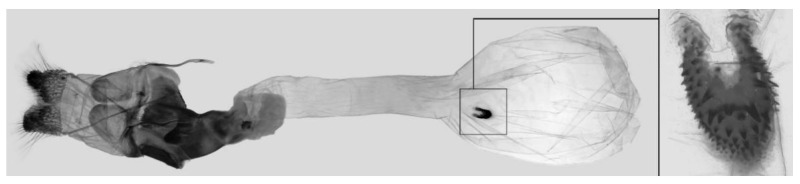
Female genitalia of *Ambulyx maculifera*, Xima Town, Yingjiang County, Yunnan, China.

Female genitalia (Figure 39): Similar to those of *A. s. sericeipennis*. Ductus bursae tub-ular, membranous and long. Signum oval with denser spines.

**Figure 40:**
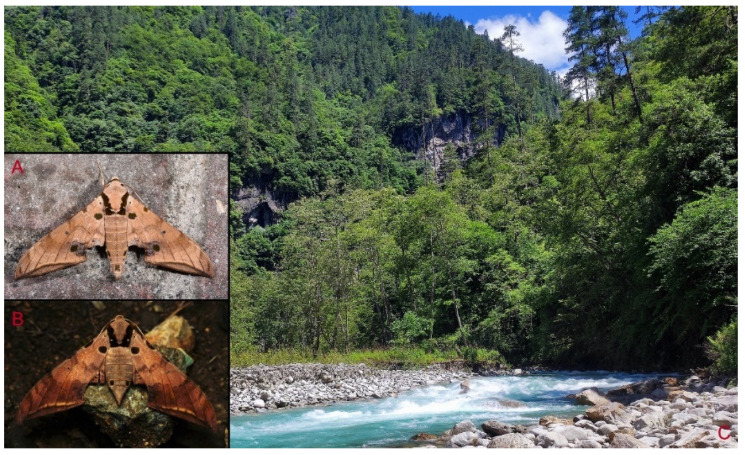
Habitat and living adults of *Ambulyx maculifera*. (**A**) Male; (**B**) female; (**C**) Dulongjiang, Yunnan, China.

**Biological notes:** This species was collected in mid- to high elevation monsoon evergreen broad-leaved forest, attracted to light at night (Figure 40).

**Distribution:** China (Yunnan, Xizang, Guizhou); Pakistan, Nepal, Bhutan, India, Myanmar, and Vietnam.

#### 3.2.10. *Ambulyx moorei* Moore, 1858 [摩尔鹰翅天蛾]

*Ambulyx moorei* Moore, 1858; in Horsfield & Moore, Cat. Lepid. Ins. Mus. East India Company, 1: 266; **Type locality:** Java, Indonesia.

*Ambulyx subocellata* C. Felder & R. Felder, 1874; Lepidoptera. Heft IV. Atlas der Heterocera Sphingidae-Noctuidae pl. 76, Figure 3 [34].
*Ambulyx turbata* Butler, 1875; Proc. zool. Soc. Lond., 1875: 252 [7].
*Ambulyx thwaitesii* Moore, 1882; Lep. Ceylon, 2: 11 [35].
*Ambulyx nubila* Huwe, 1895; Verzeichniss der von Hans Fruhstorfer während seines Aufenthalts auf Java in den Jahren 1891 bis 1893 erbeuteten Sphingiden, 40: 366 [36].
*Oxyambulyx moorei chinensis* Clark, 1922; Proceedings of the New England Zoological Club, 8: 7 [37].

**Figure 41:**
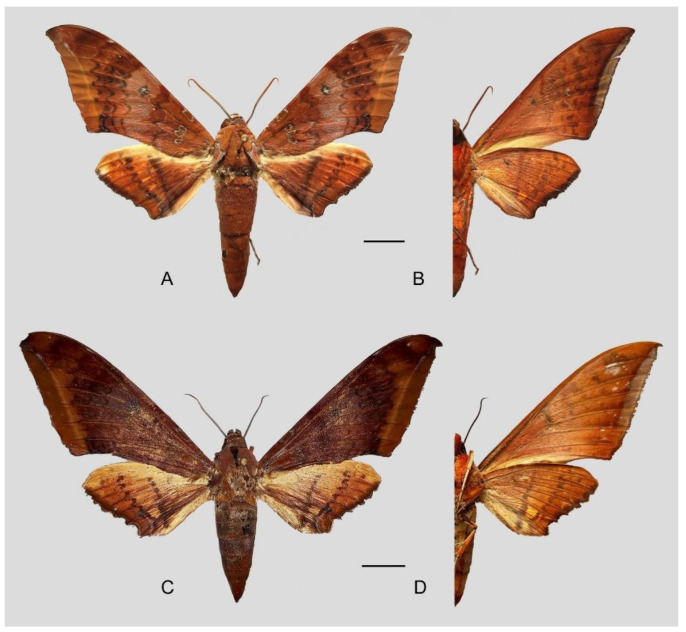
*Ambulyx moorei*. (**A**,**B**) Male, Mt. Jianfengling, Ledong County, Hainan, China; (**C**,**D**) female, Xima Town, Yingjiang County, Yunnan, China. Scale bar = 10 mm.

**Diagnosis:** Male (Figure 41A,B): Head—reddish-brown; thorax—amber with blackish lateral patches; abdomen—upperside amber, a blackish green spot edged with white on each side of the segment 6 laterally. Forewing shape and pattern similar to *A. canescens*, but upperside ground colour amber with scattered brown scales, basal band comprising one large and separate and three smaller and connected black circular spots, all edged in greyish, medial area greyish-pink, discal spot white, medial area to submarginal area with three zigzag dark brown lines, a deep brown patch near the apex, a white line runs from the apex to tornus, marginal area yellowish-brown; underside—ground colour amber, with dark brown spots and zigzag dotted lines, the line running from the apex to tornus dark brown, marginal area greyish. Hindwing— upperside ground colour amber, a black-brown line in the medial area and a black-brown zigzag line in the submarginal area, marginal area dark brown, tornal area greyish-yellow; underside—almost the same as the upperside but pattern paler.

Female (Figure 41C,D): Similar to the male, but wings broader and ground pattern darker and more extensive, antennae much slenderer.

**Figure 42:**
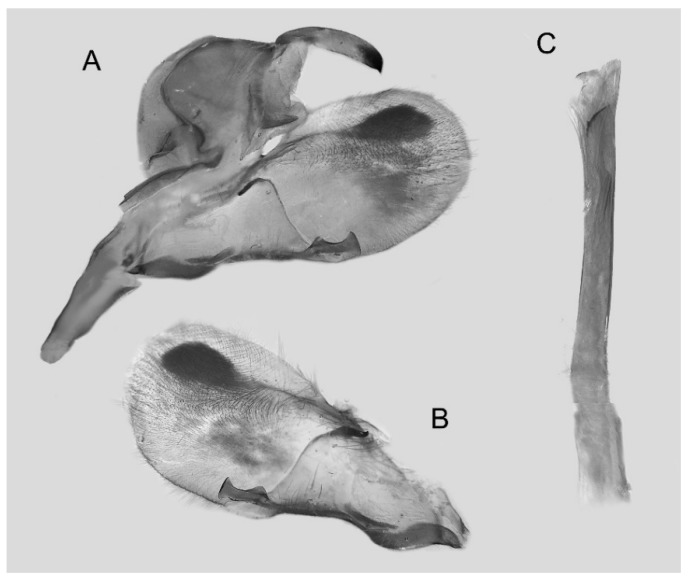
Male genitalia of *Ambulyx moorei*, Mengla County, Yunnan, China. (**A**) Lateral view; (**B**) left valve; (**C**) phallus.

Male genitalia (Figure 42A–C): Uncus and gnathos form typical Smerinthinae “bird-beak” structure. Uncus slender and curved downward, apical hook sharp. Gnathos much shorter than uncus. Valva tongue-shaped, terminal part wider than basal part, with a tongue-shaped patch of friction scales, apex blunt. Sacculus basally broad, dorsal margin with a broad, blunt triangular lobe, harpe strongly narrowed into a wavy hook. Phallus long and straight, posterior lobe with tiny spinules laterally.

**Figure 43:**
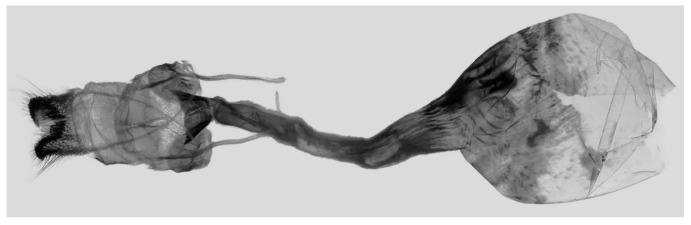
Female genitalia of *Ambulyx moorei*, Xima Town, Yingjiang County, Yunnan.

Female genitalia (Figure 43): Anal papillae rounded. Lamella antevaginalis sclerotized, annular; lamella postvaginalis broad; antrum short and funnel shaped. Ductus bursae tubular, membranous and long. Corpus bursae membranous and oval, signum leaf-shaped with tiny marginal spinules.

**Figure 44:**
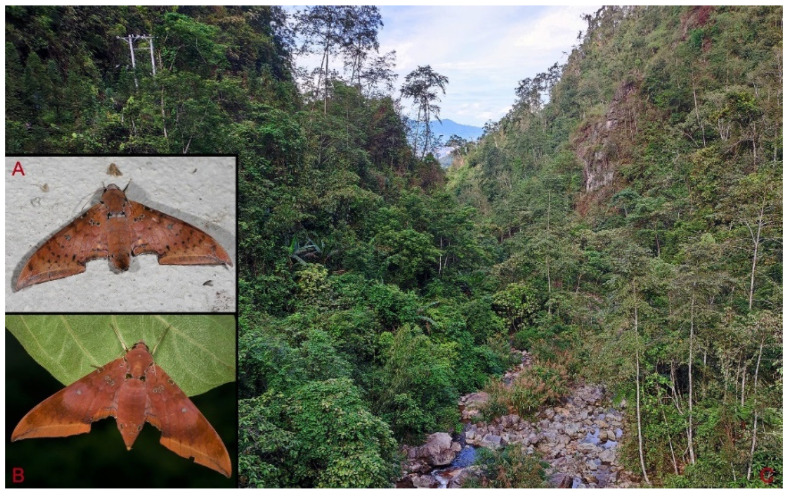
Habitat and living adults of *Ambulyx moorei*. (**A**) Male; (**B**) female; (**C**) Jinping, Yunnan, China.

**Biological notes:** This species was collected in low to mid-elevation monsoon evergreen broad-leaved forest and subtropical forests, attracted to light at night (Figure 44).

**Distribution:** China (Xizang, Yunnan, Guizhou, Guangdong, Hongkong, Guangxi, and Hainan); Sri Lanka, Nepal, Bhutan, India, Myanmar, Thailand, Vietnam, Laos, Philippines, Malaysia, and Indonesia.

#### 3.2.11. *Ambulyx ochracea* Butler, 1885 [裂斑鹰翅天蛾]

*Ambulyx ochracea* Butler, 1885; Cistula Entomologica, 3: 113; **Type locality:** Tochigi, Honshu, Japan.
  *Ambulyx ochracea kyora* Kishida, 2019; Japan Heterocerists’ Journal, 288: 312 [38].

**Diagnosis:** Male (Figure 46A,B): Body similar to *A. maculifera*. Forewing longer and patterns paler, upperside ground colour brownish-yellow, black-green subbasal spot generally more circular than in *A. s. sericeipennis* with a small notch at the dorso-distal corner, subterminal line brownish-grey, more weakly arched than *A. maculifera*, the greenish-yellow edge reaching the tornus, a blackish green spot in the submarginal area between veins CuA_1_ and CuA_2_ with a more indistinct margin than in *A. maculifera*. Hindwing upperside with a conspicuous black spot near the apex.

**Figure 45:**
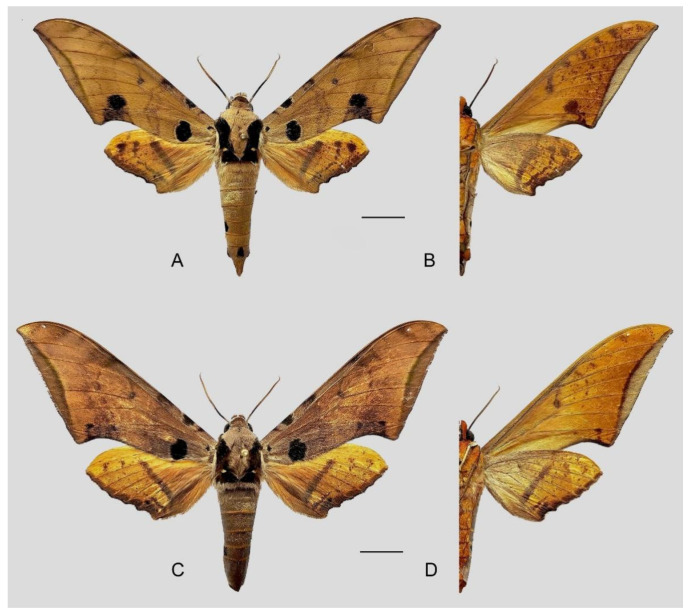
*Ambulyx ochracea*. (**A**,**B**) Male, Hangzhou, Zhejiang, China; (**C**,**D**) female, Yintiaoling Nature Reserve, Wuxi County, Chongqing, China. Scale bar = 10 mm.

Female (Figure 45C,D): Similar to male, but wings broader and forewing ground colour ochraceous, pattern darker, subterminal line fading before reaching the tornus, marginal area brown, antennae much slenderer.

**Figure 46:**
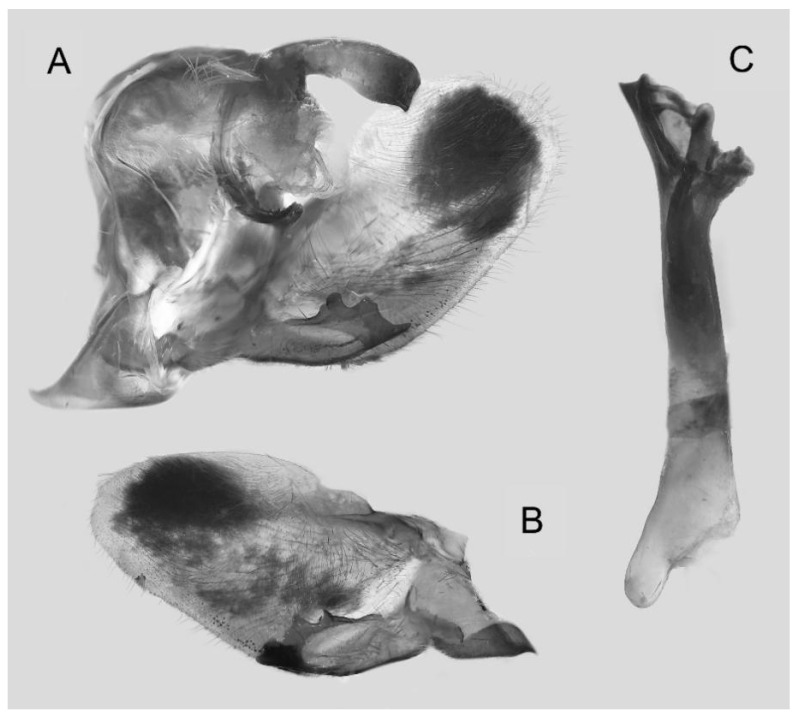
Male genitalia of *Ambulyx ochracea*, Mengla County, Yunnan, China. (**A**) Lateral view; (**B**) left valve; (**C**) phallus.

Male genitalia (Figure 46A–C): Uncus and gnathos similar to *A. maculifera*. Sacculus heavy, dorsal margin with two triangular lobes of varying shape, harpe broad, apex reduced to a short, blunt hook. Phallus much longer, posterior lobe sclerotized, and sharp apical, with row of teeth dorsally and some irregular teeth ventrally. Phallus shorter than *A. maculifera*, posterior lobe blunt with a tiny branch distally.

**Figure 47:**
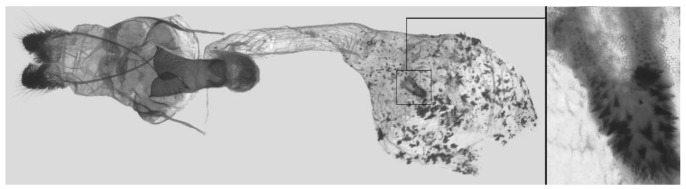
Female genitalia of *Ambulyx ochracea*, Yintiaoling Nature Reserve, Wuxi County, Chongqing, China.

Female genitalia (Figure 47): Similar to those of *A. maculifera*. Antrum funnel-shaped, anterior part leaf-shaped and broad. Ductus bursae long and membranous. Corpus bursae membranous and oval. Signum U-shaped and covered with tiny teeth.

**Figure 48:**
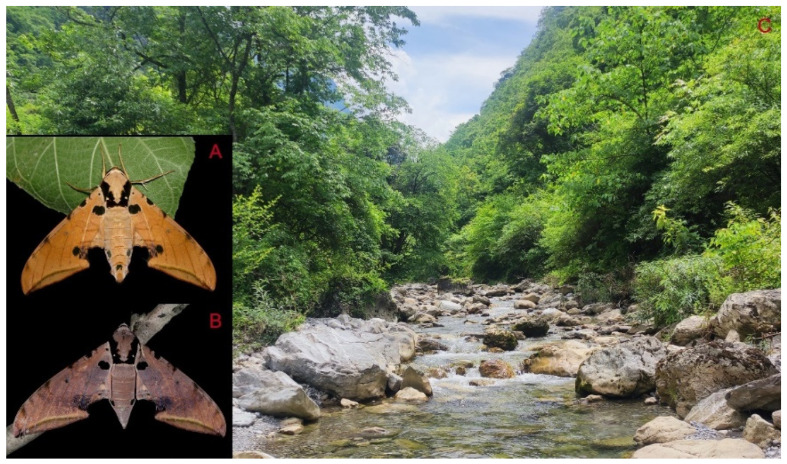
Habitat and living adults of *Ambulyx ochracea*. (**A**) Male; (**B**) female; (**C**) Wuxi County, Chongqing, China.

**Biological notes:** This species was collected in low to mid-elevation monsoon evergreen broad-leaved forest, attracted to light at night (Figure 48).

**Distribution:** China (Shaanxi, Hubei, Sichuan, Gansu, Henan, Anhui, Jiangsu, Zhejiang, Fujian, Tai-wan, Jiangxi, Hunan, Chongqing, Guangdong, Hongkong, Guangxi, Yunnan, Tibet, and Guizhou); South Korea, Japan, Nepal, Bhutan, India, Myanmar, Thailand, Laos, and Vietnam.

#### 3.2.12. *Ambulyx placida* Moore, 1888 [散斑鹰翅天蛾]

*Ambulyx placida* Moore, 1888; Proc. zool. Soc. Lond., 1888: 390; **Type locality:** Solon, Himachal Pradesh, India.

*Oxyambulyx citrona* Joicey & Kaye, 1917; Annals and Magazine of Natural History, (8) 20: 309 [39].

*Ambulyx placida nepalplacida* Inoue, 1992; Tyô to Ga, 43(3): 187 [18].

**Figure 49:**
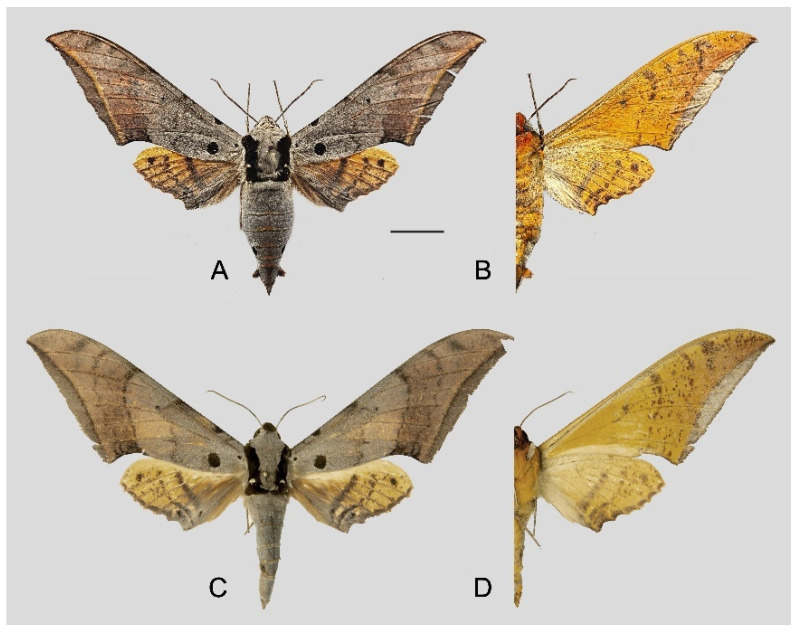
*Ambulyx placida*. (**A**,**B**) Male, Nyingchi, Xizang, China; (**C**,**D**) female, S. Xizang, China. © Sphingidae Museum. Scale bar = 10 mm.

**Diagnosis:** Male (Figure 49A,B): Similar to *A*. *japonica koreana* but much larger and generally much paler, with lateral black spots on each side of abdominal segment 6. Forewing longer, upperside ground colour grey, a black basal spot present, basal to medial area with a blackish-grey zigzag line, discal spot tiny and black, medial area to submarginal area with scattered with brown-greyish scales, a narrow brownish-black patch near the apex and the tornus, subterminal line brownish, arched and reaches the inner margin just basad to the tornus, its inner edge highlighted in yellow, marginal area dark grey; underside—ground colour yellow, covered with scattered black scales. Hindwing—upperside pale yellow covered with scattered black scales, a dark grey curved line in the medial area, apex with a black spot, tornus greyish-yellow; underside—almost the same as the upperside but colour and pattern paler.

Female (Figure 49C,D): Similar to male but wings broader and ground colour slightly darker, antennae much slenderer.

**Figure 50:**
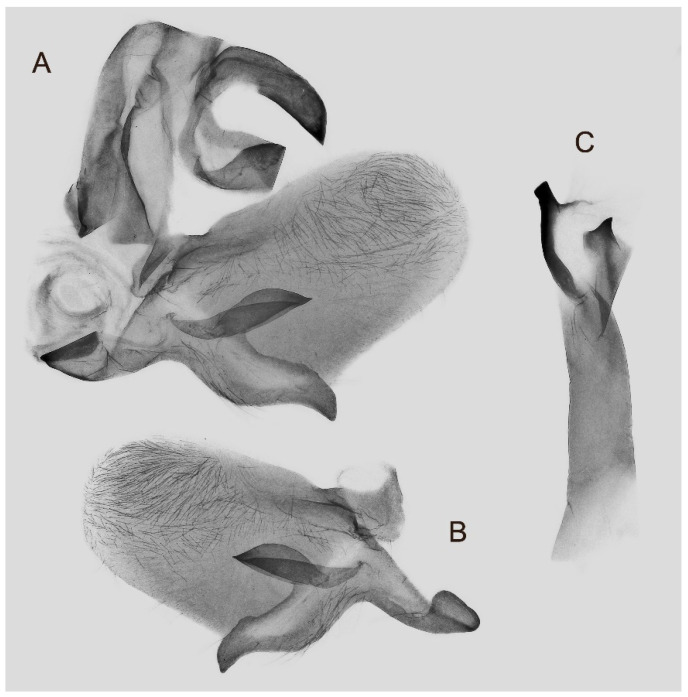
Male genitalia of *Ambulyx placida*, Nyingchi, Xizang, China. (**A**) Lateral view; (**B**) left valve; (**C**) phallus.

Male genitalia (Figure 50A–C): Similar to *A*. *j*. *japonica* but gnathos medially unnotched and patch of friction scales on valve tongue-shaped and broader. Dorsal lobe of sacculus and harpe slenderer and more separated. Phallus more curved, posterior lobe sharp and shorter, with a tiny terminal, rather than lateral tooth.

**Figure 51:**
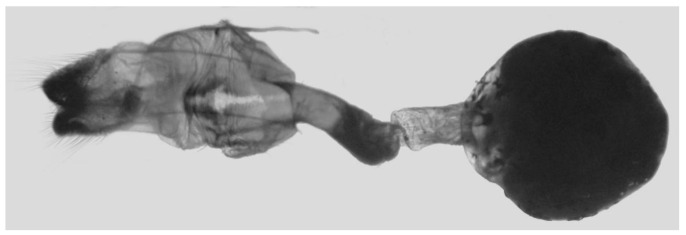
Female genitalia of *Ambulyx placida*, © The Trustees of the Natural History Museum, London, UK (downloaded from Kitching, I.J. Sphingidae Taxonomic Inventory. http://sphingidae.myspecies.info/, accessed on 20 April 2024.

Female genitalia (Figure 51): Similar to *A*. *japonica koreana* but antrum longer.

**Figure 52:**
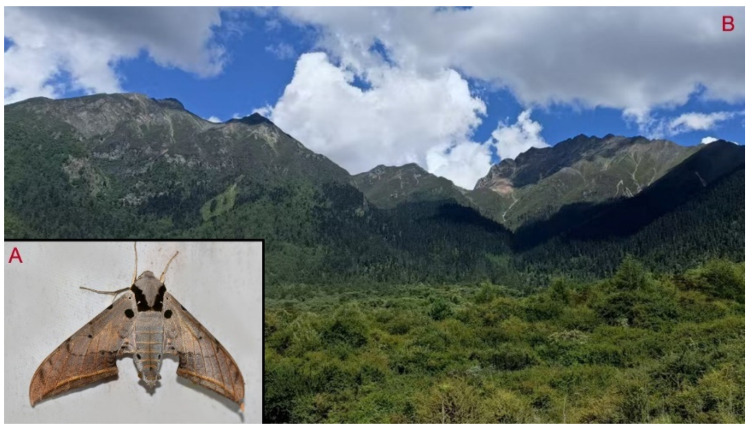
Habitat and living adult of *Ambulyx placida*. (**A**) Male; (**B**) Nyingchi, Xizang, China.

**Biological notes:** This species was collected in high elevation monsoon evergreen broad-leaved forest, attracted to light at night (Figure 52).

**Distribution:** China (S. and SE. Xizang); Nepal, Bhutan, India.

#### 3.2.13. *Ambulix schauffelbergeri* Bremer & Grey, 1853 [核桃鹰翅天蛾]

*Ambulix schauffelbergeri* Bremer & Grey, 1853; in Motschulsky (ed.), Etudes Entomologiques, 1: 62; **Type locality:** Pekin, China.

*Ambulyx trilineata* Rothschild, 1894; Novitates Zoologicae, 1: 88 [4].
*Oxyambulyx schauffelbergeri sobrina* (Mell, 1922); Beiträge zur Fauna sinica. Biologie und Systematik der südchinesischen Sphingiden, 21 [40].
*Oxyambulyx schauffelbergeri siaolouensis* Clark, 1937; Proceedings of the New England Zoological Club, 16: 28 [41].

**Figure 53:**
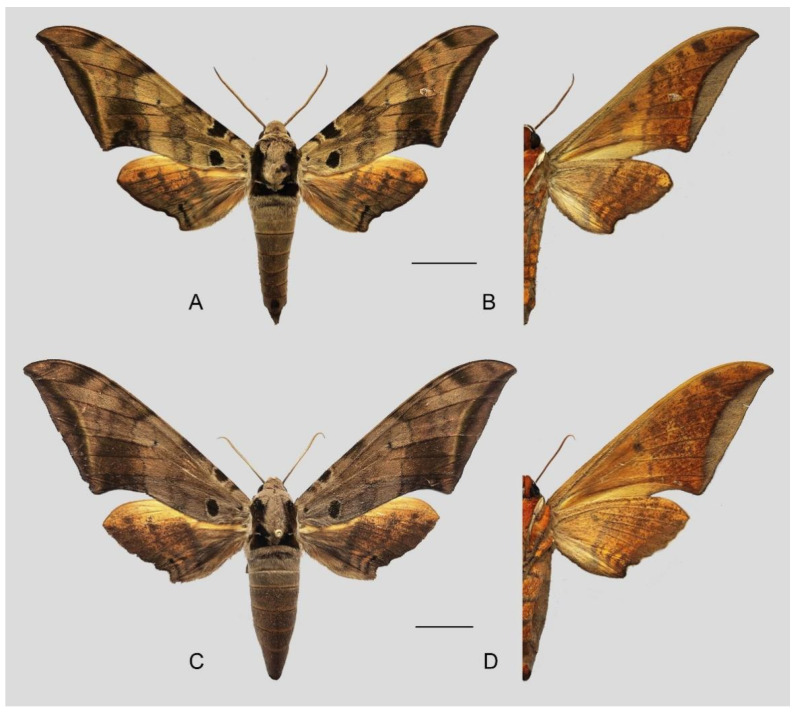
*Ambulyx schauffelbergeri*. (**A**,**B**) Male, Mt. Laoshan, Nanjing, Jiangsu, China; (**C**,**D**) female, Zhenping County, Shaanxi, China. Scale bar = 10 mm.

**Diagnosis:** Male (Figure 53A,B): Similar to *A. maculifera* but generally smaller and ground colour tending to be more brownish-yellow. Forewing narrower, upperside ground colour brownish-yellow, subbasal black-green spot generally smaller than in *A. maculifera*, basal to medial area with heavy black-brown zigzag lines and patches, subterminal line with many more scattered black scales, strongly arched and the yellow inner edge narrow, ending at the tornus, the blackish green spot in the submarginal area between veins CuA_1_ and CuA_2_ generally smaller and more indistinct than in *A. maculifera*, marginal area greyish-black; underside covered with dense scattered brownish-black scales, marginal area grey Hindwing upperside with heavy zigzag lines and with many more scattered greyish-black scales.

Female (Figure 53C,D): Similar to the male but wings broader, forewing upperside colour and pattern paler, subbasal black-green spot smaller, antennae much slenderer.

**Figure 54:**
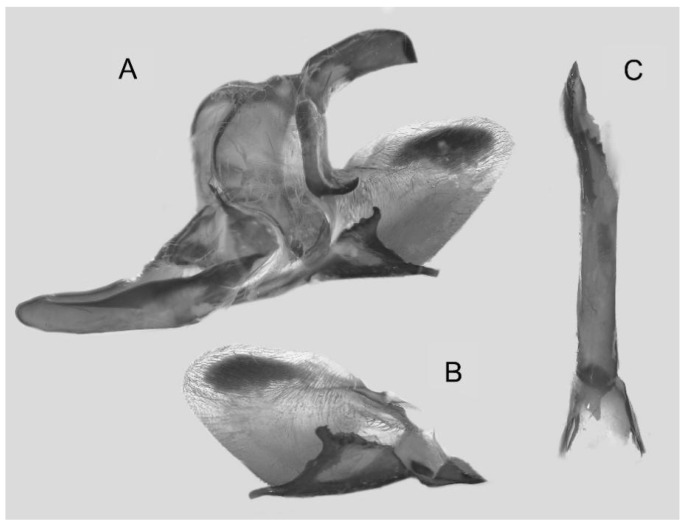
Male genitalia of *Ambulyx schauffelbergeri*, Menghai County, Yunnan, China. (**A**) Lateral view; (**B**) left valve; (**C**) phallus.

Male genitalia (Figure 54 A–C): Similar to *A. s. sericeipennis*. Uncus and gnathos heavy and thicker. Sacculus shorter and broader than in *A. s. sericeipennis*, dorsal ridge irregularly dentate with a variably shaped apical tooth, harpe narrowed to a finger-like lobe. Phallus slender, posterior lobe apically sharp, with tiny teeth ventrally.

**Figure 55:**
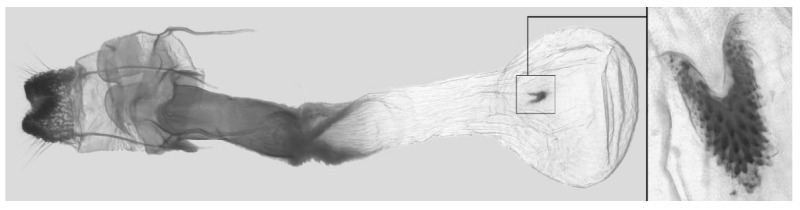
Female genitalia of *Ambulyx schauffelbergeri*, Yingshan County, Hubei, China.

Female genitalia (Figure 55): Similar to those of *A. s. sericeipennis*. Antrum cylindrical. Ductus bursae tubular and membranous. Signum tongue-shaped and narrower than in *A. s. sericeipennis*, with covered with denser spines.

**Figure 56:**
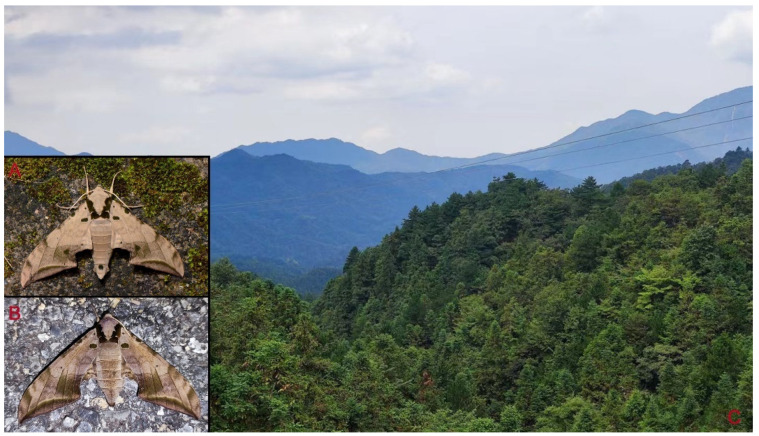
Habitat and living adults of *Ambulyx schauffelbergeri*. (**A**) Male; (**B**) female; (**C**) Yuexi County, Anhui, China.

**Biological notes:** This species was collected in low to mid-elevation monsoon evergreen broad-leaved forest, attracted to light at night (Figure 56).

**Distribution:** China (Liaoning, Hebei, Beijing, Shandong, Shaanxi, Henan, Jiangsu, Anhui, Shanghai, Zhejiang, Hubei, Sichuan, Chongqing, Yunnan, Xizang, Guizhou, Hunan, Fujian, Guangdong, and Hainan); South Korea, Japan, Nepal, India, Laos, and Vietnam.

#### 3.2.14. *Ambulyx semiplacida semiplacida* Inoue, 1990 [圆斑鹰翅天蛾指名亚种]

*Ambulyx semiplacida semiplacida* Inoue, 1990; Tinea, 12: 248; **Type locality:** Lushan Spa, Nantou Hsien, Taiwan.

**Figure 57:**
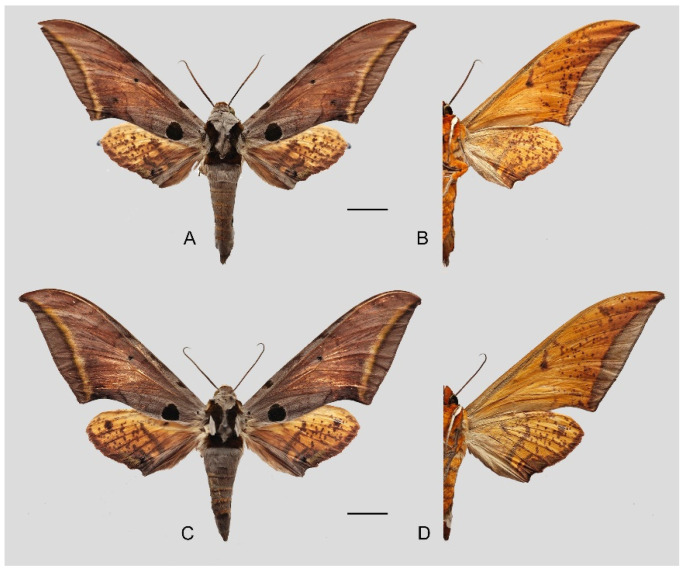
*Ambulyx semiplacida semiplacida*. (**A**,**B**) Male, Mt. Alishan, Taiwan, China; (**C**,**D**) female, Nantou County, Taiwan, China. Scale bar = 10 mm.

**Diagnosis:** Male (Figure 57A,B): Similar to *A. placida* but colour tends to be slightly bit darker. Forewing broader and wider, upperside ground colour purplish-grey, the subbasal black spot much larger than in *A. placida*, black discal spot conspicuous, subterminal line more strongly arched and the line of yellow scales along the inner edge much broader; underside—almost the same as the upperside but ground yellowish-orange, covered with dense scattered greyish scales. Hindwing—upperside pale yellow covered with dense scattered black scales; underside—almost the same as the upperside but ground colour in yellowish-orange and pattern paler.

Female (Figure 57C,D): Similar to male but wings broader and ground colour more purplish, pattern more intense, antennae much slenderer.

**Figure 58:**
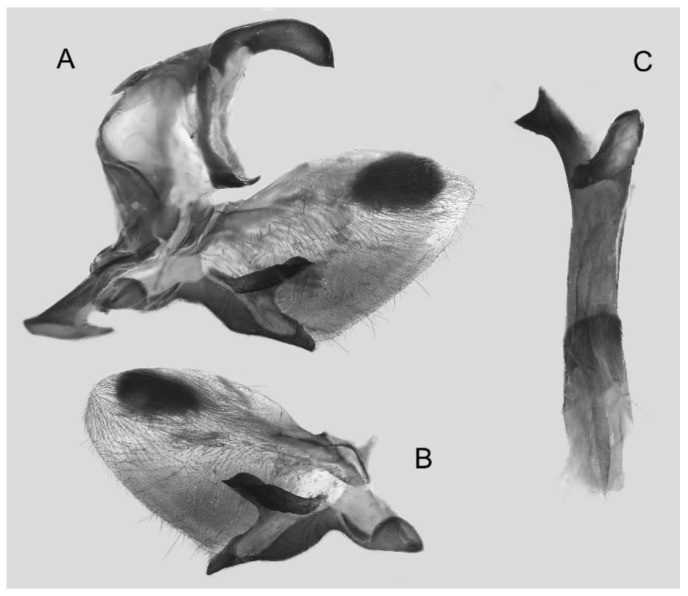
Male genitalia of *Ambulyx semiplacida semiplacida*, Mt. Alishan, Taiwan, China. (**A**) Lateral view; (**B**) left valve; (**C**) phallus.

Male genitalia (Figure 58A–C): Similar to *A. placida* but gnathos bilobed medially. Dorsal lobe of sacculus and harpe more strongly separated, each tending to be straighter than those in *A. placida*. Phallus posterior lobe sharp and longer, a large tooth laterally rather than terminally.

**Figure 59:**
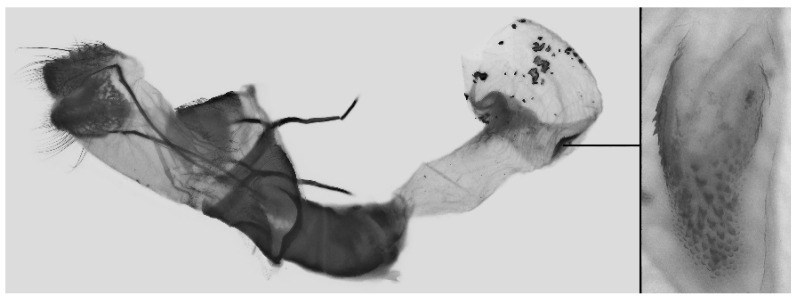
Female genitalia of *Ambulyx semiplacida semiplacida*, Nantou County, Taiwan, China.

Female genitalia (Figure 59): Similar to those of *A*. *placida*. Corpus bursae round, signum tongue-shaped with covered in sharp teeth.

**Figure 60:**
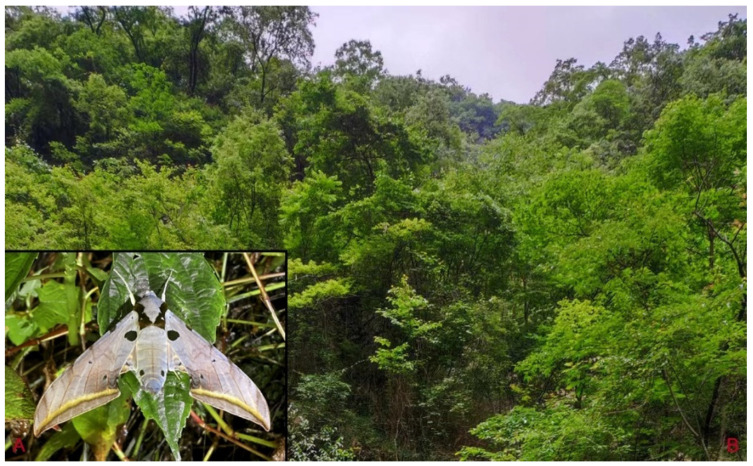
Habitat and living adult of *Ambulyx semiplacida semiplacida*. (**A**) Male; (**B**) Alishan, Taiwan, China.

**Biological notes:** This species was collected in high elevation monsoon evergreen broad-leaved forest, attracted to light at night or found resting on plants during the day (Figure 60).

**Distribution:** Endemic to the island of Taiwan.

#### 3.2.15. *Ambulyx semiplacida bhutana* Brechlin, 2014 [圆斑鹰翅天蛾不丹亚种]

*Ambulyx bhutana* Brechlin, 2014; Entomo-Satsphingia, 7(2): 50; **Type locality:** Trongsa Dzong, Bhutan.

**Figure 61:**
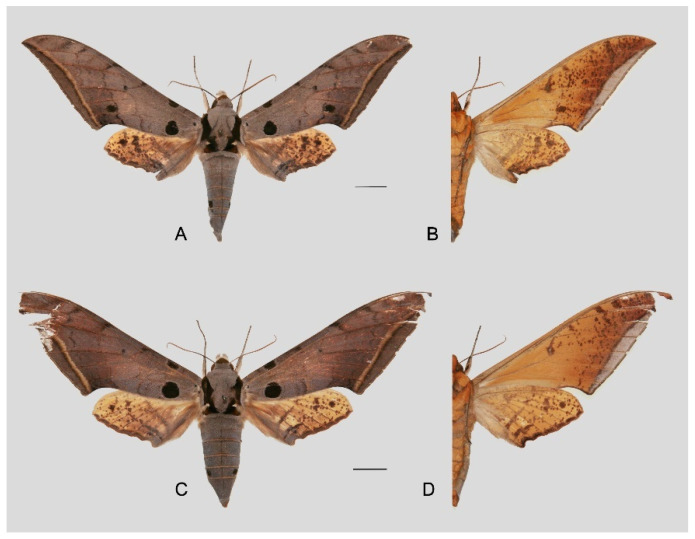
*Ambulyx semiplacida bhutana*. (**A**,**B**) HOLOTYPE, male, Trongsa Dzong, Bhutan; (**C**,**D**) ALLOTYPE, female, Jongkhar, Morong, Bhutan. Scale bar = 10 mm. © Ronald Brechlin.

**Diagnosis:** Male (Figure 61A,B): Similar to *A. s. semiplacida* but colour tending to be more greyish and pattern more uniform. Forewing narrower and longer, upperside black subbasal spot variable in size, subterminal line more weakly arched than in *A. s. semiplacida*; underside almost the same as the upperside but ground colour yellow, covered with dense scattered greyish-black scales. Forewing underside and hindwing upperside ground colour yellowish.

Female (Figure 61C,D): Similar to male but wings broader and ground colour darker, pattern more uniform, antennae much slenderer.

**Figure 62:**
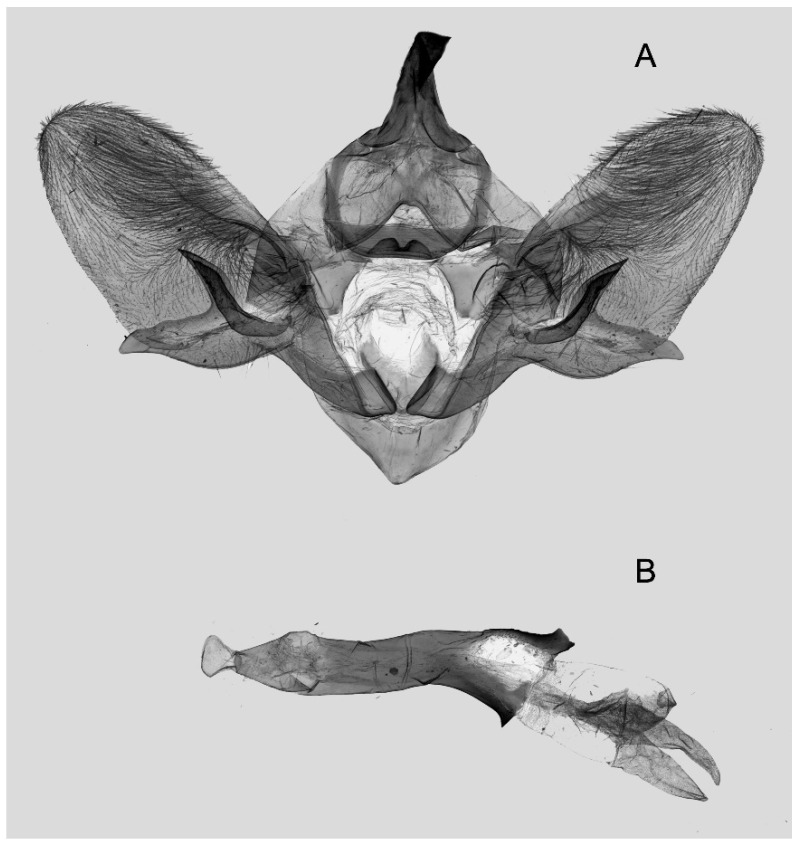
**PARATYPE**, Male genitalia of *Ambulyx semiplacida bhutana*, Trongsa, Bhutan. (**A**) Genital capsule; (**B**) phallus. © Ronald Brechlin.

Male genitalia (Figure 62A,B): Very similar to *A. s. semiplacida* but gnathos strongly bilobed.

Female genitalia: Not examined.

**Distribution:** China (S. Xizang); Bhutan, India.

#### 3.2.16. *Ambulyx semiplacida interplacida* Brechlin, 2006 [圆斑鹰翅天蛾大陆亚种]

*Ambulyx interplacida* Brechlin, 2006; Nachr. entomol. Ver. Apollo, N.F., 27(3): 103; **Type locality:** Xipaihe village, Wuyi Shan, Jiangxi, China.
  *Ambulyx amara* Kobayashi, Wang & Yano, 2006; Tinea, 19: 169 [42].
  *Ambulyx regia* Eitschberger, 2006; Atalanta, 37: 484 [32].
  *Ambulyx pseudoregia* Eitschberger & Bergmann, 2006; Atalanta, 37: 485 [25].

**Figure 63:**
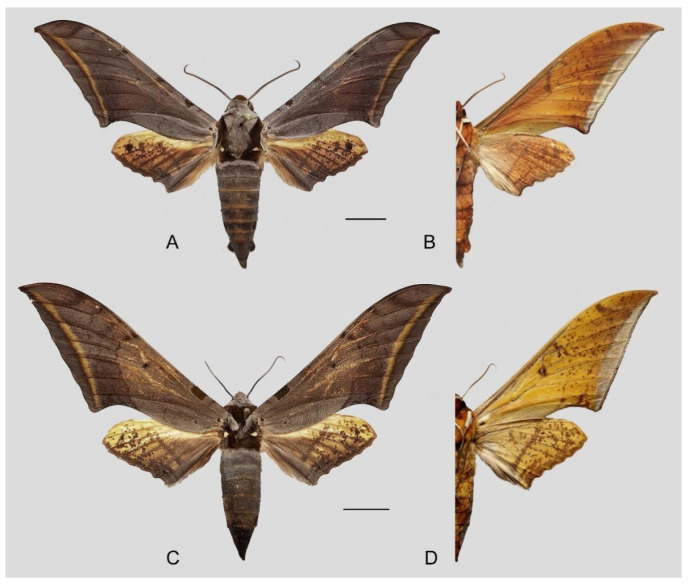
*Ambulyx semiplacida interplacida*. (**A**,**B**) Male, Shaoguan, Guangdong, China; (**C**,**D**) female, Fengtongzhai, Baoxing County, Sichuan, China. Scale bar = 10 mm.

**Diagnosis:** Male (Figure 63A,B): Similar to *A. s. semiplacida* but generally smaller, ground colour tending to be more purplish and pattern more uniform. Forewing upperside subbasal black spot absent. Hindwing upperside with dense scattered brownish-black scales. Underside of forewing and hindwing yellowish-orange.

Female (Figure 63C,D): Similar to male, but wings broader and ground colour darker, antennae much slenderer.

**Figure 64:**
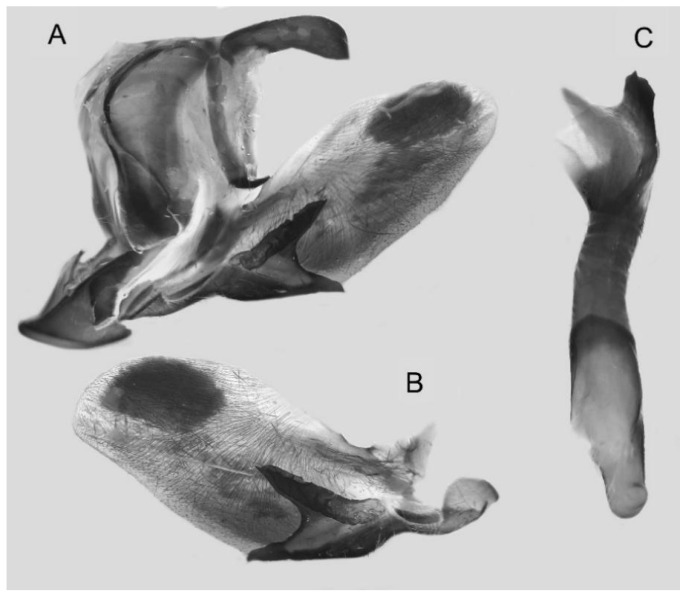
Male genitalia of *Ambulyx semiplacida interplacida*, Mt. Leigongshan, Suiyang County, Guizhou, China. (**A**) Lateral view; (**B**) left valve; (**C**) phallus.

Male genitalia (Figure 64A–C): Very similar to *A. s. semiplacida* but gnathos weakly bilobed and the dorsal lobe of the sacculus and the harpe shorter and thicker.

**Figure 65:**
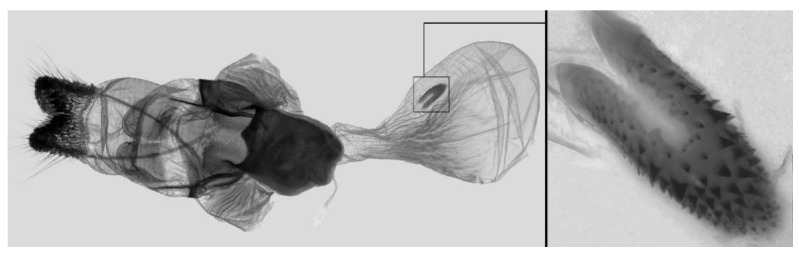
Female genitalia of *Ambulyx semiplacida interplacida*, Fengtongzhai, Baoxing County, Sichuan, China.

Female genitalia (Figure 65): Similar to those of *A*. *placida* but antrum funnel-shaped, much short and broader. Corpus bursae round, signum long tongue-shaped and covered with dense teeth.

**Figure 66:**
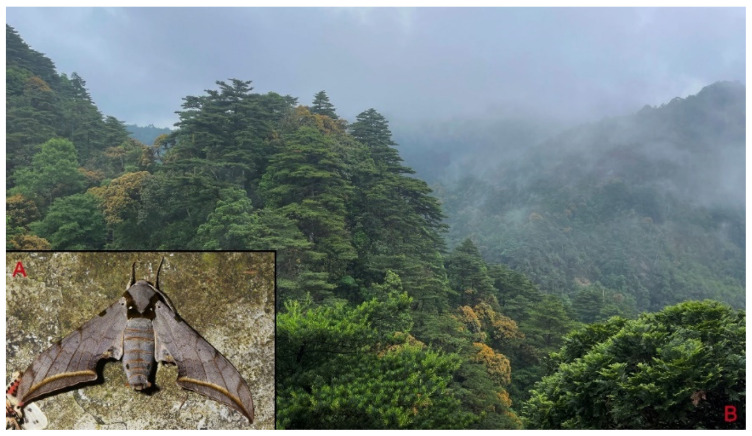
Habitat and living adult of *Ambulyx semiplacida interplacida*. (**A**) Male; (**B**) Nanling, Guangdong, China.

**Biological notes:** This subspecies was collected in high elevation monsoon evergreen broad-leaved forest, attracted to light at night (Figure 66).

**Distribution:** Currently known only from China (Yunnan, Sichuan, Guizhou, Jiangxi, Hunan, Guangdong, and Guangxi).

#### 3.2.17. *Ambulyx semiplacida* montana Cadiou & Kitching, 1990 [圆斑鹰翅天蛾缅泰亚种]

*Ambulyx montana* Cadiou & Kitching, 1990; Lambillionea, 90(4): 14; **Type locality:** Doi Inthanon National Park, Chiang Mai, Thailand.

**Figure 67:**
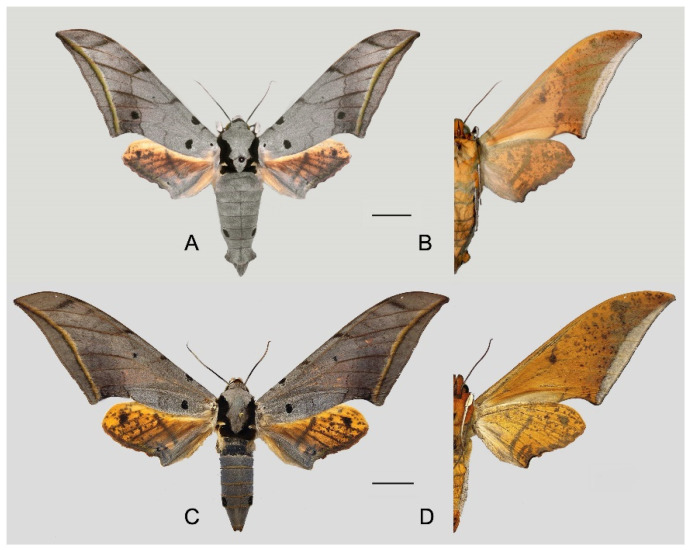
*Ambulyx semiplacida montana*. (**A**,**B**) Male, Pingbian, Yunnan, China; (**C**,**D**) female, Pingbian, Yunnan, China. Scale bar = 10 mm.

**Diagnosis:** Male (Figure 67A,B): Similar to *A*. *s. bhutana* but colour paler grey and patterned more uniform. Subbasal black spot on forewing upperside variable in size, smaller on average than in *A. s. semiplacida*. Forewing and hindwing upperside patterns paler and cleaner than in *A*. *s. bhutana*. Forewing and hindwing underside ground colour pale yellow.

Female (Figure 67C,D): Similar to male, but wings broader and ground colour darker, antennae much slenderer.

**Figure 68:**
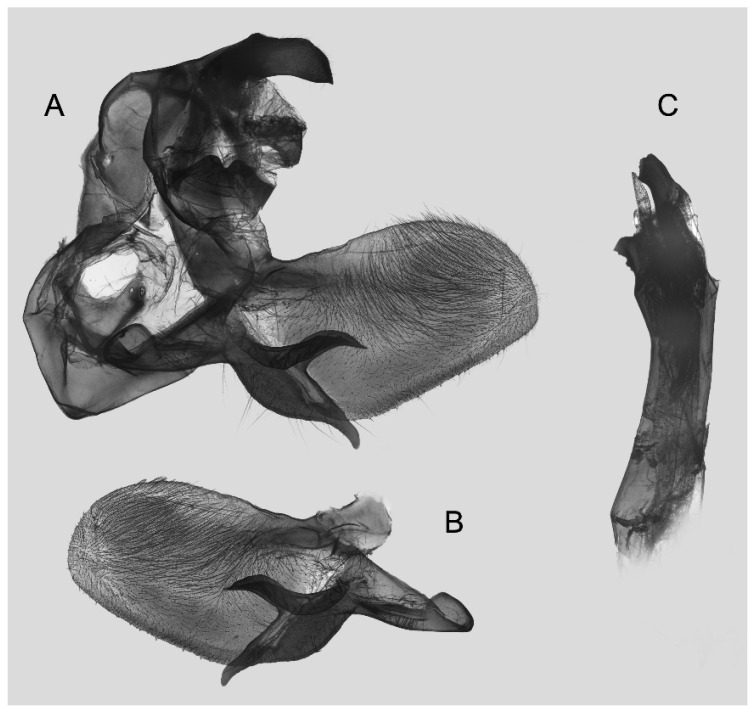
Male genitalia of *Ambulyx semiplacida montana*, Pingbian, Yunnan, China. (**A**) Lateral view; (**B**) left valve; (**C**) phallus.

Male genitalia (Figure 68A–C): Very similar to *A*. *s. bhutana* but gnathos weakly bilobed, dorsal lobe of sacculus and harpe longer and slenderer.

**Figure 69:**
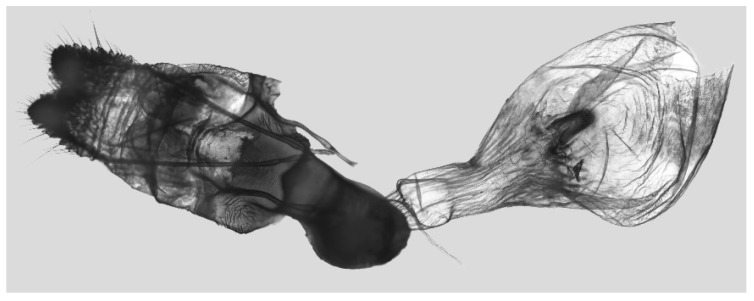
Female genitalia of *Ambulyx semiplacida montana*, Pingbian, Yunnan, China.

Female genitalia (Figure 69): Similar to those of *A*. *semiplacida interplacida* but antrum broader. Signum wider and apically blunt.

**Figure 70:**
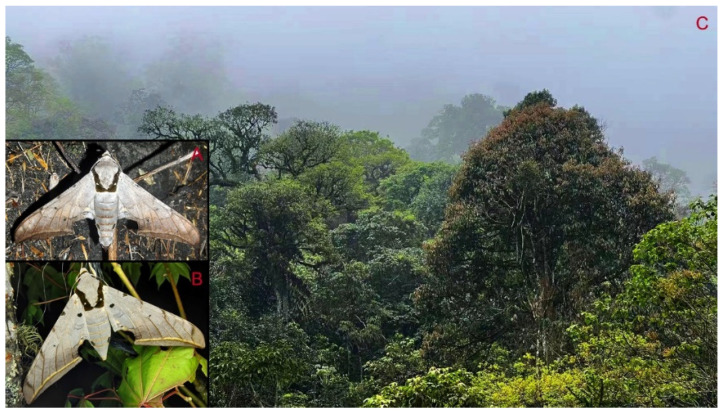
Habitat and living adults of *Ambulyx semiplacida montana*. (**A**) Male; (**B**) female; (**C**) Pingbian County, Yunnan, China.

**Biological notes:** This subspecies was collected in high elevation monsoon evergreen broad-leaved forest, attracted to light at night (Figure 70).

**Distribution:** China (SE Yunnan); Laos, Vietnam, Thailand, Myanmar, and India.

#### 3.2.18. *Ambulyx sericeipennis sericeipennis* Butler, 1875 [亚洲鹰翅天蛾指名亚种]

*Ambulyx sericeipennis* Butler, 1875; Proc. zool. Soc. Lond., 1875: 252; **Type locality:** Masuri, Uttarakhand, India.
  *Oxyambulyx sericeipennis brunnea* (Mell, 1922); Beiträge zur Fauna sinica. Biologie und Systematik der südchinesischen Sphingiden, 85 [40].
  *Oxyambulyx sericeipennis* reducta (Mell, 1922); Beiträge zur Fauna sinica. Biologie und Systematik der südchinesischen Sphingiden, 86 [40].
  *Oxyambulyx sericeipennis agana* Jordan, 1929; Novitates Zoologicae, 35: 85 [43].
  *Oxyambulyx okurai* Okano, 1959; Report of the Gakugei Faculty of Iwate University, 14: 40 [44].
  *Oxyambulyx amaculata* Meng, 1989; Entomotaxonomia, 11: 299 [45].

**Figure 71:**
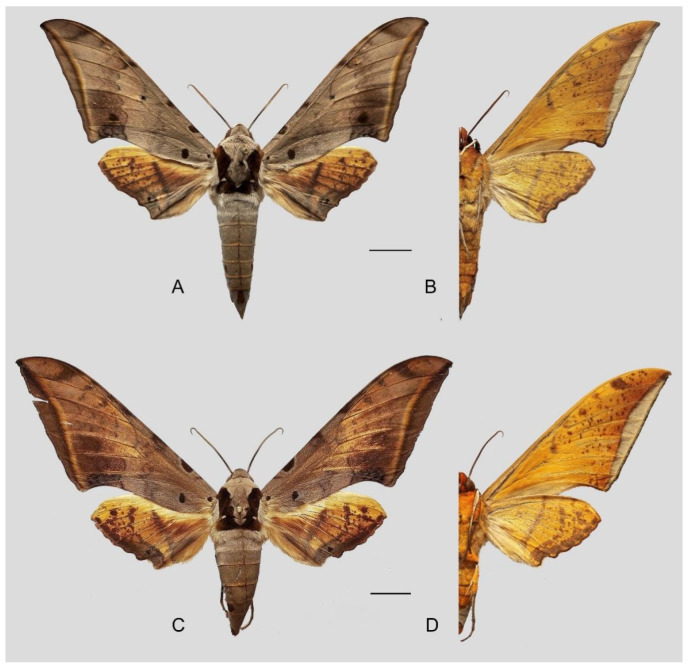
*Ambulyx sericeipennis sericeipennis*. (**A**,**B**) Male, Maolan Nature Reserve, Libo, Guizhou, China. (**C**,**D**) Female, Mt. Alishan, Taiwan, China. Scale bar = 10 mm.

**Diagnosis:** Male (Figure 71A,B): Similar to *A. placida* but coloration tends to be more brownish-yellow, a blackish green lateral spot present on the each side of abdominal segment 6, abdominal tergum A8 with a dark dorsal patch. Forewing shorter than *A. placida*, upperside ground colour yellowish-grey with scattered with brown scales, zigzag lines darker, subterminal line more strongly arched and the line of yellow scales along the inner edge much broader and reaching the tornus, a black spot in the submarginal area between veins CuA_1_ and CuA_2_; underside—almost the same as upperside but ground yellowish-orange, covered with dense scattered greyish scales. Hindwing upperside yellow, covered with dense scattered black scales.

Female (Figure 71C,D): Similar to the male, but wings broader and ground colour and pattern darker, antennae much slenderer.

**Figure 72:**
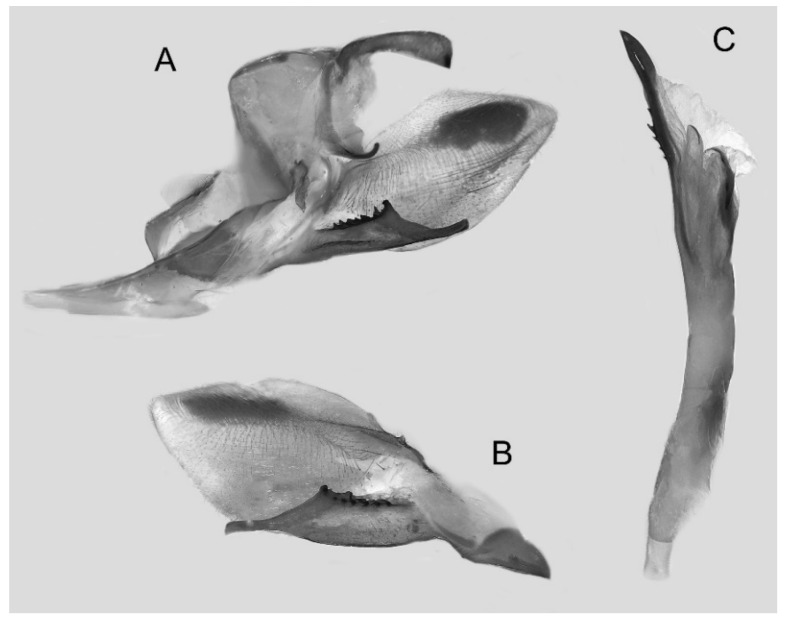
Male genitalia of *Ambulyx sericeipennis sericeipennis*, Mengla County, Yunnan, China. (**A**) Lateral view; (**B**) left valve; (**C**) phallus.

Male genitalia (Figure 72A–C): Similar to *A. placida* but gnathos with a rather narrow and almost truncate mesal lobe. Sacculus longer, dorsal margin irregularly dentate, harpe a long finger-like process, with a slight apical hook. Phallus much longer than *A. placida*, posterior lobe sharp apically, with row of teeth dorsally and some irregular teeth ventrally.

**Figure 73:**
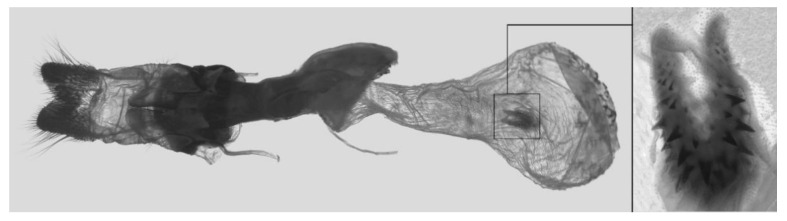
Female genitalia of *Ambulyx sericeipennis sericeipennis*, Gongshan County, Yunnan, China.

Female genitalia (Figure 73): Similar to those of *A. placida*. Antrum annular with a median finger-like process ventrally. Signum tongue-shaped with dense spines and covered in teeth.

**Figure 74:**
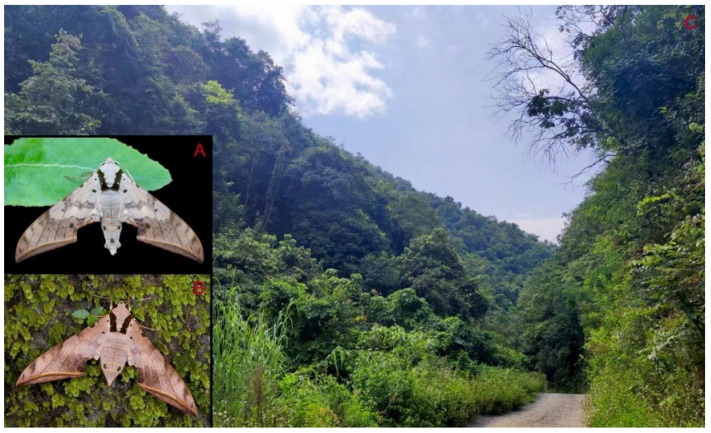
Habitat and living adults of *Ambulyx sericeipennis sericeipennis*. (**A**) Male; (**B**) female; (**C**) Yuanjiang, Yunnan, China.

**Biological notes:** This species was collected in mid- to high elevation monsoon evergreen broad-leaved forest, attracted to light at night or found resting on plants during the day (Figure 74).

**Distribution:** China (Shaanxi, Hubei, Sichuan, Gansu, Henan, Anhui, Jiangsu, Zhejiang, Fujian, Taiwan, Jiangxi, Hunan, Chongqing, Guangdong, Guangxi, Yunnan, Xizang, and Guizhou); Pakistan, Nepal, Bhutan, India, Myanmar, Laos, Thailand, Cambodia, and Vietnam.

#### 3.2.19. *Ambulyx sericeipennis* joiceyi (Clark, 1923) [亚洲鹰翅天蛾乔氏亚种]

*Oxyambulyx sericeipennis joiceyi* Clark, 1923; Proceedings of the New England Zoological Club, 8: 70 [46]; **Type locality:** Mt. Korintji, Sumatra, Indonesia.

**Figure 75:**
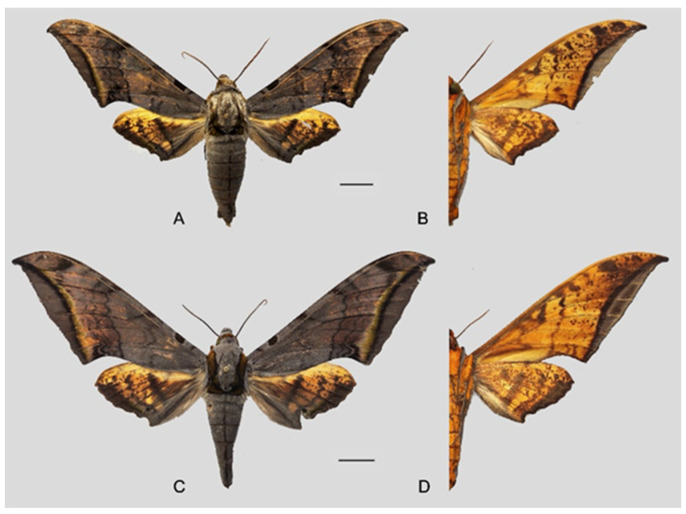
*Ambulyx sericeipennis joiceyi*. (**A**,**B**) Male, Sabah, Borneo, Malaysia. (**C**,**D**) Female, Sumatra, Indonesia. Scale bar = 10 mm.

**Diagnosis:** Male (Figure 75A,B): Similar to *A. s. sericeipennis* but upperside body colour greyish, V-shaped patch on thorax dorsally yellow-greenish. Forewing narrower and longer than *A. s. sericeipennis*, upperside ground colour purplish-grey, with heavy and darker zigzag lines in the medial area, subbasal black spot absent, subterminal line more strongly arched and the line of yellowish-green scales along its inner edge much broader, a black dentate patch near the tornus; underside ground yellow, covered with dense scattered black scales and zigzag stripes. Hindwing upperside pattern darker than *A. s. sericeipennis*, marginal area greyish-black.

Female (Figure 75C,D): Similar to male, but wings broader and ground colour and pattern darker, line of yellowish-green scales along the inner edge of the subterminal line on the forewing upperside even broader, antennae much slenderer.

**Figure 76:**
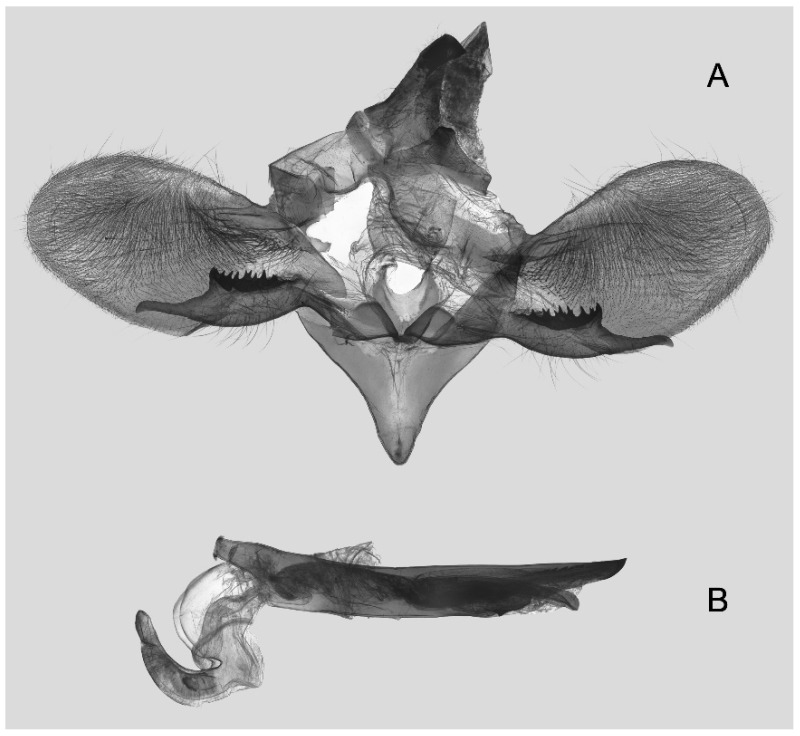
Male genitalia of *Ambulyx sericeipennis joiceyi*, Sabah, Borneo, Malaysia. (**A**) Genital capsule; (**B**) phallus.

Male genitalia (Figure 76A,B): Similar to *A. s. sericeipennis* but valves more rounded and shorter. Harpe apex process slender and more downcurved apically. Phallus posterior lobe apically shorter and thicker than in *A. s. sericeipennis*, with more irregular teeth dorsally.

**Figure 77:**
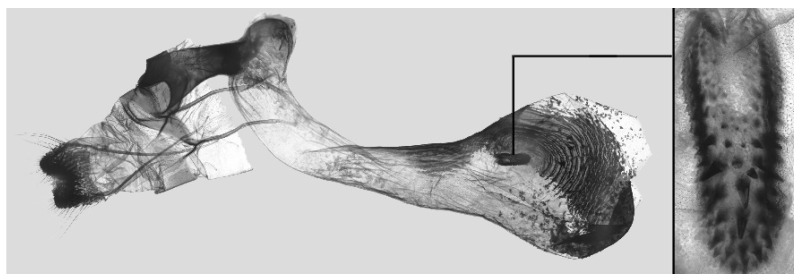
Female genitalia of *Ambulyx sericeipennis joiceyi*, Sumatra, Indonesia.

Female genitalia (Figure 77): Similar to those of *A. s. sericeipennis*. Ductus bursae tubular, membranous and longer. Signum tongue-shaped, much slenderer than in *A. s. sericeipennis* and covered with thicker spines and teeth.

**Figure 78:**
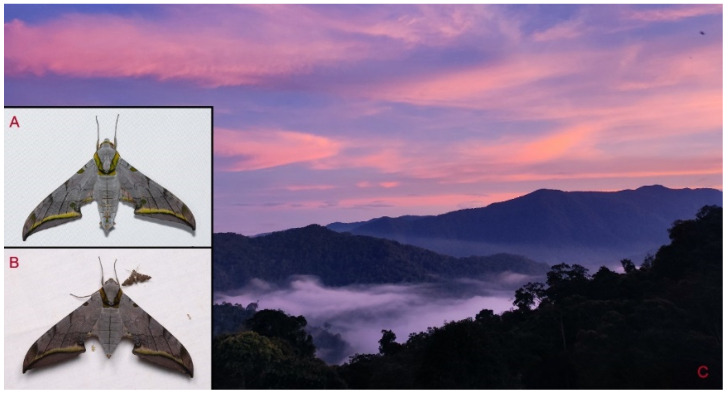
Habitat and living adults of *Ambulyx sericeipennis joiceyi*. (**A**) Male; (**B**) female; (**C**) Sabah, Borneo, Malaysia.

**Biological notes:** This subspecies was collected in mid-elevation subtropical forests, attracted to light at night (Figure 78).

**Distribution:** Malaysia (Peninsular Malaysia and Sabah, Borneo), Indonesia (Kalimantan, Borneo, and Sumatra).

#### 3.2.20. *Ambulyx sericeipennis javanica* (Clark, 1930) [亚洲鹰翅天蛾爪哇亚种]

*Oxyambulyx sericeipennis javanica* Clark, 1930; Proceedings of the New England Zoological Club, 12: 26 [47]; **Type locality:** Mt. Gede, Java, Indonesia.

**Figure 79:**
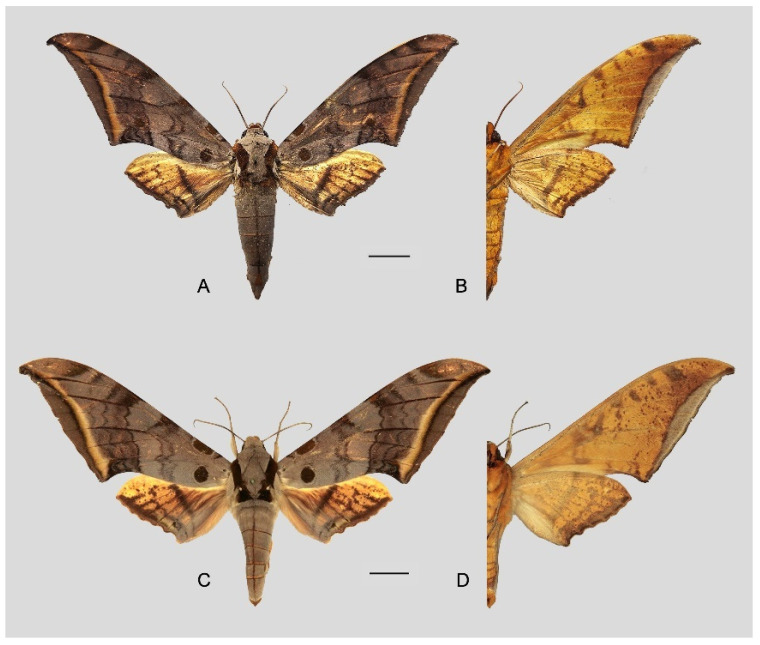
*Ambulyx sericeipennis javanica*. (**A**,**B**) Male, Mt. Halimun, Java, Indonesia. (**C**,**D**) Female, Mt. Mt. Halimun, Java, Indonesia. Scale bar = 10 mm. © The Trustees of the Natural History Museum, London, UK (downloaded from Kitching, I.J. Sphingidae Taxonomic Inventory. http://sphingidae.myspecies.info/, accessed on 20 April 2024.

**Diagnosis:** Male (Figure 79A,B): Similar to *A. s. joiceyi* but smaller and body colour tending to be slightly yellow. Forewing ground colour paler, with darker zigzag lines on medial area, a subbasal black spot present, subterminal line arched and line of yellowish-green scales along its inner edge broader. Hindwing upperside pattern paler and sparser than in *A. s. joiceyi*, marginal area brownish-black.

Female (Figure 79C,D): Similar to male but wings broader and ground colour and pattern darker, line of yellowish-green scales along the inner edge of the subterminal line on the forewing upperside broader, antennae much slenderer.

**Figure 80:**
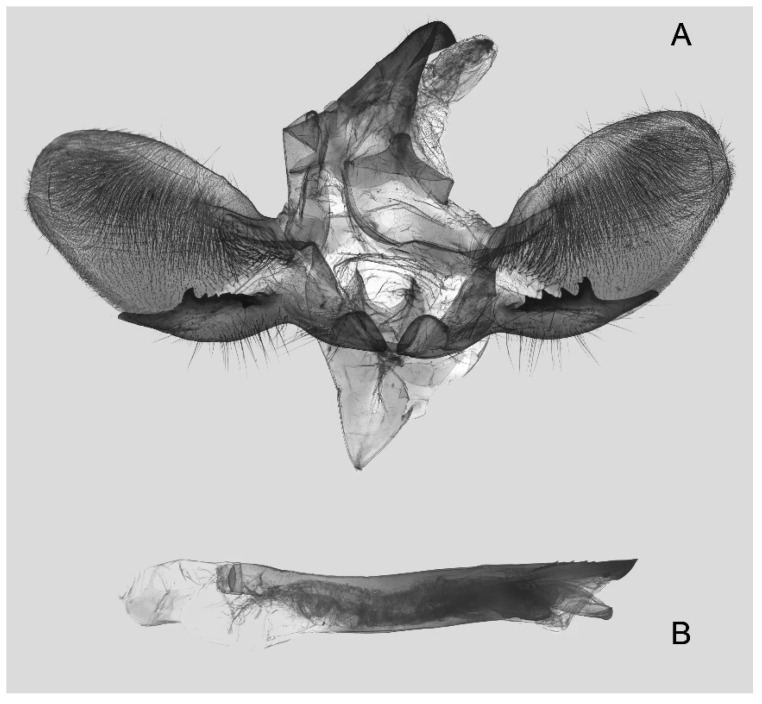
Male genitalia of *Ambulyx sericeipennis javanica*, Mt. Halimun, Java, Indonesia. (**A**) Genital capsule; (**B**) phallus.

Male genitalia (Figure 80A,B): Similar to *A. s. joiceyi* but dorsal margin of sacculus with a smaller number of larger teeth and harpe shorter than in *A. s. joiceyi*.

Female genitalia: Not examined.

**Figure 81:**
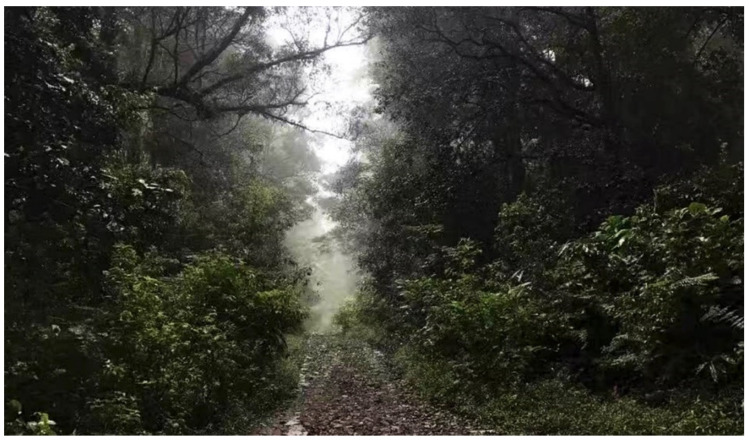
Habitat of *Ambulyx sericeipennis javanica*, Mt. Tangkuban Perabu, Java, Indonesia.

**Biological notes:** This subspecies was collected in mid-elevation subtropical forests (Figure 81), attracted to light at night.

**Distribution:** Indonesia (Java).

#### 3.2.21. *Ambulyx sericeipennis luzoni* (Clark, 1924) [亚洲鹰翅天蛾吕宋亚种]

*Oxyambulyx sericeipennis luzoni* Clark, 1924; Proceedings of the New England Zoological Club, 9: 14 [48]; **Type locality:** Baguio, Luzon, Philippines.

**Figure 82:**
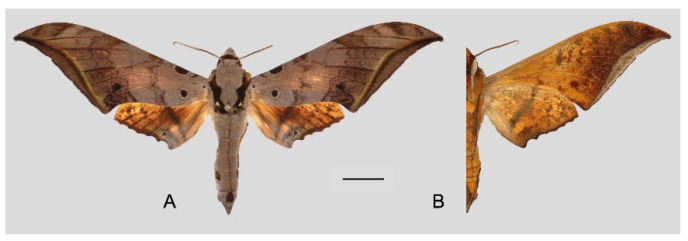
*Ambulyx sericeipennis luzoni*. (**A**,**B**) Male, Mt. Polis, Luzon, Philippines. Scale bar = 10 mm. © The Trustees of the Natural History Museum, London, UK.

**Diagnosis:** Male (Figure 82A,B): Similar to *A. s. javanica* smaller and body colour tending to be slightly ochre. Forewing upperside ground pattern paler, the subbasal black spot much smaller, subterminal line strongly arched and the line of yellowish-green scales along its inner edge narrower than *A. s. javanica*; underside ground colour ochre-yellow, covered with dense scattered black scales. Hindwing upperside ground colour yellowish-ochre, medial area with a broader black-greyish band and an ochre patch.

Female: Similar to male but wings broader and ground colour and pattern darker, antennae much slenderer.

**Figure 83:**
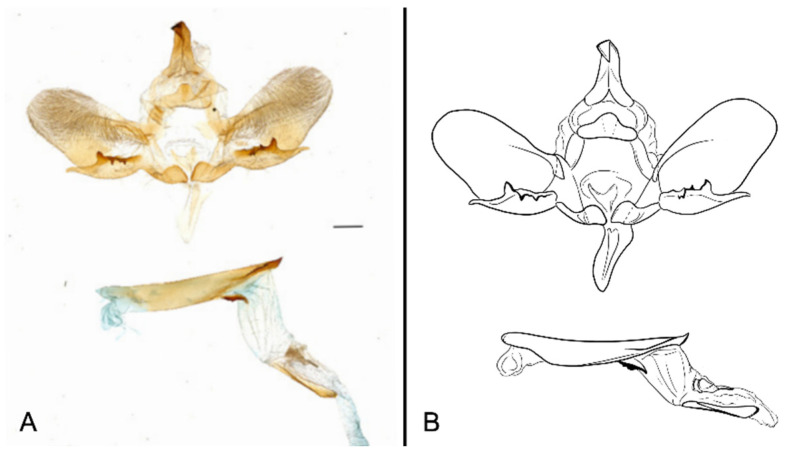
Male genitalia of *Ambulyx sericeipennis luzoni*, Luzon, Philippines. (**A**) Original genitalia slide photos [16]; (**B**) line drawing by the first author.

Male genitalia (Figure 83A,B): Similar to *A. s. javanica* but dorsal margin of sacculus with a smaller and more irregular teeth and harpe shorter and more curved than in *A. s. javanica*.

Female genitalia: Not examined.

**Distribution:** The Philippines (Luzon).

#### 3.2.22. *Ambulyx sericeipennis palawanica* Brechlin, 2009 [亚洲鹰翅天蛾巴拉望亚种]

*Ambulyx sericeipennis palawanica* Brechlin, 2009; Entomo-Satsphingia, 2: 60 [16]; **Type locality:** Mt. Mantalingajan, Palawan, Philippines.

**Figure 84:**
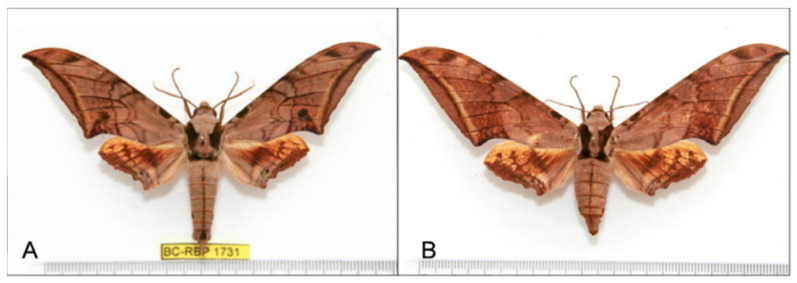
*Ambulyx sericeipennis palawanica*. (**A**) HOLOTYPE, male, Palawan, Philippines. (**B**) PARATYPE, female, Palawan, Philippines. Scale bar = 10 mm. [16].

**Figure 85:**
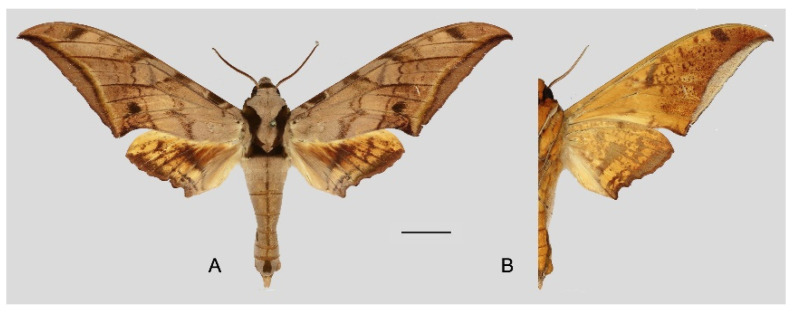
*Ambulyx sericeipennis palawanica*. Male, Mt. Brooks, Palawan, Philippines. (**A**) upperside; (**B**) underside. Scale bar = 10 mm. © The Trustees of the Natural History Museum, London, UK.

**Diagnosis:** Male (Figure 84A and Figure 85A): Similar to *A. s. luzoni* but body colour tending to be paler. Forewing upperside ground colour greyish-yellow, pattern and black scales on the veins heavier, the subbasal black spot absent, subterminal line not as arched as in *A. s. luzoni* and the line of yellowish-green scales along its inner edge broader than in *A. s. luzoni*; underside colour yellow covered with dense scattered greyish scales. Hindwing upperside ground colour yellow, medial area with broader black dentate patch rather than the band seen in *A. s. luzoni*.

Female (Figure 84B): Similar to male, but wings broader and ground colour and pattern paler, antennae much slenderer.

**Figure 86:**
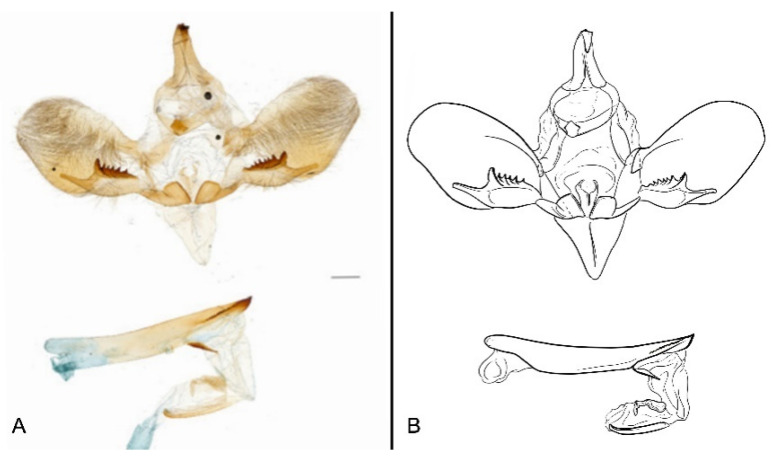
PARATYPE, male genitalia of *Ambulyx sericeipennis palawanica*, Palawan, Philippines. (**A**) Original genitalia slide photo [16]; (**B**) line drawing from the photo by the first author.

Male genitalia (Figure 86A,B): Similar to *A. s. luzoni* but teeth along the dorsal edge of the sacculus more uniform in size and denser in *A. s. luzoni*.

Female genitalia: Not examined.

**Distribution:** The Philippines (Palawan).

#### 3.2.23. *Ambulyx siamensis* Inoue, 1991 [暹罗鹰翅天蛾]

*Ambulyx siamensis* Inoue, 1991; Tinea, 13(14): 130; **Type locality:** Nam Proam, Chaiyaphum, Thailand.

**Figure 87:**
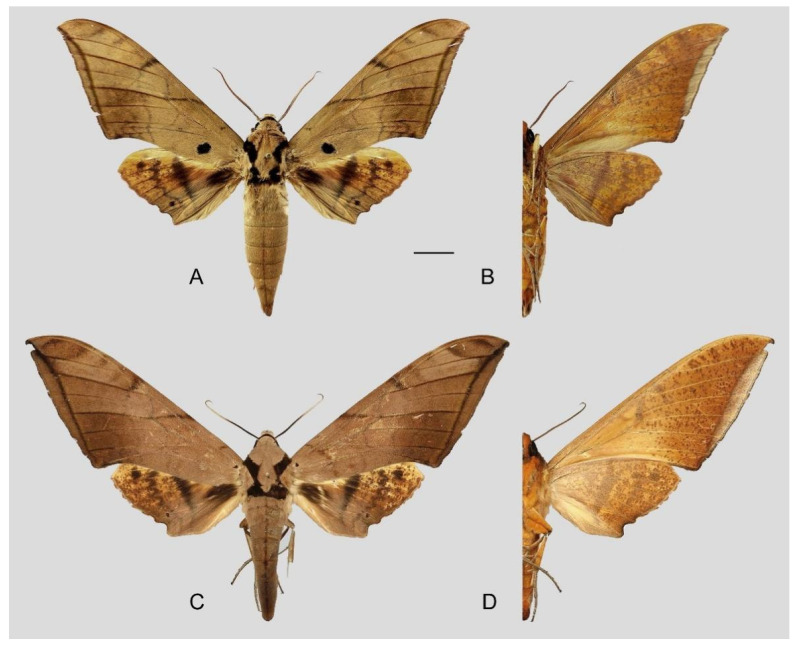
*Ambulyx siamensis*. (**A**,**B**) Male, Nabanhe Nature Reserve (1093 m), Xishuangbanna, Yunnan, China. (**C**,**D**) Female, Nan, Thailand. © The Trustees of the Natural History Museum, London, UK (downloaded from Kitching, I.J. Sphingidae Taxonomic Inventory. http://sphingidae.myspecies.info/, accessed on 20 April 2024.

**Diagnosis:** Male (Figure 87A,B): Similar to *A. substrigilis* but forewing upperside pattern paler and more indistinct, subbasal spot much smaller than in *A. substrigilis* or even absent, subterminal line narrow in blackish, slightly arched and fading just before reaching the tornus, no spot in the submarginal area between veins CuA_1_ and CuA_2_; underside subterminal line indistinct, brownish. Hindwing patterns heavier than in *A. substrigilis* with more scattered black scales.

Female (Figure 87C,D): Similar to male, but wings broader and forewing ground colour ochraceous, pattern even faded indistinct, antennae much slenderer.

**Figure 88:**
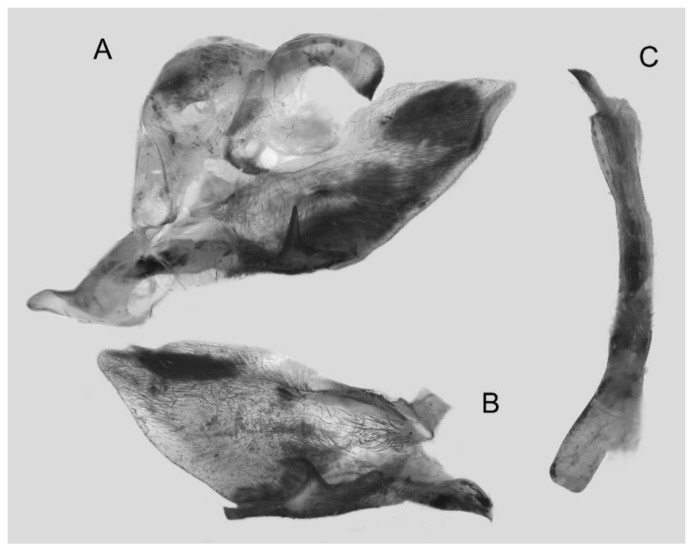
Male genitalia of *Ambulyx siamensis*, Xima Town, Yingjiang County, Yunnan. (**A**) Genital capsule with left valva removed; (**B**) left valva; (**C**) phallus.

Male genitalia (Figure 88A–C): Uncus and gnathos similar to *A. substrigilis*. Sacculus slender, dorsal margin with a large, sharply pointed and curved, triangular hook, harpe narrowed to a blunt finger-like lobe. Phallus slender and curved, posterior lobe sharply pointed apically and with tiny teeth laterally.

**Figure 89:**
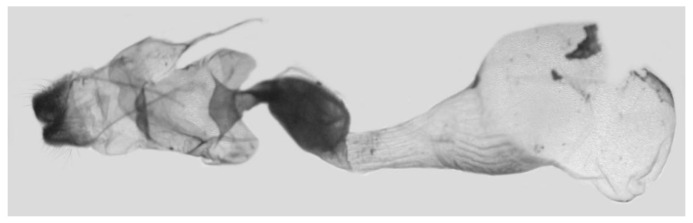
Female genitalia of *Ambulyx siamensis*, Chiang Mai, Thailand. © The Trustees of the Natural History Museum, London, UK (downloaded from Kitching, I.J. Sphingidae Taxonomic Inventory. http://sphingidae.myspecies.info/, accessed 20 April 2024.

Female genitalia (Figure 89): Similar to *A. substrigilis*. Antrum short, funnel-shaped, posterior margin wide and sinuate. Ductus bursae tubular, membranous, and long. Signum oval and covered with dense spines.

**Figure 90:**
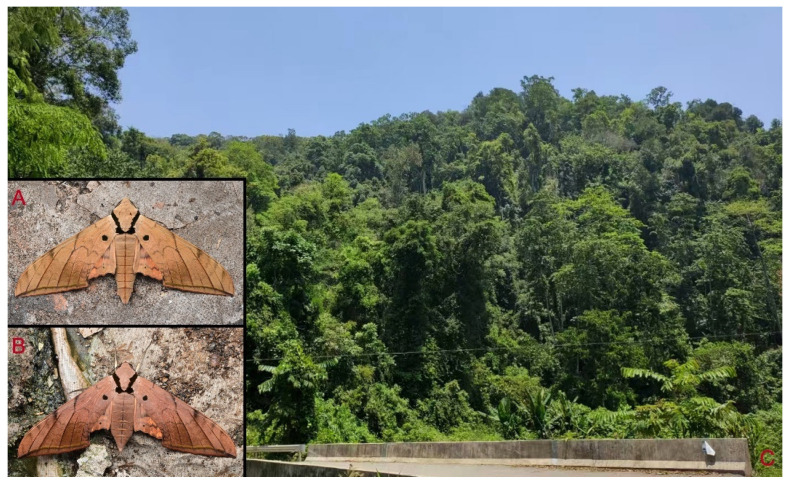
Habitat and living adults of *Ambulyx siamensis*. (**A**) Male; (**B**) female; (**C**) Mengla Couny, Yunnan, China.

**Biological notes:** This species was collected in low to mid-elevation subtropical forests, attracted to light at night or at rest on plants during the day (Figure 90).

**Distribution:** China (W. and S. Yunnan); Laos, Thailand, and Vietnam.

#### 3.2.24. *Ambulyx substrigilis* Westwood, 1847 [亚距鹰翅天蛾]

*Sphinx (Ambulyx) substrigilis* Westwood, 1847; The cabinet of Oriental entomology: [49], pl. 30, Figure 2; **Type locality:** Sylhet, Bangladesh.
  *Oxyambulyx substrigilis brooksi* Clark, 1923; Proceedings of the New England Zoological Club, 8: 52 [46].
  *Oxyambulyx sericeipennis subrufescens* Clark, 1936; Proceedings of the New England Zoological Club, 15: 73 [29].
  *Ambulyx substrigilis cana* Gehlen, 1940; Entomologische Zeitschrift, 54(18): 140 [50].

**Figure 91:**
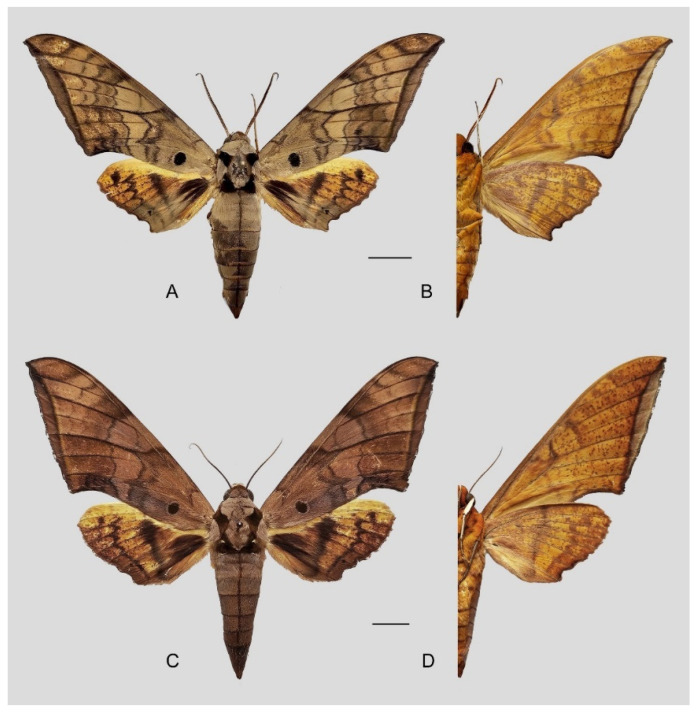
*Ambulyx substrigilis*. (**A**,**B**) Male, Mt. Jianfengling, Ledong County, Hainan; (**C**,**D**) female, Xiajinchang Township, Malipo County, Yunnan, China. Scale bar = 10 mm.

**Diagnosis:** Male (Figure 91A,B): Similar to *A. s. sericeipennis* but larger, body colour tends to be more greyish, abdomen upperside with a black dorsal line, without spots on any segment laterally. Forewing broader, upperside ground colour greyish-yellow, basal costal spot absent, subbasal black-green spot present, basal to medial area with conspicuous and heavier black-brown zigzag lines, subterminal line blackish, slightly arched and the line of yellowish-green scales along its inner edge generally fading before reaching the tornus, marginal area brownish, an indistinct blackish-green spot in the submarginal area between veins CuA_1_ and CuA_2_; underside with a well-marked black subterminal line that is weaker and brown or absent in *A. s. sericeipennis.* Hindwing upperside with a heavy zigzag line, a black spot near the apex, medial area with a wide black stripe and basal area with a conspicuous black patch.

Female (Figure 91C,D): Similar to male but wings broader and forewing ground colour ochraceous, patterns heavier, subterminal line weakly arched, marginal area brownish, antennae much slenderer.

**Figure 92:**
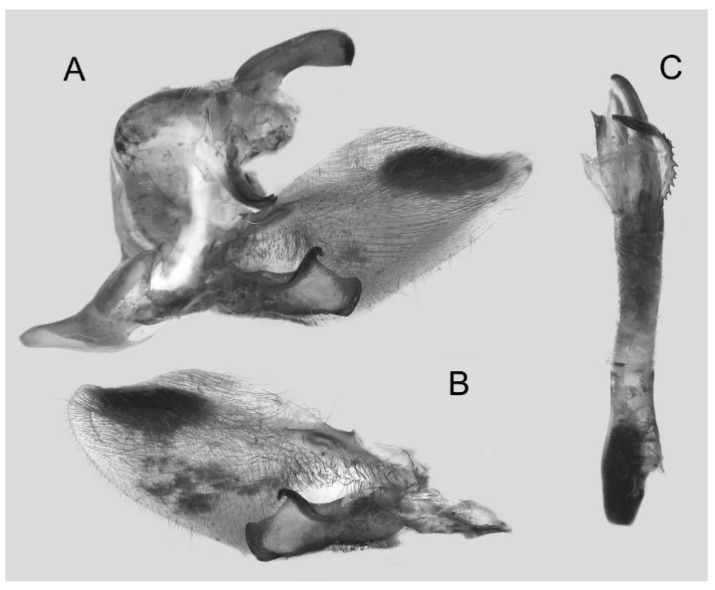
Male genitalia of *Ambulyx substrigilis*, Nabanhe Nature Reserve, Xishuangbanna, Yunnan, China. (**A**) Lateral view; (**B**) left valve; (**C**) phallus.

Male genitalia (Figure 92A–C): Similar to *A. kuangtungensis*. Uncus and gnathos somewhat heavy. Sacculus broader, dorsal margin with a single strongly curved and sharply pointed hook, harpe shovel-shaped and blunt. Phallus slender, posterior lobe with short, blunt and ventrally curved apical process, and a sharp tooth and a semi-circular serrated lobe laterally.

**Figure 93:**
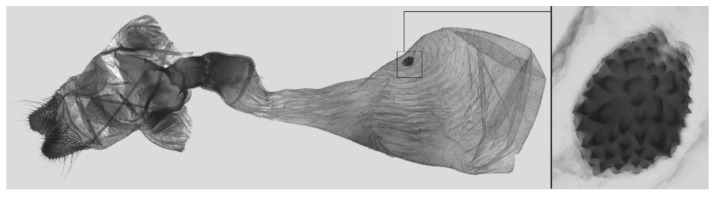
Female genitalia of *Ambulyx substrigilis*, Malipo County, Yunnan, China.

Female genitalia (Figure 93): Similar to *A. s. sericeipennis*. Antrum short and cylindrical. Signum oval without and anterior gap and covered with denser teeth.

**Figure 94:**
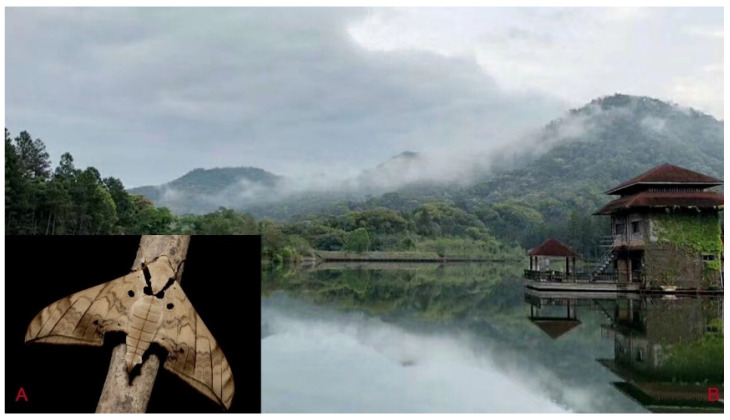
Habitat and living adult of *Ambulyx substrigilis*. (**A**) Male; (**B**) Jianfengling, Hainan, China.

**Biological notes:** This species was collected in mid-elevation monsoon evergreen broad-leaved forest and subtropical forests, attracted to light at night or found resting on plants during the day (Figure 94).

**Distribution:** China (Xizang, Yunnan, Guangxi, and Hainan); Sri Lanka, India, Nepal, Bhutan, Bangladesh, Thailand, Vietnam, Malaysia, Indonesia, and Philippines.

#### 3.2.25. *Ambulyx tattina tattina* (Jordan, 1919) [塔蒂鹰翅天蛾指名亚种]

*Oxyambulyx tattina* Jordan, 1919; Novitates Zoologicae, 26: 192; **Type locality:** Battak Mts, northeast Sumatra, Indonesia.
  *Ambulyx tattina borneensis* Gehlen, 1940; Entomologische Zeitschrift, 54(18): 140 [50].

**Figure 95:**
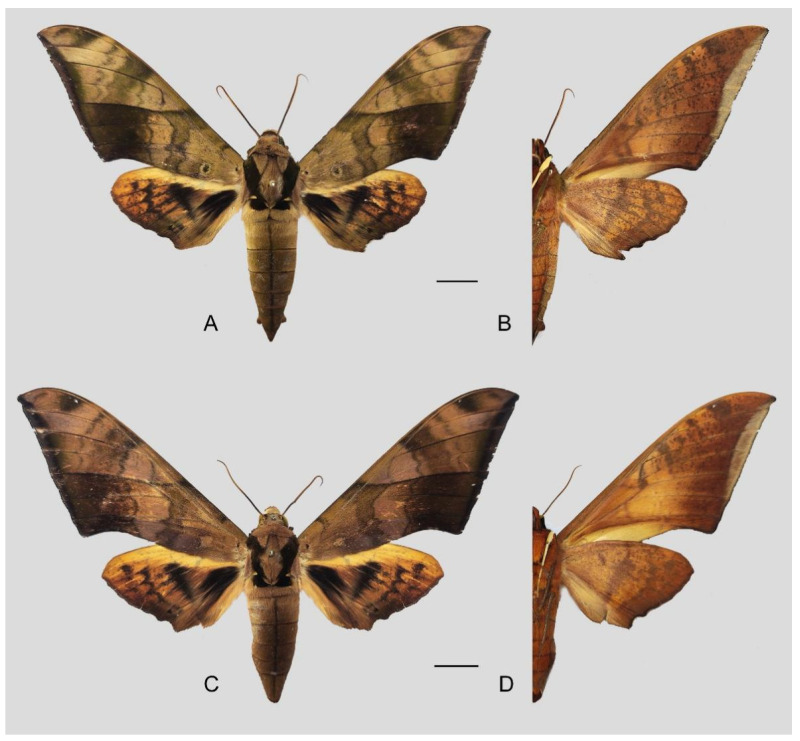
*Ambulyx tattina tattina*. (**A**,**B**) Male, Menghai County, Xishuangbanna, Yunnan. (**C**,**D**) Female, Menghai County, Xishuangbanna, Yunnan, China. Scale bar = 10 mm.

**Diagnosis:** Male (Figure 95A,B): Similar to *A. substrigilis*. Forewing upperside with heavier and darker brown-blackish zigzag lines and patches, with scattered greenish-brown scales, subbasal black spot minute (much smaller in *A. substrigilis*), surrounded by a characteristic pale ring, subterminal line wider, greenish-brown, arched and fading well before reaching the tornus, the line of yellowish-green scales along its inner edge absent, a conspicuous brownish-black patch in the submarginal area from vein M_1_ to the tornus, marginal area brown; underside ground colour ochre with dense scattered dark grey scales, subterminal line almost absent. Hindwing pattern darker and heavier than in *A. substrigilis*, especially the black medial stripe and zigzag line, and with more scattered black scales.

Female (Figure 95C,D): Similar to male but wings broader and forewing ground colour ochraceous and pattern much heavier, antennae much slenderer.

**Figure 96:**
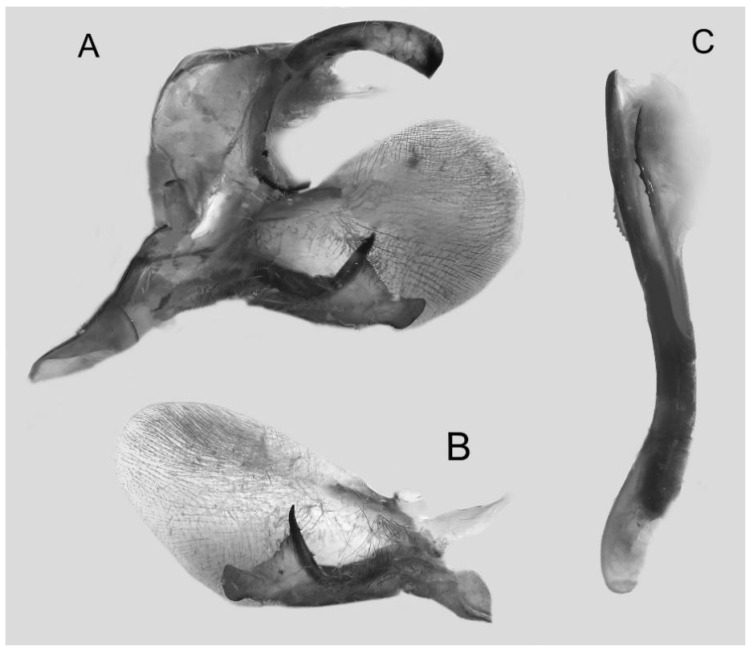
Male genitalia of *Ambulyx tattina tattina*, Menghai County, Xishuangbanna, Yunnan. (**A**) Lateral view; (**B**) left valve; (**C**) phallus.

Male genitalia (Figure 96A–C): Similar to *A. substrigilis*. Uncus slenderer and longer. Valve more rounded with smaller patch of friction scales. Process on dorsal margin of sacculus shorter and only slightly curved apically, harpe narrower than in *A. substrigilis*. Phallus longer, slenderer, and more curved than in *A. substrigilis*, posterior lobe much straighter and with a curved slender serrated lobe laterally on each side.

**Figure 97:**
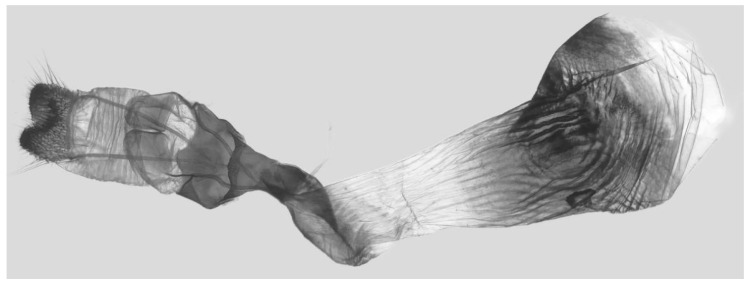
Feale genitalia of *Ambulyx tattina tattina*, Menghai County, Xishuangbanna, Yunnan.

Female genitalia (Figure 97): Similar to those of *A. substrigilis* but antrum shorter and broader. Signum oval, with a conspicuous posterior gap centrally.

**Figure 98:**
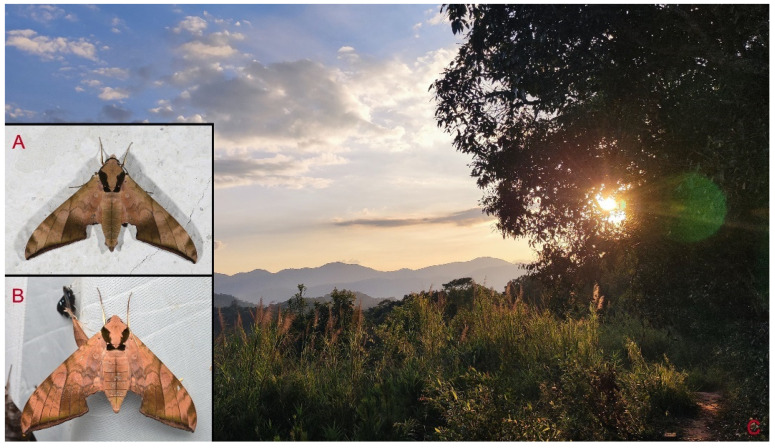
Habitat and living adults of *Ambulyx tattina tattina*. (**A**) Male; (**B**) female; (**C**) Pu’er, Yunnan, China.

**Biological notes:** This species was collected in mid-elevation monsoon evergreen broad-leaved forest and subtropical forests, attracted to light at night (Figure 98).

**Distribution:** China (South Yunnan); Laos, Thailand, Vietnam, Malaysia, and Indonesia.

#### 3.2.26. *Ambulyx tobii* Inoue, 1976 [拓比鹰翅天蛾]

*Oxyambulyx sericeipennis tobii* Inoue, 1976; Bull. Fac. domest. Sci. Otsuma Wom. Univ., 12: 173; **Type locality:** Awajishima, Sumoto City, Nakatsugawa, Japan.
  *Ambulyx sericeipennis pirika* Kishida, 2018; Japanese Heterocerists’ Journal, 287: 288 [38].

**Figure 99:**
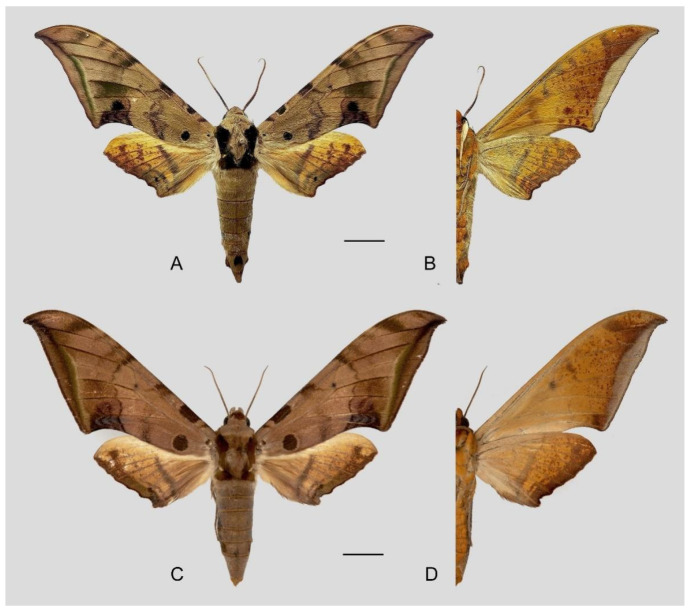
*Ambulyx tobii*. (**A**,**B**) Male, Yintiaoling Nature Reserve, Wuxi County, Chongqing, China; (**C**,**D**) female, Huairou County, Beijing. Scale bar = 10 mm.

**Diagnosis:** Male (Figure 99A,B): Very similar to *A. s. sericeipennis* but ground colour more greenish (especially in fresh Japanese specimens) although many continental moths are more yellowish-grey, dorsal V-shaped patch on thorax blackish green rather than the brownish green of *A. s. sericeipennis*, a blackish green lateral spot on each side of abdominal segments 6 and 7. Forewing upperside ground colour yellowish-green or yellowish-grey, with scattered brown scales, subbasal and basal costal black spots generally larger than in *A. s. sericeipennis*, subterminal line more arched and the line of green or yellowish-green scales along its inner edge generally fading by vein M_1_ whereas in *A. s. sericeipennis* this line extends across vein M_1_ even reaching the apex, the blackish-green spot in the submarginal area between veins CuA_1_ and CuA_2_ generally larger than in *A. s. sericeipennis*.

Female (Figure 99C,D): Similar to male but wings broader and ground colour darker, yellowish-green area on forewing upperside much broader, antennae much slenderer.

**Figure 100:**
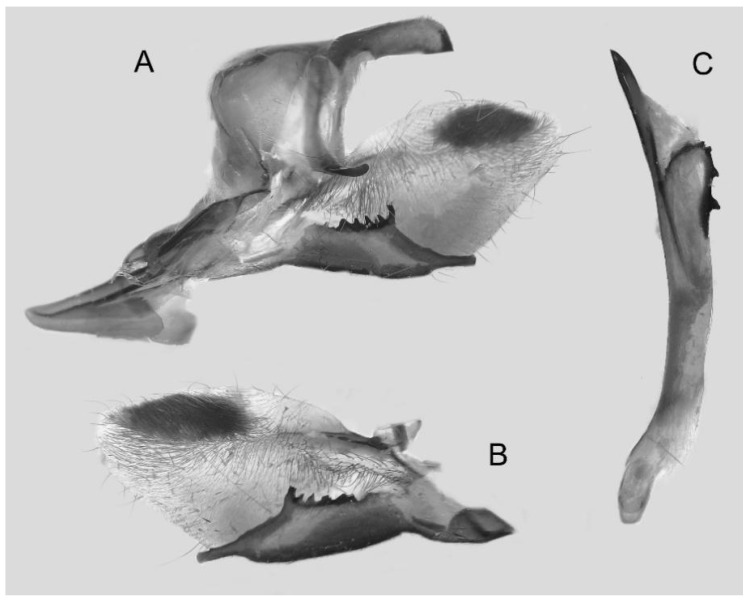
Male genitalia of *Ambulyx tobii*, Mt. Leigongshan, Suiyang County, Guizhou, China. (**A**) Lateral view; (**B**) left valve; (**C**) phallus.

Male genitalia (Figure 100A–C): Similar to *A. s. sericeipennis* but sacculus broader. Phallus much longer, posterior lobe apically sharp, dorsal margin with fewer teeth.

**Figure 101:**
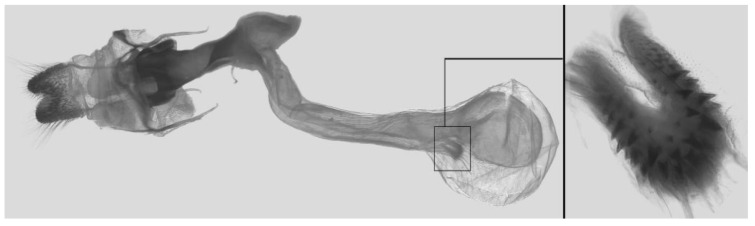
Female genitalia of *Ambulyx tobii*, Yingshan County, Hubei, China.

Female genitalia (Figure 101): Similar to those of *A. s. sericeipennis*. Ductus bursae tubular, membranous and longer. Signum tongue-shaped, slenderer than in *A. s. sericeipennis* and covered with more and denser spines and teeth.

**Figure 102:**
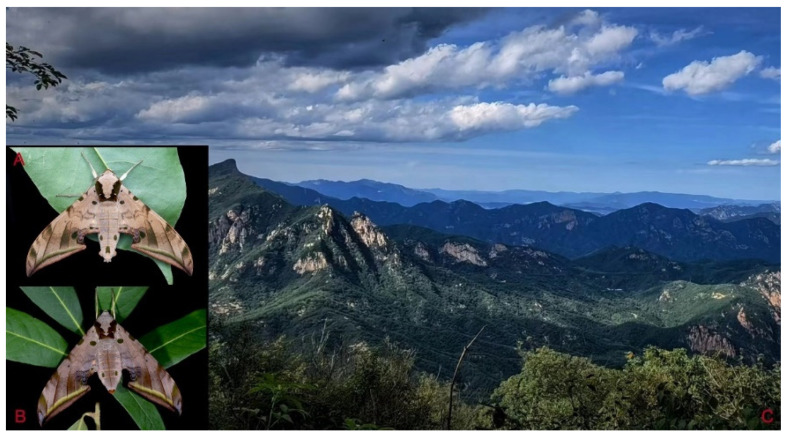
Habitat and living adults of *Ambulyx tobii*. (**A**) Male; (**B**) female; (**C**) Huairou County, Beijing, China.

**Biological notes:** This species was collected in low to high elevation monsoon evergreen broad-leaved forest attracted to light at night or found resting on plants during the day (Figure 102).

**Distribution:** China (Beijing, Tianjin, Shaanxi, Zhejiang, Hubei, Sichuan, Chong-qing, Yunnan, Xizang, Guizhou, Hunan, Fujian, Taiwan, Guangdong, and Guangxi); Bhutan, India, Vietnam, North Korea, South Korea, Japan, and Russia.

#### 3.2.27. *Ambulyx zhejiangensis* Brechlin, 2009 [浙江鹰翅天蛾]

*Ambulyx zhejiangensis* Brechlin, 2009, Entomo-Satsphingia, 2(2): 50; **Type locality:** Yu-Shan-Wu, Anji County, Zhejian, China.

**Figure 103:**
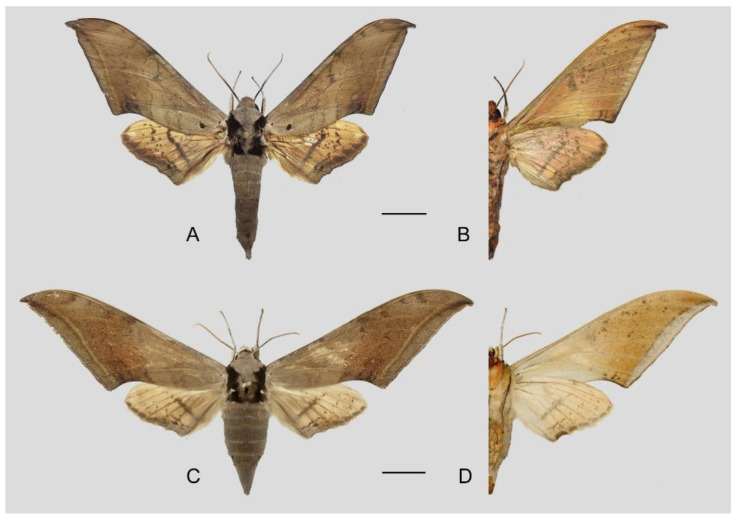
*Ambulyx zhejiangensis*. (**A**,**B**) Male, Yintiaoling Nature Reserve, Wuxi County, Chongqing, China; (**C**,**D**) female, Tapa Shan, China. © Sphingidae Museum, Czech Republic. Scale bar = 10 mm.

**Diagnosis:** Male (Figure 103A,B): Head—grey; thorax—grey with wide black patches and a V-shaped dorsal black patch; abdomen—upperside greyish, tergum A8 with a dark patch dorsally and a tiny black spot on each side of segment 6 laterally. Forewing long, triangular with sharp apex, outer margin smooth, distal part of inner margin slightly concave; upperside ground colour grey with scattered pale brown scales, subbasal black spot minute, medial area with indistinct pale brown zigzag lines, subterminal line pale brown, straight and reaching the inner margin just basad of the tornus (as in the *A. placida* group), with a line of yellowish-green scales along its inner edge; underside—ground colour brownish-yellow, dotted with grey spots, marginal area greyish. Hindwing—upperside yellow with tiny brown spots, a brown curved line in the medial area and a brown zigzag line in the submarginal area, marginal area greyish-brown; underside—ground colour brownish-yellow scattered with pink scales, pattern similar to that on the upperside.

Female (Figure 103C,D): Similar to male but wings broader and pattern more indistinct, antennae much slenderer.

**Figure 104:**
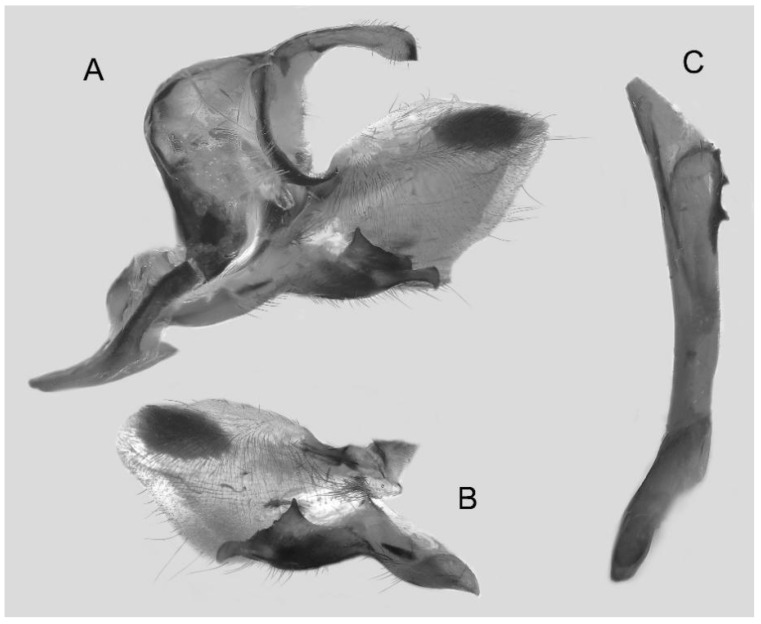
Male genitalia of *Ambulyx zhejiangensis*, Wuxi County, Chongqing, China. (**A**) Lateral view; (**B**) left valve; (**C**) phallus.

Male genitalia (Figure 104A–C): Similar to *A. kuangtungensis*, with a more rounded valve. Sacculus heavier and broader, dorsal margin with a broad, bluntly pointed, triangular lobe, harpe strongly narrowed basally, apex a thick sharp downwards-directed hook. Phallus slender, posterior lobe slender, straight and apically sharply pointed, with a few large teeth ventrally.

Female genitalia: Not examined.

**Figure 105:**
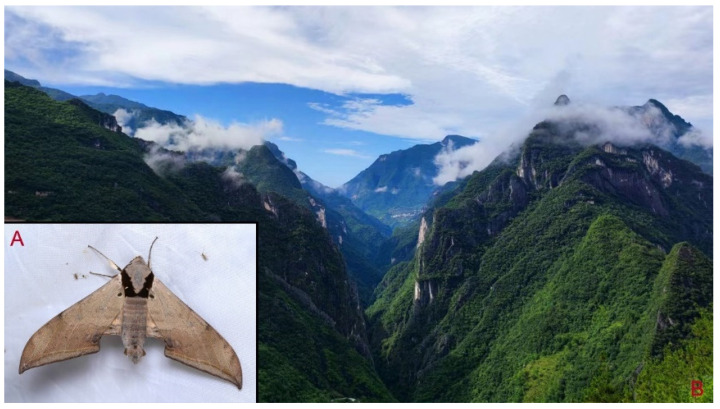
Habitat and living adult of *Ambulyx zhejiangensis*. (**A**) Male; (**B**) Wuxi County, Chongqing, China.

**Biological notes:** This species was collected in mid-elevation monsoon evergreen broad-leaved forests attracted to light at night (Figure 105).

**Distribution:** Currently known only from China (Chongqing, Hubei, and Zhejiang).

### 3.3. Biological Records of Habitats and Host Plants

**Figure 106:**
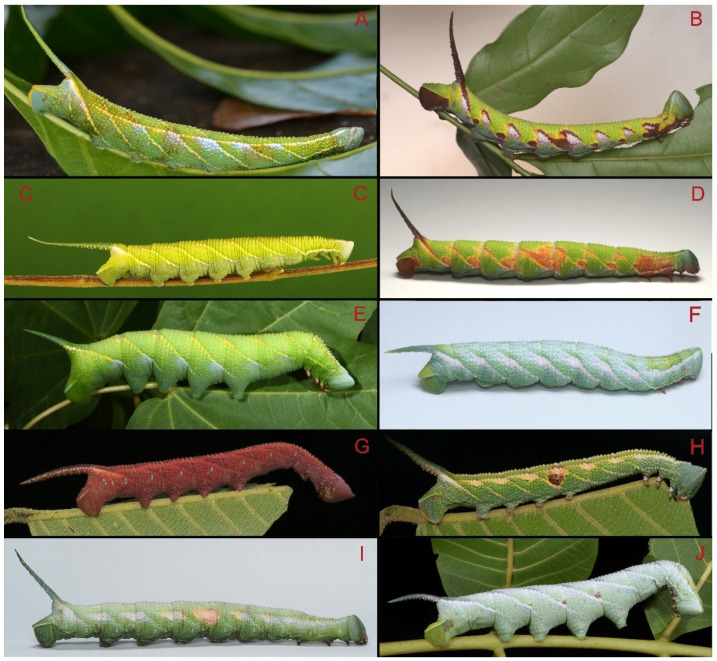
Larvae of *Ambulyx* species from China. (**A**) *A*. *moorei*, Foshan, Guangdong, China; (**B**) *A. sericeipennis sericeipennis*, Hongkong, China; (**C**) *A. ochracea*, Guangzhou, Guangdong, China; (**D**) *A. kuangtungensis*, Guangzhou, Guangdong, China; (**E**) *A. semiplacida montana*, Pingbian County, Yunnan, China; (**F**) *A. schauffelbergeri*, Chongqing City, China; (**G**) *A. siamensis*, Mengla County, Yunnan, China; (**H**) *A. siamensis*, Mengla County, Yunnan, China; (**I**) *A. liturata*, Mt. Maoershan, Guangxi, China; (**J**) *A. tobii*, Kunming, Yunnan, China.

Larvae of the Chinese *Ambulyx* species (Figure 106) have been recorded feeding on various woody plants. *Ambulyx canescens* is recorded on *Dipterocarpus tuberculatus*, *Dryobalanops lanceolata*, *Shorea lepidota*, and *Shorea macroptera*; *A*. *japonica koreana* on species of *Acer*, *Carpinus japonica*, *Carpinus tschonoskii*, and *Carpinus laxiflora*; *A. kuangtungensis* on *Choerospondias axillaris*, *Pistacia chinensis*, and *Elaeocarpus rugosus; A. liturata* on *Castanopsis hystrix*, *Canarium album*, and *Quercus*; *A*. *moorei* on *Canarium album*, *Buchanania*, *Lannea*, and various Anacardiaceae; *A. ochracea* on *Choerospondias axillaris*, *Juglans regia*, *Rhus chinensis*, and *Platycarya strobilacea*; *A. schauffelbergeri* on *Juglans regia*; *A. semiplacida semiplacida* on *Pistacia chinensis*; *A. semiplacida montana* on *Acer sinense*; *A. sericeipennis sericeipennis* on various plants, such as *Juglans regia*, *Engelhardia spicata*, *Engelhardia roxburghiana*, *Quercus*, *Myrica nagi*, *Betula alnoides* and *Rhus chinensis*; *A. tobii* on *Juglans regia*, *Acer mandshuricum*, and *Acer pictum*; *A. siamensis* on *Parashorea chinensis* and *Shorea obtusa*; and *A. substrigilis* on *Aglaia littoralis* [40,51,52,53,54,55,56,57].

**Figure 107:**
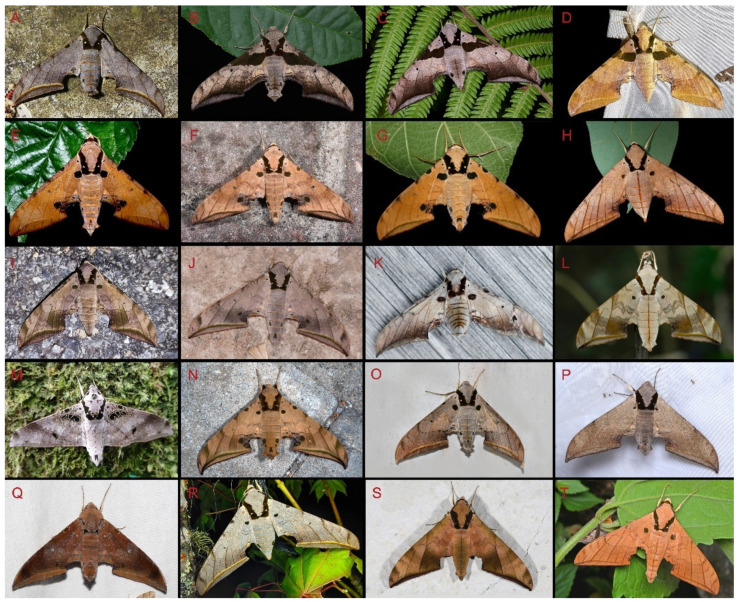
Morphological comparison of living adults of *Ambulyx* species from China. (**A**) Male *A*. *semiplacida interplacida*, Mt. Leigongshan, Guizhou, China; (**B**) male *A*. *japonica koreana*, Wuxi County, Chongqing, China; (**C**) female *A*. *japonica angustifasciata*, Nantou County, Taiwan, China; (**D**) female *A*. *latifascia*, Hutiaoxia, Yunnan, China; (**E**) Male *A*. *kuangtungensis*, Wuxi County, Chongqing, China; (**F**) male *A*. *maculifera*, Motuo, Xizang, China; (**G**) male *A*. *ochracea*, Wuxi County, Chongaqing, China; (**H**) female *A*. *liturata*, Shenzhen, Guangdong, China; (**I**) Female *A*. *schauffelbergeri*, Yuexi, Anhui, China; (**J**) male *A*. *sericeipennis sericeipennis*, Zunyi, Guizhou, China; (**K**) male *wukong* Jiang and Kitching sp. nov., Weixi, Yunnan, China; (**L**) male *A*. *substrigilis*, Haikou, Hainan, China; (**M**) male *A*. *canescens*, Yingjiang, Yunnan, China; (**N**) male *A*. *tobii*, Huairou County, Beijing, China; (**O**) male *A. placida*, Motuo, Xizang, China; (**P**) male *A*. *zhejiangensis*, Wuxi County, Chongqing, China; (**Q**) male *A*. *moori*, Hezhou, Guangxi, China; (**R**) female *A. semiplacida montana*, Pingbian County, Yunnan, China; (**S**) male *A*. *tattina tattina*, Mengla County, Yunnan, China; (**T**) male *A*. *siamensis*, Menglun, Xishuangbanna, Yunnan, China.

**Figure 108:**
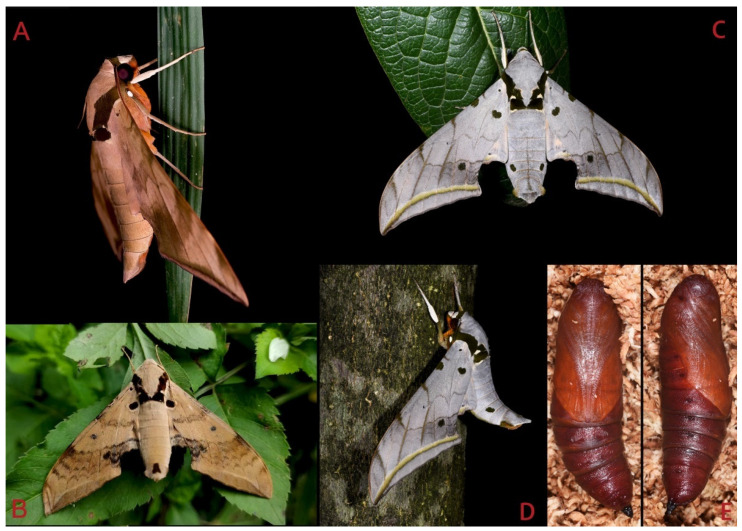
(**A**) *A*. *tattina tattina* resting after emerging in the rainforest in Mengla County, Yunnan, China; (**B**) an *A*. *kuangtungensis* at rest by day in the forest in Yuanjiang County, Yunnan, China; (**C**,**D**) *A. semiplacida montana* resting after emerging in the forest in Pingbian County, Yunnan, China; (**E**) Pupa of the *A. semiplacida montana* from Pingbian County, Yunnan, China.

**Figure 109:**
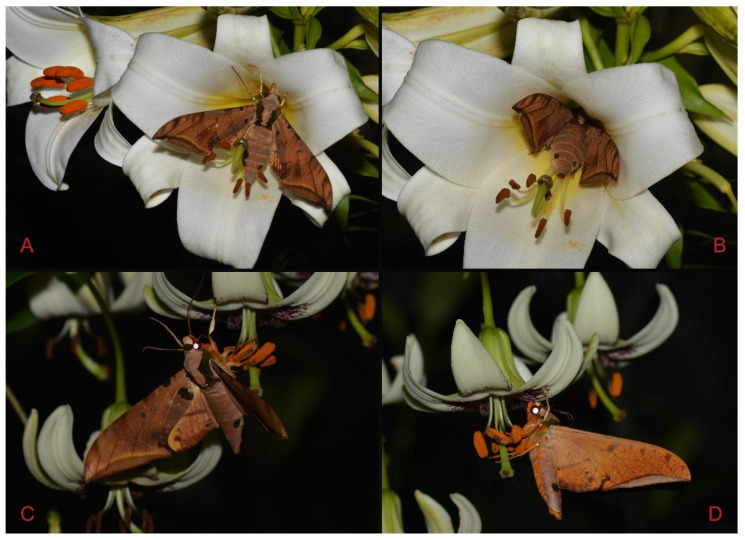
Some species of *Ambulyx* from China visiting flowers at night. (**A**,**B**) Female *A*. *tobii* visiting *Lilium sulphureum*, Yanhe County, Guizhou, China; (**C**,**D**) female *A. ochracea* visiting *Lilium primulinum* var. *ochraceum*, Guiyang City, Guizhou, China.

**Figure 110:**
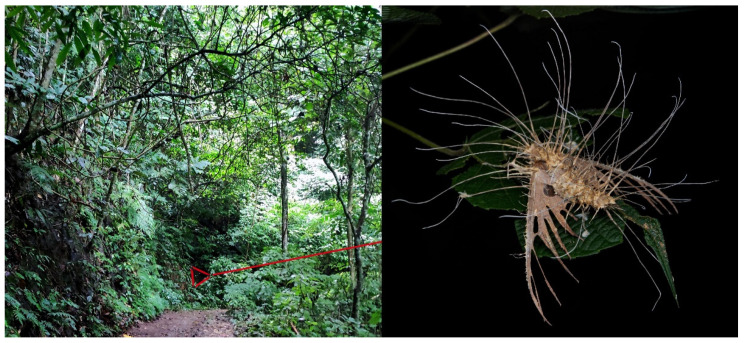
*Ambulyx liturata* infested by *Akanthomyces* sp. in the rainforest of Mengla County, Xishuangbanna, Yunnan, China (the red line indicate its concealed position in rainforest).

Adult *Ambulyx* are nocturnal hawkmoths. According to our observations and collecting experience, males are easily attracted to lights, but the females of some species were very rarely attracted to light traps (Figure 107), for example, the *placida*-group species, *A. siamensis*, *A. substrigilis*, *A. zhejiangensis*, *A. latifascia* and *A. maculifera*. Sometimes, adult *Ambulyx* species can be observed resting on the leaves or hanging on branches during the day (Figure 108) or visiting flowers, such as those of *Lilium* and *Magnolia*, at night (Figure 109). Occasionally, they have been observed infested with entomopathogenic fungi, such as *Akanthomyces* sp. (Figure 110).

### 3.4. Morphological Differences

**Figure 111:**
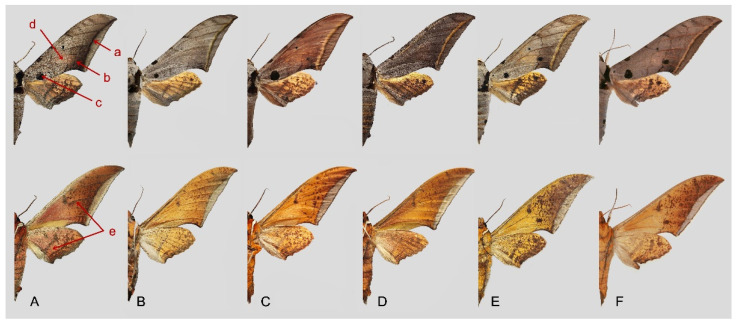
Morphological comparison of species of the *Ambulyx placida*-group (**A**) *A*. *wukong* sp. nov., Wexi, Yunnan, China, male; (**B**) *A*. *placida*, Nyalam County, Xizang, China, male; (**C**) *A. semiplacida semiplacida*, Pingtung, Taiwan, China, male; (**D**) *A. semiplacida interplacida*, Shaoguan, Guangdong, China, male; (**E**) *A. semiplacida montana*, Pingbian, Yunnan, China, male; (**F**) *A. semiplacida bhutana*, Trongsa Dzong, Bhutan, male. © Ronald Brechlin. Uppersides in the top row, undersides in the bottom row.

**Figure 112:**
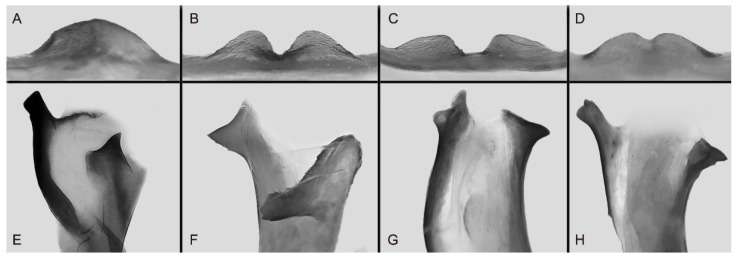
Male genitalia of the *Ambulyx placida*-group. (**A**) Gnathos of *A*. *placida*, Nyingchi, Xizang, China; (**B**) Gnathos of *A*. *s. semiplacida*, Taitung, Taiwan, China; (**C**) Gnathos of *A. wukong* sp. nov., Wexi, Yunnan, China; (**D**) Gnathos of *A. japonica koreana*, Wuxi, Chongqing, China; (**E**) Phallus lobe of *A*. *placida*, Nyingchi, Xizang, China; (**F**) phallus lobe of *A*. *s. semiplacida*, Taitung, Taiwan, China; (**G**) phallus lobe of *A. wukong* sp. nov., Wexi, Yunnan, China, China; (**H**) phallus lobe of *A. japonica koreana*, Wuxi, Chongqing, China.

**Figure 113:**
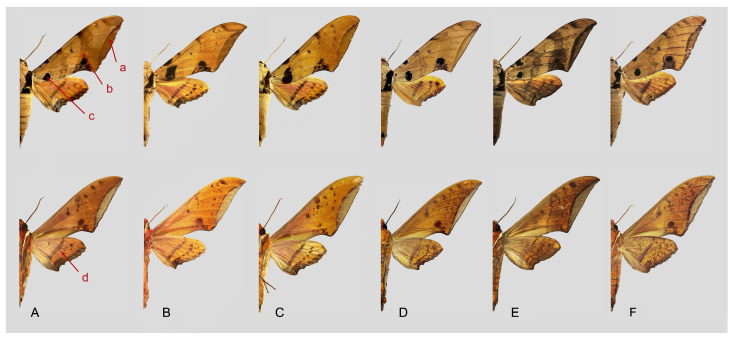
Morphological comparison of similar species of *Ambulyx* of China (**A**) *A. kuangtungensis*, Libo, Guizhou, China, male; (**B**) *A. kuangtungensis f. adhemariusa*, Yuexi, Anhui, China, male; (**C**) *A. latifascia*, Panzhihua, Sichuan, China, male; (**D**) *A. ochracea*, Hangzhou, Zhejiang, China, male; (**E**) *A. schauffelbergeri*, Nanjing, Jiangsu, China, male; (**F**) *A. maculifera*, Yingjiang, Yunnan, China, male. Uppersides in the top row, undersides in the bottom row.

**Figure 114:**
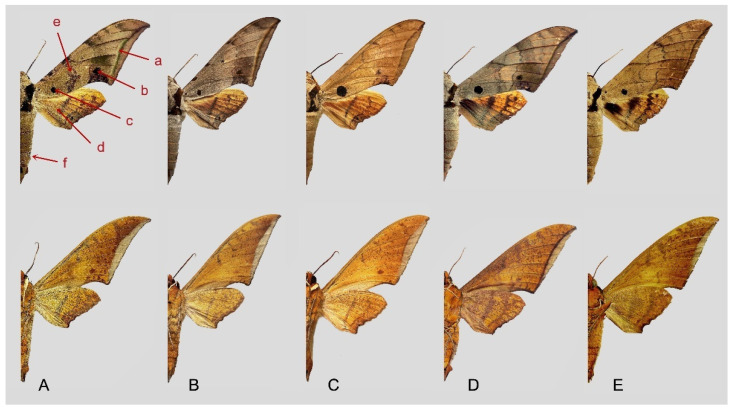
Morphological comparison of similar species of *Ambulyx* of China (**A**) *A*. *tobii*, Wuxi, Chongqing, China, male; (**B**) *A. sericeipennis sericeipennis*, Libo, Guizhou, China, male; (**C**) *A. liturata*, Sanming, Fujian, China, male; (**D**) *A. substrigilis*, Mengla, Yunnan, China, male; (**E**) *A. siamensis*, Mengla, Yunnan, China, male. Uppersides in the top row, undersides in the bottom row.

Comparison of the wing morphology of sets of similar species of *Ambulyx* from China showed constant differences, as illustrated in Figure 111, Figure 112, Figure 113 and Figure 114. The following characters are important in separating similar species of the *placida*-group (Figure 111): (1) the subterminal line on the forewing upperside in *A. wukong* sp. nov. is black whereas in other species it is grey-brown with scattered yellow-greenish scales along the edge (a); (2) the submarginal area of the forewing upperside from vein M_3_ to the tornus is covered with dark blackish-brown scales in *A. wukong* sp. nov. but this area is covered with grey or purple-brown scales in the other species and subspecies (b); (3) *A. wukong* sp. nov. and *A. semiplacida semiplacida* have a large, circular, and conspicuous basal spot on the forewing upperside whereas in the other species and subspecies although it varies in size, it is generally smaller, and in *A. placida* and *A. semiplacida interplacida* this basal spot is sometimes tiny or even lacking (c); (4) the ground colour of the forewing upperside in *A. placida* and *A. semiplacida montana* is generally paler than in the other species and subspecies, and *A. semiplacida interplacida* is the most indistinct and uniformly patterned (d); and (5) the ground colour of the forewing and hindwing undersides is ochre in *A. wukong* sp. nov. but more yellow in the other species and subspecies; *A. wukong* sp. nov. has the densest scattered black-grey scaling of all the species (e).

The male genital structures (Figure 112) are also important in separating some similar species of the *placida*-group: (1) *A. placida* has an unnotched gnathos (A) and a tiny tooth distally on the apex of the phallus without a strong, laterally projecting tooth (E); (2) *A. semiplacida*, *A. japonica* and *A. wukong* sp. nov. all have a bilobed gnathos (B–D) and a phallus with a strong laterally projecting tooth (F–H) of which *A. japonica* has the least bilobed gnathos; and (3) in the subspecies of *A. semiplacida*, ssp. *semiplacida* has a heavier and broader phallus than other subspecies and ssp. *interplacida* has the least bilobed gnathos.

Among the five similar middle-size species, the following characters are important in separating them (Figure 113): (1) the dark brown subterminal line on forewing upperside in *A. kuangtungensis*, *A. latifascia* and *A. ochracea* is inconspicuous and fades before reaching the tornus, whereas in other two species this line is broader and black, and reaches the anal margin, especially so in *A. schauffelbergeri* (a); (2) the transverse lines in the medial area appear as indistinct dotted lines in *A. kuangtungensis* and *A. latifascia*, but as zigzag lines in other three species, with *A. schauffelbergeri* having the heaviest patterns and a denser scattering of dark brown scales (b); (3) *A. latifascia* has a large, black-greenish patch in the basal area that almost connects with the black patch on the costa, while in the other species this basal spot is a circular and conspicuous (although *A. kuangtungensis* can show a similar pattern to *A. latifascia* and such moths can be difficult to identify, for example, see Figure 113B and several specimens illustrated at [2] (https://sphingidae.myspecies.info/taxonomy/term/428/media, accessed on 20 April 2024) (c); and (4) the forewing and hindwing undersides of *A. kuangtungensis* and *A. latifascia* have less dense scattered black-grey scaling than the other species and the basal third has scattered pink scales rather than the yellow sales of the other three species (d).

The following characters are important in separating the five similar large-sized species (Figure 114): (1) the dark brown subterminal line on forewing upperside in *A. tobii* and *A. s. sericeipennis* is more conspicuous, with a much broader green-yellowish scaled area than in other species, and reaches the tornus whereas this line is narrower and fades before reaching the tornal angle in the other three species (a); (2) the black spots in the submarginal area between veins CuA_1_ and CuA_2_ are generally conspicuous and larger in *A. tobii* but indistinct or inconspicuous in the other species, even disappearing in *A. siamensis* (b); (3) *A. liturata* has the largest basal black spot whereas it is much smaller in other four species, sometimes being only a tiny dot, or even disappearing in *A. siamensis* and *A. s. sericeipennis* (c); (4) on hindwing upperside, *A. substrigilis* and *A. siamensis* have a conspicuous black basal patch and a heavy black medial stripe and zigzag postmedial line whereas these patterns are much weaker and paler in the other three species, *A. tobii* and *A. s. sericeipennis* lacking any black basal patch and *A. liturata* having a tiny and indistinct black basal patch (d); (5) on forewing upperside, *A. liturata* and *A. siamensis* generally have the most indistinct and uniform pattern, *A. substrigilis* is the most strongly patterned species especially the dark zigzag transverse lines and patches, the ground colour of *A. substrigilis* and *A. s. sericeipennis* tends to be more grey than in the other species, and *A. tobii* and *A. s. sericeipennis* have a conspicuous black-greenish basal spot on the costa that is lacking in other three species (e); and (6) in *A. liturata*, *A. substrigilis* and *A. siamensis*, the abdomen has a conspicuous black-brownish dorsal line and lacks any lateral spots on segments 6 and 7, whereas in *A. tobii* and *A. s. sericeipennis* the dorsal abdominal line is indistinct and there is a black-greenish spot laterally on the each side of segments 6 and 7 (f). It is not easy to distinguish *A. tobii* and *A. s. sericeipennis*, but the descriptions of these two species in the taxonomic section above offer some apparently stable features.

## 4. Discussion

In the mountainous areas of Asia, there is a group of hawkmoths, the *Ambulyx placida*-group, that are fairly homogeneous in size and pattern. These large *Ambulyx* species are usually greyish in colour, sometimes tending towards yellowish or purplish. A common feature of these moths is the presence on the forewing upperside of a broad, subterminal line that from the apex and to the tornus that is generally brown or black with scattered with green scales when the insect is fresh but fading to greenish yellow in older specimens. It is important to note that this line ends on the inner margin, slightly basad of the tornus, whereas in the similar species, *A. sericeipennis*, and also most other *Ambulyx* species, it either ends at or disappears before reaching the tornus. This is the main characteristic of *placida*-group that enables its species to be distinguished easily from other *Ambulyx* species-groups. The morphology of the male genital structures, as well as body and wing patterns, is consistent with the molecular evidence based on seven male specimens, in assigning a fourth and new species, *A. wukong* sp. nov., to the *placida*-group. It is only known from high elevation (2100–2500 m) forest in the Hengduan Mountains, Weixi, Yunnan. Phylogenetic analysis places it as the sister to the remaining three species (Figure 4). The centre of diversity of the *placida*-group appears to be in the East Himalaya, towards to Hengduan Mountains (Figure 1) but this needs further verification from future whole genome sequencing; here, we could only use DNA barcode sequences (*cox1*) due to limited sampling. It is sometimes not easy to distinguish some of the species of the *placida*-group because they have a great variation in colouration and pattern. Thus, recourse must be made to important diagnostic characteristics in the male genital structure. In *A. placida*, the gnathos is unnotched and the phallus lacks a strong, laterally projecting tooth, whereas *A. semiplacida*, *A. japonica* and *A. wukong* sp. nov. all have an obviously bilobed gnathos and a laterally projecting tooth on the phallus [58] (Figure 111). The phylogenetic tree (Figure 4) and K2P distance (Figure 5A) also suggests that some taxa in this group, such as *A. s. bhutana* and *A. s. montana*, need further study due to their extremely close relationship.

The *schauffelbergeri*-group is another interesting group of fairly similar species in size and pattern. These middle to large *Ambulyx* species are usually greyish-yellow or brownish-yellow in colour, sometimes tending towards ochreous. According to our phylogenetic analyses, the *schauffelbergeri*-group comprises four species: *A. lahora*, *A. tobii*, *A. schauffelbergeri* and *A. sericeipennis*. The last three species are all similar in morphology and are frequent in most areas of China. *Ambulyx lahora* is restricted to western Himalayan regions, mainly in Nepal, NW India and Pakistan [2,7] and is also the most westerly occurring species of the genus. The four insular subspecies of *A. sericeipennis*, *A. s. joiceyi*, *A. s. javanica*, *A. s. luzoni* and *A. s. palawanica* [16,46,47,48] (Figure 2), together with the nominotypical subspecies, all comprise a single DNA barcode BIN and so assigning species or subspecies status to them is somewhat subjective (Figure 4). This species needs further study; we only had limited DNA barcode sequences (*cox1*) to reconstruct their relationships and maybe whole genome sequencing would provide better insights.

*Ambulyx zhejiangensis* is one of the rarest *Ambulyx* species in China, with only a few specimens known: the holotype female from “Yu-Shan-Wu, Anji county, Zhejiang, China”; a female from the same locality as the holotype; a female from “Tapa Shan, China” (probably Hubei); a male without any information illustrated in original description [16]; a male from Yasunori Kishida’s (Japan) collection; and a male collected from Yintiaoling Nature Reserve, Chongqing, China collected for this study. *A. zhejiangensis* is similar to the sympatric *A. sericeipennis* but the forewing ground colour is much paler, the pattern of transverse lines and bands is almost obsolete, and the wing shape is more curved, especially the female. Dissection of the male genitalia of *A. zhejiangensis* showed they are similar to those of *A. kuangtungensis* in having a similar sacculus structure, with a hook-shaped harpe and a tiny upwardly directed point medially on the dorsal margin. *Ambulyx zhejiangensis* is so far known only from the mountains of Zhejiang, Hubei, and Chongqing provinces [51] (Figure 2), but its distribution probably covers a greater area than these records suggest. Interestingly, the phylogenetic tree suggests this species is close to *A. maculifera* and that these two species form a clade (Figure 4), but they are not similar in morphological characters such as the wing patterns and genital structure.

China is a country with a mega-biodiversity of hawkmoths, especially in west and south Yunnan, where the physical environment is highly heterogeneous. In recent years, many new hawkmoth taxa have been described and new records made in southwest China [51,59,60,61,62,63,64]. Most species of *Ambulyx* are forest species, and in China, a number of subtropical species are only found in south Yunnan, such as *A. tattina* and *A. semiplacida montana*. The former is found in Thailand, Malaysia, Borneo, Sumatra, Java, and the Philippines [2,65,66], but our record from Xishuangbanna in Yunnan is the first record for China; the latter is found in Laos, Vietnam, Thailand, Myanmar, and India [58,67], and our record from Pingbian in Yunnan is the first Chinese record of this subspecies. Some of the rare Chinese *Ambulyx* species are difficult to observe and collect, perhaps because SE and S Yunnan is on the northernmost edge of their ranges and their population densities appear to be much lower than in SE Asia. For example, *A. canescens* is a very common species in Myanmar, Thailand, Vietnam, Cambodia, Malaysia, and Indonesia [51,68] but there are only two records in China, from W Yunnan [51]. In summary, the genus *Ambulyx* is now represented in China by 19 species and ten species-groups (Figure 4), but it still needs further investigation, particularly in those areas near the borders with neighbouring countries.

## Data Availability

The data are openly available in GenBank at https://www.ncbi.nlm.nih.gov/genbank/ and BOLD SYSTEMS https://v4.boldsystems.org/ (accessed on 14 December 2024). A list of investigated species and their GenBank accession numbers or BOLD sampleIDs is given in Table S1.

## Supplementary Materials

The following supporting information can be downloaded at: https://www.mdpi.com/article/10.3390/insects16020223/s1, Table S1: Sampling information and BOLD SampleID/GenBank accession numbers of Ambulyx and outgroups samples used in this study. The taxon names follow current taxonomy.

## Appendix A. List of Ambulyx Specimens Examined

Names of institutions in which specimens are deposited are listed after private collections and are abbreviated as follows: ZHJ—collection of Zhuo-Heng Jiang (Kunming, China); ZBX—collection of Zhen-Bang Xu (Beijing, China); CQL—collection of Chang-Qiu Liu (Guilin, China); DRC—collection of De-Rao Chou (Shanghai, China); KIZAS—collections of the Kunming Institute of Zoology, Chinese Academy of Science (Kunming, Yunnan, China); NHMUK—collection of the Natural History Museum (London, U.K.); NWAU—collections of the Northwest Agriculture and Forestry University (Shaanxi, China); SM—collections of the Sphingidae Museum (Příbram, Czech Republic).

***Ambulyx canescens* (Walker, 1865)**

♂, Xima Town (1500 m), Yingjiang County, Yunnan, China, 2018 VII-23, Zhuo-Heng Jiang *leg*. [ZHJ]; ♂, Rare Botanical Garden (1200 m), Ruili, Yunnan, China, 2007-VII-2, Xiao-Chen Zhang *leg*. [NWAU].

***Ambulyx japonica angustifasciata* Okano, 1959**

2♂♂, Donghe Township (560 m), Taitung County, Taiwan, China, 2018 VII-2, Wen-Yi Chou *leg*. [ZHJ]; 5♂♂, Nantou County, Taiwan, China, 2023 V-22, Local collector *leg*. [ZHJ]; 3♂♂, Puli, Nantou County, Taiwan, China, VI-8, Schnitzler *leg*. [SM]; ♀, Puli, Nantou County, Taiwan, China, VI-3, Schnitzler *leg*. [SM].

***Ambulyx japonica koreana* Inoue, 1993**

4♂♂, ♀, Yintiaoling Nature Reserve (1270 m), Wuxi County, Chongqing, China, 2022 VII-23, Zhuo-Heng Jiang *leg*. [ZHJ]; 4♂♂, 2♀♀, Ziqiu Township (1473 m), Changyang County, Hubei, China, 2022 V-30, Zi-Hao Shen *leg*. [ZHJ]; ♂, ♀, Mt. Wuyishan (1500 m), Jiangxi, China, 2005-VI-VII, Zhi-Yuan Qi *leg*. [SM]; ♂, Mt. Jiajinshan (1800 m), Xiaojin County, Sichuan, China, 2016 V-3, Zhi-Yuan Qi *leg*. [ZHJ]; ♀, Mentougou (1210 m), Beijing, China, 2018 VII-13, Kun Li *leg*. [ZHJ]; ♀, Baotianman (1170 m), NanYang, Henan, China, 2020 VI-19, Dan-Dan Xu *leg*. [ZHJ].

***Ambulyx wukong* Jiang & Kitching sp. nov.**

**HOLOTYPE:** ♂, Tacheng (2350 m), Weixi County, Yunnan, China, 2023 V-13, local collector *leg.* [KIZAS 0133744]. **PARATYPES:** 4 ♂♂, Tacheng (2510 m), Weixi County, Yunnan, China, 2023 V-17, local collector *leg*. [ZHJ]; 2♂♂, ♀, Tacheng (2510 m), Weixi County, Yunnan, China, 2024 V-12, local collector *leg*. [ZHJ].

***Ambulyx kuangtungensis* (Mell, 1922)**

♂, Mt. Baihuashan (1410 m), Beijing, China, 2016 VI-13, Kun Li *leg*. [ZHJ]; 2♂♂, ♀, Yintiaoling Nature Reserve (1270 m), Wuxi County, Chongqing, China, 2022 VII-22, Zhuo-Heng Jiang *leg*. [ZHJ]; 2♂♂, Donghe Township (560 m), Taitung County, Taiwan, China, 2018 VII-2, Wen-Yi Chou *leg*. [ZHJ]; 3♂♂, ♀, Mt. Tianmushan (920 m), Zhejiang, China, 2020 VII-13, Zhuo-Heng Jiang *leg*. [ZHJ]; ♂, Mt. Linshan (134 m), Hangzhou, Zhejiang, China, 2021 VII-24, Zhuo-Heng Jiang *leg*. [ZHJ]; 3♂♂, 2♀♀, Maolan Nature Reserve (896 m), Libo, Guizhou, China, 2017 IV-27, Zhuo-Heng Jiang *leg*. [ZHJ]; ♂, Hanjichong (810 m), Yuanjiang County, Yunnan, China, 2020 IX-27, Zhuo-Heng Jiang *leg*. [ZHJ]; 2♂♂, Mengla County (847 m), Yunnan, China, 2013 VIII-30, Zhuo-Heng Jiang *leg*. [ZHJ]; 2♂♂, Mt. Jianfengling (1012 m), Ledong County, Hainan, China, 2018 IV-17, Zhuo-Heng Jiang *leg*. [ZHJ].

***Ambulyx latifascia* Brechlin & Haxaire, 2014**

3♂♂, 2♀♀, Hutiaoxia (2310 m), Lijiang, Yunnan, China, 2020 VII-26, Hao-Lin Gan *leg*. [ZHJ]; ♂, Renhe Villiage (2210 m), Lijiang, Yunnan, China, 2017 VI-20, Hui-Hong Zhang *leg*. [ZHJ]; ♂, Yanbian County (2130 m), Panzhihua, Sichuan, China, 2019 VIII-7, Zhuo-Heng Jiang *leg*. [ZHJ]; ♂, Tacheng (2510 m), Weixi County, Yunnan, China, 2024 V-12, local collector *leg*. [ZHJ].

***Ambulyx liturata* Butler, 1875**

3♂♂, ♀, Youxi County (846 m), Sanming, Fujian, China, 2018 V-29, Zhuo-Heng Jiang *leg*. [ZHJ]; 2♂♂, ♀, Mt. Tianmushan (920 m), Zhejiang, China, 2020 VII-16, Zhuo-Heng Jiang *leg*. [ZHJ]; 2♂♂, Mengla County (847 m), Yunnan, China, 2013 VIII-30, Zhuo-Heng Jiang *leg*. [ZHJ]; 2♂♂, Mt. Jianfengling (1012 m), Ledong County, Hainan, China, 2018 IV-17, Zhuo-Heng Jiang *leg*. [ZHJ].

***Ambulyx maculifera* Walker, 1866**

♂, Xima Town (1500 m), Yingjiang County, Yunnan, China, 2018 VII-23, Zhuo-Heng Jiang *leg*. [ZHJ]; 2♂♂, Nabanhe Nature Reserve (1093 m), Xishuangbanna, Yunnan, China, 2022 VII-7, Chang-Qiu Liu *leg*. [ZHJ]; ♂, Motuo (1210 m), Xizang, China, 2022 VII-21, Shi-Wei Guo *leg*. [ZHJ]; ♂, Pu’er (1608 m), Yunnan, China, 2021 VII-18, Dan-Dan Xu *leg*. [ZHJ]; ♀, Xima Town (1500 m), Yingjiang County, Yunnan, China, 2022 VII, Wei-Zong Yang *leg*. [ZHJ].

***Ambulyx moorei* Moore, 1858**

3♂♂, Mt. Jianfengling (1012 m), Ledong County, Hainan, China, 2018 IV-17, Zhuo-Heng Jiang *leg*. [ZHJ]; 2♂♂, Mengla County (847 m), Yunnan, China, 2013 VIII-30, Zhuo-Heng Jiang *leg*. [ZHJ]; ♀, Xima Town (1500 m), Yingjiang County, Yunnan, China, 2018 VII-22, Zhuo-Heng Jiang *leg*. [ZHJ].

***Ambulyx ochracea* Butler, 1885**

3♂♂, ♀, Yintiaoling Nature Reserve (1270 m), Wuxi County, Chongqing, China, 2022 VII-22, Zhuo-Heng Jiang *leg*. [ZHJ]; 2♂♂, Yanbian County (1130 m), Panzhihua, Sichuan, China, 2019 VIII-3, Zhuo-Heng Jiang *leg*. [ZHJ]; 2♂♂, Youxi County (846 m), Sanming, Fujian, China, 2018 V-29, Zhuo-Heng Jiang *leg*. [ZHJ]; 2♂♂, ♀, Mt. Tianmushan (920 m), Zhejiang, China, 2020 VII-14, Zhuo-Heng Jiang *leg*. [ZHJ]; ♂, Mt. Linshan (134 m), Hangzhou, Zhejiang, China, 2021 VII-24, Zhuo-Heng Jiang *leg*. [ZHJ]; 3♂♂, Maolan Nature Reserve (896 m), Libo, Guizhou, China, 2017 IV-28, Zhuo-Heng Jiang *leg*. [ZHJ]; 2♂♂, Mengla County (793 m), Yunnan, China, 2013 VIII-29, Zhuo-Heng Jiang *leg*. [ZHJ]; 2♂, Mt. Jianfengling (1012 m), Ledong County, Hainan, China, 2018 IV-17, Zhuo-Heng Jiang *leg*. [ZHJ]; 2♂♂, ♀, Xima Town (1500 m), Yingjiang County, Yunnan, China, 2018 VII-22, Zhuo-Heng Jiang *leg*. [ZHJ].

***Ambulyx placida* Moore, 1888**

♂, Lixin Village (1370 m), Nyalam County, Xizang, China, 2020 VII-17, Si-Xun Ge *leg*. [ZHJ]; ♂, Motuo (1630 m), Xizang, China, 2023 VII-23, Yu-Fei Li *leg*. [ZHJ]; 3♂♂, Nyingchi City(1980 m), Xizang, China, 2024 VII-21, Xin Wang *leg*. [ZHJ].

***Ambulix schauffelbergeri* Bremer & Grey, 1853**

2♂♂, Mt. Fushoushan (1097 m), Yueyang, Hunan, China, 2016 IX-23, Zhuo-Heng Jiang *leg*. [ZHJ]; 2♂♂, Jiading new town (4 m), Jiading District, Shanghai, China, 2021 X, De-Rao Chou *leg*. [DRC]; ♂, Menghai County, Xishuangbanna, Yunnan, China, 2020 V-23, Chang-Qiu Liu *leg*. [ZHJ]; 2♂♂, ♀, Yaoluoping (990 m), Yuexi County, Anhui, China, 2023 IX-7, Zhuo-Heng Jiang *leg*. [ZHJ]; ♂, Mt. Laoshan (270 m), Nanjing, Jiangsu, China, 2023 VII-11, Zhuo-Heng Jiang *leg*. [ZHJ]; ♀, Zhenping County (1140 m), Shaanxi, China, 2023 VIII-13, Zhuo-Heng Jiang *leg*. [ZHJ].

***Ambulyx semiplacida semiplacida* Inoue, 1990**

2♂♂, Mt. Alishan (1450 m), Taiwan, China, 2017 V-6, Wen-Yi Chou *leg*. [ZHJ]; 6♂♂, 2♀♀, Nantou County, Taiwan, China, 2023 V-22, Local collector *leg*. [ZHJ]; 2♂♂, Nantou County, Taiwan, China, 2002 IV-18–19, D. Anstine *leg*. [SM]; ♀, Yilan County, Taiwan, China, 2011 X-6, Schnitzler *leg*. [SM]; ♀, Yilan County, Taiwan, China, 2011 X-6, Schnitzler *leg*. [SM].

***Ambulyx semiplacida interplacida* Brechlin, 2006**

♂, Shaoguan, Guangdong, China, 2017 V-2, Jin-Kun Zhang *leg*. [ZHJ]; 5♂♂, Mt. Leigongshan (1304 m), Suiyang County, Guizhou, China, 2021 V-3, Xin-Yi Zheng *leg*. [ZHJ]; ♀, Fengtongzhai (1802 m), Baoxing County, Sichuan, China, 2016 V-2, Zhi-Yuan Qi *leg*. [ZHJ].

***Ambulyx semiplacida montana* Cadiou & Kitching, 1990**

♂, Pingbian County (1980 m), Yunnan, China, 2024 VI-25, Shi-Wei Guo *leg*. [ZHJ]; ♂, ♂, Pingbian County (2012 m), Yunnan, China, 2024 V-21, Peng Wang *leg*. [ZHJ]; ♂, ♀, Pingbian County (2040 m), Yunnan, China, 2024 V-4, Yong-Kang You *leg*. [ZHJ].

***Ambulyx sericeipennis sericeipennis* Butler, 1875**

2♂♂, ♀, Yintiaoling Nature Reserve (1270 m), Wuxi County, Chongqing, China, 2022 VII-22, Zhuo-Heng Jiang *leg*. [ZHJ]; ♂, Yanbian County (1130 m), Panzhihua, Sichuan, China, 2019 VIII-3, Zhuo-Heng Jiang *leg*. [ZHJ]; 2♂♂, Youxi County (846 m), Sanming, Fujian, China, 2018 V-29, Zhuo-Heng Jiang *leg*. [ZHJ]; 2♂♂, ♀, Mt. Tianmushan (920 m), Zhejiang, China, 2020 VII-14, Zhuo-Heng Jiang *leg*. [ZHJ]; ♂, ♀, Mt. Alishan (1450 m), Taiwan, China, 2017 V-6, Wen-Yi Chou *leg*. [ZHJ]; 2♂♂, Mt. Fushoushan (1097 m), Yueyang, Hunan, China, 2016 IX-23, Zhuo-Heng Jiang *leg*. [ZHJ]; 3♂♂, ♀, Maolan Nature Reserve (896 m), Libo, Guizhou, China, 2017 IV-28, Zhuo-Heng Jiang *leg*. [ZHJ]; ♂, ♀, Lixin Village (1370 m), Nyalam County, Xizang, China, 2020 VII-17, Si-Xun Ge *leg*. [ZHJ].

***Ambulyx sericeipennis joiceyi* (Clark, 1923)**

3♂♂, 2♀♀, Sabah (1070 m), Borneo, Malaysia, 2023 IV-27, Zhuo-Heng Jiang *leg*. [ZHJ]; 2♂♂, Sabah (960 m), Borneo, Malaysia, 2018 V-16, Local collector *leg*. [ZHJ]; 4♂♂, ♀, Sumatra, Indonesia, 2023 VI, Local collector *leg*. [ZHJ].

***Ambulyx sericeipennis javanica* (Clark, 1930)**

3♂♂, Mt. Halimun (1020 m), Mt. Halimun, Java, Indonesia, 2022 V, Local collector *leg*.

***Ambulyx siamensis* Inoue, 1991**

3♂♂, Nabanhe Nature Reserve (1093 m), Xishuangbanna, Yunnan, China, 2022 VIII-29, Chang-Qiu Liu *leg*. [CQL, ZHJ]; ♂, Mt. Bulangshan (1450 m), Menghai County, Yunnan, China, 2021 IV-17, Chang-Qiu Liu *leg*. [CQL]; ♂, Xima Town (1500 m), Yingjiang County, Yunnan, China, 2022 VII, Wei-Zong Yang *leg*. [ZHJ].

***Ambulyx substrigilis* Westwood, 1847**

2♂♂, Nabanhe Nature Reserve (1093 m), Xishuangbanna, Yunnan, China, 2022 VIII-29, Chang-Qiu Liu *leg*. [CQL, ZHJ]; 2♂♂, Tongbiguan Township (1450 m), Yingjiang County, Yunnan, China, 2022 VIII-30, Chang-Qiu Liu *leg*. [CQL]; ♀, Xiajinchang Township, Malipo County, Yunnan, China, 2020 VII-26, Hao-Lin Gan *leg*. [ZHJ]; ♀, Bapo (1312 m), Dulongjiang, Yunnan, China, 2024 VI-6, Yi-Ting Lin *leg*. [ZBX]; ♂, Mt. Jianfengling (924 m), Ledong County, Hainan, China, 2018 IV-20, Zhuo-Heng Jiang leg. [ZHJ].

***Ambulyx tattina tattina* (Jordan, 1919)**

♂, Yiwu Town (1367 m), Mengla County, Xishuangbanna, Yunnan, China, 2013 VIII-31, Zhuo-Heng Jiang *leg*. [ZHJ]; ♂, ♀, Menghai County (1120 m), Xishuangbanna, Yunnan, China, 2022 IX-16, Dan-Dan Xu *leg*. [ZHJ].

***Ambulyx tobii* Inoue, 1976**

2♂♂, ♀, Yintiaoling Nature Reserve (1270 m), Wuxi County, Chongqing, China, 2022 VII-22, Zhuo-Heng Jiang *leg*. [ZHJ]; ♂, Huairou County (1210 m), Beijing, China, 2023 VII-20, Li Tao *leg*. [ZHJ]; ♂, Mt. Lingyanshan (2130 m), Dujiangyan City, Sichuan, China, 2015 VII-20, Yi Zhang *leg*. [ZHJ]; 2♂♂, Mt. Fushoushan (1097 m), Yueyang, Hunan, China, 2016 IX-23, Zhuo-Heng Jiang *leg*. [ZHJ]; ♂, ♀, Mt. Leigongshan (1304 m), Suiyang County, Guizhou, China, 2021 V-13, Xin-Yi Zheng *leg*. [ZHJ]; ♂, Yanjin County (1107 m), Yunnan, China, 2016 VII-17, Zhuo-Heng Jiang *leg*. [ZHJ]; 3♂♂, Xiajinchang Township, Malipo County, Yunnan, China, 2020 VII-26, Hao-Lin Gan *leg*. [ZHJ].

***Ambulyx zhejiangensis* Brechlin, 2009**

♂, Yintiaoling Nature Reserve (1370 m), Wuxi County, Chongqing, China, 2022 IV-11, Tian-Yu Ren *leg*. [ZHJ]; ♀, Tapa Shan, China, 2001 V, Local collector *leg*. [SM]; ♀, Yu-Shan-Wu, Anji County, Zhejiang, China, 2001 V, Local collector *leg*. [SM].

## References

[1] Westwood J.O. (1847). The Cabinet of Oriental Entomology.

[2] Kitching I. Sphingidae Taxonomic Inventory. https://sphingidae.myspecies.info/.

[3] Walker F. (1865). Supplement. List of the Specimens of Lepidopterous Insects in the Collection of the British Museum.

[4] Rothschild L.W. (1894). Notes on Sphingidae, with Descriptions of New Species. Novit. Zool..

[5] Mell R. (1922). Neue Südchinesische Lepidoptera. Dtsch. Entomol. Z..

[6] Brechlin R. (2014). Eine Neue Art Der Gattung Ambulyx Westwood, 1847 Aus Bhutan Und NO-Indien (Lepidoptera: Sphingidae). Entomo-Satsphingia.

[7] Butler A.G. (1875). Descriptions of New Species of Sphingidae. Proc. Zool. Soc. Lond..

[8] Walker F. (1866). List of the Specimens of Lepidopterous Insects in the Collection of the British Museum.

[9] Moore F. (1858). A Catalogue of the Lepidopterous Insects in the Museum of the Hon.

[10] Butler A.G. (1885). Descriptions of Moths New to Japan, Collected by Messrs. Lewis and Pryer. Cistula Entomol..

[11] Moore E. (1888). Descriptions of New Genera and Species of Lepidoptera Heterocera, Collected by Rev. JH Hocking, Chiefly in the Kangra District, NW Himalaya. Proceedings of the Zoological Society of London.

[12] Bremer O., Grey W., de Motschulsky V. (1852). Diagnoses de Lepidopteres Nouveaux Trouves Par MM. Tatarinoff et Gaschkewitsch Aux Environs de Peking. Études Entomologiques.

[13] Inoue H. (1990). Supplementary Notes on the Sphingidae of Taiwan, with Special Reference to Marumba Spectabilis-Complex. Tinea.

[14] Inoue H. (1976). Some New and Unrecorded Moths Belonging to the Families of Bombyces and Sphinges from Japan (Lepidoptera). Bull. Fac. Domest. Sci. Otsuma Women’s Univ..

[15] Inoue H. (1991). Records of the Sphingidae from Thailand, with Descriptions of Four New Species. Tinea.

[16] Brechlin R. (2009). Einige Anmerkungen Zur Sericeipennis-Gruppe Der Gattung Ambulyx Westwood, 1847 Mit Beschreibung Eines Neuen Taxons (Lepidoptera, Sphingidae). Entomo-Satsphingia.

[17] Okano M. (1964). New or Little Known Moths from Formosa (5). Tohoku Konchu Kenkyu.

[18] (1992). Inoue, H A New Subspecies of *Ambulyx placida* Moore from Nepal (Sphingidae). Lepidoptera Science.

[19] Brechlin R. (2006). Anmerkungen Zur Placida-Gruppe Der Gattung Ambulyx Westwood, 1847 Mit Beschreibung Einer Neuen Art (Lepidoptera: Sphingidae). Nachrichten Des Entomol. Ver. Apollo.

[20] Brechlin R., Haxaire J. (2014). Eine Neue Art Der Gattung Ambulyx Westwood, 1847 Aus Yunnan, China (Lepidoptera: Sphingidae). Entomo-Satsphingia.

[21] Katoh K., Standley D.M. (2013). MAFFT Multiple Sequence Alignment Software Version 7: Improvements in Performance and Usability. Mol. Biol. Evol..

[22] Capella-Gutiérrez S., Silla-Martínez J.M., Gabaldón T. (2009). trimAl: A Tool for Automated Alignment Trimming in Large-Scale Phylogenetic Analyses. Bioinformatics.

[23] Nguyen L.-T., Schmidt H.A., Von Haeseler A., Minh B.Q. (2015). IQ-TREE: A Fast and Effective Stochastic Algorithm for Estimating Maximum-Likelihood Phylogenies. Mol. Biol. Evol..

[24] Yang Z. (2007). PAML 4: Phylogenetic Analysis by Maximum Likelihood. Mol. Biol. Evol..

[25] Kawahara A.Y., Barber J.R. (2015). Tempo and Mode of Antibat Ultrasound Production and Sonar Jamming in the Diverse Hawkmoth Radiation. Proc. Natl. Acad. Sci. USA.

[26] Rambaut A., Drummond A.J., Xie D., Baele G., Suchard M.A. (2018). Posterior Summarization in Bayesian Phylogenetics Using Tracer. Syst. Biol..

[27] Kumar S., Stecher G., Tamura K. (2016). MEGA7: Molecular Evolutionary Genetics Analysis Version 7.0 for Bigger Datasets. Mol. Biol. Evol..

[28] Druce H. (1882). Descriptions of New Species of Aegeriidae and Sphingidae. Entomol. Mon. Mag..

[29] Clark B.P. (1936). Descriptions of Twenty-Four New Sphingidae and Notes Concerning Two Others. Proc. New Engl. Zool. Club.

[30] Mell R. (1937). Beiträge Zur Fauna Sinica. XIV. Ergänzungen Zur Sphingiden-, Brahmaeiden-und Eupterotidenfauna Chinas (Lep.). Berl. Entomol. Z..

[31] Gehlen B. (1942). Neue Sphingiden. Entomol. Z..

[32] Eitschberger U., Bergmann A., Hauenstein A. (2006). Drei Neue Arten Der Gattung Ambulyx Westwood, 1847 (Lepidoptera, Sphingidae). Atalanta.

[33] Butler A.G. (1881). Illustrations of Typical Specimens of Lepidoptera Heterocera in the Collection of the British Museum.

[34] Felder C., Felder R. (1867). Reise Der Österreichischen Fregatte Novara Um Die Erde. Den Jahren 1857, 1858, 1859 Unter Den Befehlen Des Commodore B. von Wüllerstorf-Urbair. Lepidoptera; Rhopalocera.

[35] Moore F. (1882). The Lepidoptera of Ceylon.

[36] Huwe A. (1895). Verzeichniss Der von Hans Fruhstorfer Während Seines Aufenthalts Auf Java in Den Jahren 1891 Bis 1893 Erbeuteten Sphingiden. Berl. Entomol. Z..

[37] Clark B.P. (1922). Twenty-Five New Sphingidae. Proc. N. Engl. Zool. Club.

[38] Kishida Y. (2018). Notes on Japanese *Ambulyx sericeipennis*, with Description of a New Subspecies from Hokkaido. Jpn. Heterocerists’ J..

[39] Joicey J.J., Kaye W.J. (1917). New Species and Forms of Sphingidae. Ann. Mag. Nat. Hist..

[40] Mell R. (1922). Beiträge Zur Fauna Sinica. Biologie Und Systematik Der Südchinesischen Sphingiden.

[41] Clark B.P. (1937). Twelve New Sphingidae and Notes on Seven Others. Proc. N.Engl. Zool. Club.

[42] Kobayashi H., Wang M., Yano T. (2006). A New Species of the Genus Ambulyx from Guangdong, China and Remarks upon *Ambulyx sericeipennis* Butler (Lepidoptera, Sphingidae). Tinea.

[43] Jordan K. (1929). On Some Oriental Sphingidae. Novit. Zool. Tring.

[44] Okano M. (1958). New or Little Known Moths from Formosa (1). Report of the Gakugei Faculty of Iwate University.

[45] Meng X.W. (1989). A New Species of Oxyambulyx from China (Lepidoptera: Sphingidae). Entomotaxonomia.

[46] Clark B.P. (1923). Thirty-Three New Sphingidae. Proc. N. Engl. Zool. Club.

[47] Clark B.P. (1930). Sundry Notes on Sphingidae and Descriptions of Seven New Forms. Proc. N. Engl. Zool. Club.

[48] Clark B.P. (1924). Twelve New Sphingidae. Proc. N. Engl. Zool. Club.

[49] Jiang Z.H., Ge S.X., Xu Z.B. (2020). New Records of Four Hawkmoths Species (Lepidoptera, Sphingidae) from China. Sichuan J. Zool..

[50] Gehlen B. (1940). Sieben Neue Sphingiden. Entomol. Z..

[51] Jiang Z.-H., Huang C.-L. (2023). Hawkmoths of China.

[52] Bell T.R.D., Scott F.B. (1937). The Fauna of British India, Including Ceylon and Burma. Moths.

[53] Nakamura M. (1977). Supplement to the Pupae of Japanese Sphingidae (Lepidoptera). New Entomol..

[54] Yano T. (1994). The Larva of *Ambulyx japonica* Rothschild (Sphingidae). Jpn. Heterocerists’ J..

[55] Zolotuhin V., Sinev S.Y. (2019). V Sphingidae. Catalogue of the Lepidoptera of Russia.

[56] Koshkin E.S. (2021). New and Interesting Records of Lepidoptera from the Southern Amur Region, Russia (Insecta: Lepidoptera). SHILAP Rev. De Lepidopterol..

[57] Eitschberger U., Ihle T. (2010). Raupen von Schwärmern Aus Laos Und Thailand–1. Beitrag (Lepidoptera, Sphingidae). Neue Entomol. Nachrichten.

[58] Haxaire J., Melichar T. (2023). Contribution to the Knowledge of the Sphingidae of Arunachal Pradesh, with Taxonomic Notes on the Genera Eupanacra Cadiou & Holloway, 1989, Acosmeryx Boisduval, 1875, Ambulyx Westwood, 1847 and Marumba Moore, 1882 (Lepidoptera, Sphingidae). Eur. Entomol..

[59] Jiang Z.H., Xiong Z.C., Gan H.L. (2022). Description of Three Newly Recorded Species of Hawkmoths in China (Lepidoptera: Sphingidae). J. Zhejiang For. Sci. Technol..

[60] Pan X., Han H. (2018). A New Species and a Newly Recorded Species in the Genus Macroglossum Scopoli (Lepidoptera: Sphingidae) from China. Entomotaxonomia.

[61] Jiang Z.H., Lou W.R., Zhang H.H. (2021). New Records of Hawkmoths from China (Lepidoptera: Sphingidae): Two Species. Sichuan J. Zool..

[62] Jiang Z.H., Zhang H.H. (2024). New Species Records of Sphingidae from China. J. Zhejiang For. Sci. Technol..

[63] Jiang Z.H., Yan M., Wang J.X., Hu S.J. (2024). Review of the Genus Pseudodolbina Rothschild, 1894, with the Description of a New Species from Yunnan, China (Lepidoptera: Sphingidae). Zootaxa.

[64] Xu Z.-B., Melichar T., He J.-B., Zhang C., Zhang X.-Y., Feng D.U., Hu S.-J. (2022). A New Species of Rhodambulyx Mell, 1939 (Lepidoptera: Sphingidae) from Southwest Chongqing, China. Zootaxa.

[65] Jordan K. (1919). On Some Oriental Sphingidae. Novit. Zool..

[66] Clark B.P. (1938). Eight New Sphingidae and Notes on Two Others. Proc. N. Engl. Zool. Club.

[67] Cadiou J.M., Kitching I.J. (1990). New Sphingidae from Thailand (Lepidoptera). Lambillionea.

[68] Quynh Le T., Vu L. (2024). Checklist of Hawkmoths (Lepidoptera: Bombycoidea: Sphingidae) in the Central Highlands of Vietnam. J. Threat. Taxa.

