# Correction: Jiang et al. A Review of the Genus *Ambulyx* Westwood, 1847 (Lepidoptera: Sphingidae) from China Based on Morphological and Phylogenetic Analyses, with the Description of a New Species. *Insects* 2025, *16*, 223

**DOI:** 10.3390/insects16090920

**Published:** 2025-09-02

**Authors:** Zhuo-Heng Jiang, Ian J. Kitching, Xiao-Dong Xu, Zhen-Bang Xu, Ming Yan, Wen-Bo Yu, Chang-Qiu Liu, Shao-Ji Hu

**Affiliations:** 1 Yunnan Key Laboratory of International Rivers and Transboundary Eco-Security, Yunnan University, Kunming 650500, China; jzhsphingidae@163.com (Z.-H.J.); zhenbangxumm@gmail.com (Z.-B.X.); 2 Institute of International Rivers and Eco-Security, Yunnan University, Kunming 650500, China; 3 School of Life Science, Westlake University, Hangzhou 310023, China; xuxiaodong@westlake.edu.cn; 4 Natural History Museum, Cromwell Road, London SW7 5BD, UK; i.kitching@nhm.ac.uk; 5 Anhui Provincial Key Laboratory of the Conservation and Exploitation of Biological Resources, College of Life Sciences, Anhui Normal University, Wuhu 241000, China; 15050598630@163.com; 6 College of Landscape Architecture, Nanjing Forestry University, Nanjing 210037, China; yuwenbo@njfu.edu.cn; 7 Guangxi Institute of Botany, Chinses Academy of Sciences, Guilin 541006, China; changqiuliu@whu.edu.cn

## Text Correction

In the original publication [1], the ZooBank unique identification code (LSID—Life Science Identifier) is missing. The following information should be added. The authors state that the scientific conclusions are unaffected. This correction was approved by the Academic Editor. The original publication has been edited to include the LSIDs, and the journal published a correction notice on the website.

## 3. Results

### *3.2.5.* Ambulyx wukong *Jiang & Kitching **sp. nov.** [悟空鹰翅天蛾]*

Article registration: urn:lsid:zoobank.org:pub:C35EA7C0-4F7A-4572-A204-BDBDB36F8E2E

Species registration: urn:lsid:zoobank.org:act:3B8A06A9-D158-4B8F-BD47-44F4F8F02810

**Type data: HOLOTYPE:** ♂, Tacheng (2350 m), Weixi County, Yunnan, China, 2023 V-13, local collector leg. [KIZAS 0133744]. **PARATYPES:** 4♂♂, Tacheng (2510 m), Weixi County, Yunnan, China, 2023 V-17, local collector *leg*. [ZHJ]; 2♂♂, ♀, Tacheng (2510 m), Weixi County, Yunnan, China, 2024 V-12, local collector *leg*. [ZHJ].
