# Supplementary material for: A Review of the Genus Ambulyx Westwood, 1847 (Lepidoptera: Sphingidae) from China Based on Morphological and Phylogenetic Analyses, with the Description of a New Species
Source: Insects. 2025 Feb 18;16(2):223. doi: 10.3390/insects16020223 (PMC11856168; doi:10.3390/insects16020223)
Supplement: Supplementary file 1 [file insects-16-00223-s001.zip › insects-3393664-supplementary.pdf]

**Table S1.** Sampling information and BOLD SampleID/GenBank accession numbers of *Ambulyx* and outgroups samples used in this study. The taxon names follow current taxonomy

| Taxon (Sample Code) | Locality | Collecting Date | GenBank No. | Bold ID |
|---|---|---|---|---|
| *Ambulyx aglaia* | Tamil Nadu, India | 1997-IV-08 | — | BC-RBP-1941 |
| *Ambulyx aglaia* | Tamil Nadu, India | 1997-IV-08 | — | BC-RBP-1942 |
| *Ambulyx aglaia* | Tamil Nadu, India | 1997-IV-08 | GU704276 | — |
| *Ambulyx amboynensis* | Ambon, Indonesia | 1992-VII-20 | — | BC-RBP-2130 |
| *Ambulyx amboynensis* | Buru, Indonesia | 1998-X-01 | — | BC-RBP-2133 |
| *Ambulyx andangi* | Tanimbar, Indonesia | 2005-XI-01 | — | Bc-Mel_0104b |
| *Ambulyx andangi* | Tanimbar-isls., Indonesia | 1996-V-20 | — | BC-RBP-1776 |
| *Ambulyx auripennis* | Deniyaya, Sri Lanka | 1985-XII-01 | — | BC-Hax0156 |
| *Ambulyx auripennis* | Deniyaya, Sri Lanka | 1985-XII-01 | JN677693 | — |
| *Ambulyx bakeri* | Leyte island, Philippines | 1985-XII-01 | — | BC-Hax0077 |
| *Ambulyx bakeri* | Eastern Visayas, Philippines | 1992-X-01 | — | BC-Hax0079 |
| *Ambulyx belli* | India | No date | — | BC-Mel_0108b |
| *Ambulyx belli* | Maharashtra, India | 2005-IX-26 | — | MA05-08-27-26 |
| *Ambulyx bima bima* | Nusa Tenggara Barat, Indonesia | 2000-V-01 | — | BC-Hax0128 |
| *Ambulyx bima bima* | Sumbawa, Indonesia | 1998-I-01 | — | BC-RBP-2154 |
| *Ambulyx bima timoriana* | Timor W, Indonesia | 2007-IV-10 | — | BC-RBP-1774 |
| *Ambulyx canescens* | Yingjiang, Yunnan, China | 2018-VII-26 | PO722229 | — |
| *Ambulyx canescens* | Lamphun, Thailand | 2001-VIII-12 | — | BC-Hax0509 |
| *Ambulyx carycina* | West New Britain, Papua New Guinea | 2003-IV-04 | — | BC-RBP-1783 |
| *Ambulyx carycina* | West New Britain, Papua New Guinea | 2003-IV-04 | — | BC-RBP-1784 |
| *Ambulyx celebensis celebensis* | Sulawesi, Indonesia | 1995-X-16 | — | BC-Hax0086 |
| *Ambulyx celebensis celebensis* | Mimaropa, Philippines | 1998-XI-26 | — | BC-RBP-2061 |
| *Ambulyx charlesi* | Ceram, Indonesia | 1999-X-01 | JN677700 | — |
| *Ambulyx clavata* | Borneo, Malaysia | 1991-VII-13 | — | BC-Hax0088 |
| *Ambulyx clavata* | Borneo, Malaysia | 1991-VII-13 | JN677701 | |
| *Ambulyx cyclasticta* | Saravan, Laos | 1997-VII-25 | — | BC-Hax0151 |
| *Ambulyx cyclasticta* | Lamphun, Thailand | 2001-VIII-12 | — | BC-Hax0499 |
| *Ambulyx dohertyi dohertyi* | Papua Barat, Indonesia | 1998-II-26 | — | BC-RBP-2138 |
| *Ambulyx dohertyi dohertyi* | Morobe, Papua New Guinea | 1999-IV-08 | — | BC-RBP-2137 |
| *Ambulyx dohertyi isabeliana* | Santa Isabel, Solomon Islands | 1998-I-28 | — | BC-RBP-2146 |
| *Ambulyx dohertyi* | Vietnam | 2014-IX-09 | MK804151 | — |
| *Ambulyx dohertyi novobritannica* | West New Britain, Papua New Guinea | 2003-III-19 | — | BC-RBP-2151 |
| *Ambulyx dohertyi novobritannica* | West New Britain, Papua New Guinea | 2003-III-19 | — | BC-RBP-2152 |
| *Ambulyx dohertyi novoirlandensis* | New Ireland, Papua New Guinea | 2000-III-11 | — | BC-RBP-2149 |
| *Ambulyx dohertyi novoirlandensis* | New Ireland, Papua New Guinea | 2000-III-11 | — | BC-RBP-2150 |
| *Ambulyx dohertyi queenslandi* | Queensland, Australia | 2005-X-16 | KJ168784 | — |
| *Ambulyx dohertyi queenslandi* | Ferguson Island, Papua New Guinea | 2005-II-11 | — | BC-RBP-2142 |
| *Ambulyx dohertyi salomonis* | Mt. Austin, Guadalcanal, Solomon Islands | 2007-III-18 | — | BC-RBP-2145 |

| | | | | |
|---|---|---|---|---|
| *Ambulyx dohertyi salomonis* | San Cristobal, Solomon Islands | 2008-III-08 | — | BC-RBP-2147 |
| *Ambulyx flava flava* | Leyte island, Philippines | 2003-XI-19 | — | BC-Hax0179 |
| *Ambulyx flavocelebensis* | Sulawesi Selatan, Indonesia | 1996-XII-01 | — | BC-RBP-1882 |
| *Ambulyx flavocelebensis* | Sulawesi Selatan, Indonesia | 1997-X-01 | — | BC-RBP-1883 |
| *Ambulyx immaculata* | Leyte island, Philippines | 2003-IX-26 | — | BC-Hax0033 |
| *Ambulyx immaculata* | Panay islands, Philippines | 1997-XII-25 | — | BC-Hax0036 |
| *Ambulyx inouei* | Central Sulawesi, Indonesia | 2000-XII-08 | — | BC-Hax0134 |
| *Ambulyx inouei* | Southern Sulawesi, Indonesia | 2000-VII-01 | — | BC-Hax0136 |
| *Ambulyx japonica angustifasciata* | Nantou, Taiwan, China | 2005-V-02 | — | BC-EMEM0622 |
| *Ambulyx japonica angustifasciata* | Taidong, Taiwan, China | 2004-VIII-01 | — | AYK-04-0287 |
| *Ambulyx japonica japonica* | Aichi, Japan | 2001-VII-07 | — | BC-EMEM0624 |
| *Ambulyx japonica japonica* | Yamanashi, Japan | 2002-VI-01 | — | BC-EMEM0623 |
| *Ambulyx japonica koreana* | Shaanxi, China | 2000-VII-01 | — | BC-EMEM0628 |
| *Ambulyx japonica koreana* | Sichuan, China | 2005-V-14 | — | BC-EMEM0626 |
| *Ambulyx johnsoni* | Leyte island, Philippines | 2003-XI-19 | — | BC-Hax0113 |
| *Ambulyx johnsoni* | Leyte island, Philippines | 2003-IX-26 | — | BC-Hax0115 |
| *Ambulyx jordani* | Irian Jaya, Papua New Guinea | 2003-IV-01 | — | BC-EMEM0600 |
| *Ambulyx jordani* | Irian Jaya, Papua New Guinea | 2003-IV-01 | — | BC-EMEM0601 |
| *Ambulyx kuangtungensis* | Shaanxi, China | 1998-XI-01 | — | BC-EMEM0866 |
| *Ambulyx kuangtungensis* | Sichuan, China | 1999-VII-01 | — | BC-EMEM0862 |
| *Ambulyx labuanensis* | Nusa Tenggara Timur, Indonesia | 2002-II-14 | — | BC-RBP-2099 |
| *Ambulyx labuanensis* | Nusa Tenggara Timur, Indonesia | 2002-II-03 | — | BC-RBP-2100 |
| *Ambulyx lahora* | Azk, Pakistan | 2010-VII-15 | — | MOT-00968 |
| *Ambulyx latifascia* | Yunnan, China | 1995-VIII-01 | — | BC-Hax0189 |
| *Ambulyx latifascia* | Yunnan, China | 1998-IV-27 | — | BC-RBP-2014 |
| *Ambulyx lestradei* | Sri Lanka | 1985-XII-01 | — | BC-Hax0194 |
| *Ambulyx liturata* | Jiangxi-Fujian border, China | 2002-V-01 | — | BC-Hax0106 |
| *Ambulyx liturata* | Hubei, China | 1998-VIII-01 | — | BC-EMEM0706 |
| *Ambulyx maculifera* | Motuo, Xizang, China | 2022-VII-14 | PO722233 | — |
| *Ambulyx maculifera* | Jinghong, Yunnan, China | 2022-VII-11 | PO722232 | — |
| *Ambulyx marissa* | Mt.Seribu, OBI isl., Indonesia | 2008-V-01 | — | BC-Mel_1009 |
| *Ambulyx marissa* | Mt.Seribu, OBI isl., Indonesia | 2008-V-01 | — | BC-Mel_1010 |
| *Ambulyx matti* | Goa, India | 2005-IX-25 | — | MA05-08-23-57 |
| *Ambulyx matti* | Karnataka, India | 2008-VII-01 | — | BC-Mel_1011 |
| *Ambulyx meeki meeki* | Mt. Austin, Guadalcanal, Solomon Islands | 2007-III-18 | — | BC-RBP-1790 |
| *Ambulyx meeki meeki* | Guadalcanal, Solomon Islands | 1998-II-03 | — | BC-RBP-1791 |
| *Ambulyx moorei* | Menglun, Yunnan, China | 2023-VIII-22 | PO723097 | — |
| *Ambulyx moorei* | Tonkin, Mt. Fan Si Pan, Vietnam | 1994-X-01 | — | BC-RBP-2159 |
| *Ambulyx obliterata* | Borneo, Sabah, Malaysia | 2000-V-01 | — | BC-Hax0057 |
| *Ambulyx obliterata* | Borneo, Sabah, Malaysia | 1993-V-01 | — | BC-Hax0058 |
| *Ambulyx obliterata* | Borneo, Malaysia | 2000-V-01 | JN677718 | |
| *Ambulyx ochracea* | Shaanxi, China | 1998-XI-01 | — | BC-EMEM0866 |
| *Ambulyx ochracea* | Sichuan, China | 1996-VII-01 | — | BC-Hax0125 |

| | | | | |
|---|---|---|---|---|
| *Ambulyx phalaris* | Timika, Indonesia | 2000-VII-01 | — | BC-Hax0161 |
| *Ambulyx phalaris* | Lakekamu Basin, Gulf, Papua New Guinea | 2000-III-10 | — | USNM ENT 00196045 |
| *Ambulyx placida* | Jigme Dorji Nat.Park, Bhutan | 2008-VI-04 | — | BC-RBP-1754 |
| *Ambulyx placida* | Tibet, China | 1996-VI-08 | — | BC-RBP-1756 |
| *Ambulyx pryeri pryeri* | Cameron highlands, Malaysia | 1990-I-01 | — | BC-Hax0163 |
| *Ambulyx pryeri pryeri* | Kanchanaburi, Thailand | 1998-IX-25 | — | BC-Hax0162 |
| *Ambulyx pryeri tenggarensis* | Nusa Tenggara Timur, Indonesia | 1996-IV-22 | — | BC-RBP-1765 |
| *Ambulyx pseudoclavata* | Chiang Mai, Thailand | 1999-III-20 | — | BC-Hax0087 |
| *Ambulyx pseudoclavata* | Ban na Hin, Laos | 2008-V-01 | — | BC-Mel0482 |
| *Ambulyx rawlinsi* | Ceram, Indonesia | 1999-X-01 | — | BC-Hax0167 |
| *Ambulyx schauffelbergeri* | Sichuan, China | 2001-VI-01 | — | BC-EMEM0695 |
| *Ambulyx schauffelbergeri* | Jiangxi, China | 2003-VII-01 | — | BC-RBP-2032 |
| *Ambulyx schauffelbergeri* | - | - | OP219770 | — |
| *Ambulyx semifervens* | Maluku, Indonesia | 1998-I-06 | — | BC-EMEM0613 |
| *Ambulyx semiplacida bhutana* | Black Mt. Nat.Park N, Bhutan | 2008-VI-06 | — | BC-RBP-1749 |
| *Ambulyx semiplacida bhutana* | Black Mt. Nat.Park N, Bhutan | 2008-VI-06 | — | BC-RBP-1750 |
| *Ambulyx semiplacida interplacida* | Jiangxi, China | 2003-VII-01 | — | BC-RBP-1757 |
| *Ambulyx semiplacida interplacida* | Sichuan, China | 2005-V-14 | — | BC-EMEM0872 |
| *Ambulyx semiplacida interplacida* | Hunan, China | No date | — | BC-Hax4475 |
| *Ambulyx semiplacida montana* | Doi (Mt.) Phahompok, Chiang Mai, Thailand | 1998-IV-02 | — | BC-RBP-1742 |
| *Ambulyx semiplacida montana* | Tonkin, Mt. Fan Si Pan, Vietnam | 1995-IV-08 | — | BC-RBP-1745 |
| *Ambulyx semiplacida montana* | Pingbian, Yunnan, China | 2024-V-04 | PQ656313 | — |
| *Ambulyx semiplacida semiplacida* | Nantou, Taiwan, China | 1997-V-29 | — | BC-RBP-1760 |
| *Ambulyx semiplacida semiplacida* | Nantou, Taiwan, China | 2002-IV-19 | — | BC-Mel0034 |
| *Ambulyx sericeipennis javanica* | Jawa Barat, Indonesia | 2000-IV-01 | — | BC-RBP-1737 |
| *Ambulyx sericeipennis joiceyi* | Borneo, Malaysia | 1991-VIII-09 | JN677708 | — |
| *Ambulyx sericeipennis luzoni* | Central Luzon, Philippines | 1996-VII-21 | — | BC-RBP-1740 |
| *Ambulyx sericeipennis palawanica* | Mt. Matalingahan, Palawan, Mimaropa, Philippines | 2000-VIII-02 | — | BC-RBP-1730 |
| *Ambulyx sericeipennis sericeipennis* | Hunan, China | 1998-VIII-01 | — | BC-EMEM0719 |
| *Ambulyx sericeipennis sericeipennis* | Guangxi, China | 2001-VI-01 | — | BC-EMEM0869 |
| *Ambulyx siamensis* | Jinghong, Yunnan, China | 2022-VI-11 | PO723101 | — |
| *Ambulyx siamensis* | Magway, Myanmar | 2005-VI-29 | — | BC-EMEM0658 |
| *Ambulyx siamensis* | Trat, Thailand | 2005-X-07 | — | BC-EMEM0657 |
| *Ambulyx staudingeri* | Central Luzon, Philippines | 2008-II-15 | — | BC-RBP-2044 |
| *Ambulyx staudingeri* | Panay islands, Philippines | 1997-III-03 | — | BC-Hax0157 |
| *Ambulyx substrigilis* | Bangladesh | 1997-V-01 | — | BC-EMEM0587 |
| *Ambulyx substrigilis* | Jinghong, Yunnan, China | 2022-VI-11 | PO723100 | — |
| *Ambulyx substrigilis* | Yingjiang, Yunnan, China | 2023-VII | PO723362 | — |
| *Ambulyx tattina tattina* | Mengla Count, Yunnan, China | 2023-XI-17 | PQ656314 | — |
| *Ambulyx tattina uichancoi* | Central Luzon, Philippines | 2008-IV-06 | — | BC-RBP-2067 |
| *Ambulyx tenimberi* | Sulawesi, Indonesia | 1998-II-01 | — | BC-Hax0168 |
| *Ambulyx tenimberi* | Southern Sulawesi, Indonesia | 2000-XII-03 | — | BC-Hax0170 |

| | | | | |
|---|---|---|---|---|
| *Ambulyx tobii* | Guizhou, China | 2002-VII-01 | — | BC-EMEM0716 |
| *Ambulyx tobii* | Kunming, Yunnan, China | 2023-VII-18 | PO723102 | - |
| *Ambulyx tondanoi* | Sulawesi, Indonesia | 2000-XII-04 | — | BC-Hax0141 |
| *Ambulyx tondanoi* | Sulawesi Selatan, Indonesia | 1995-I-30 | — | BC-RBP-1778 |
| *Ambulyx wildei* | Mt. Sibera, Maluku, Indonesia | 1996-II-02 | — | BC-RBP-1805 |
| *Ambulyx wildei* | Mt. Sibera, Maluku, Indonesia | 1996-II-02 | — | BC-RBP-1807 |
| *Ambulyx wildei* | West New Britain, Papua New Guinea | 2003-III-26 | GU704167 | — |
| *Ambulyx wildei* | Maluku, Indonesia | 2003-IX-08 | KJ168447 | — |
| *Ambulyx wilemani* | Mt. Halcon, Mindoro island, Philippines | 1997-VI-01 | — | BC-Mel_0106b |
| *Ambulyx wilemani* | Panay islands, Philippines | 1997-XII-31 | — | BC-Hax0107 |
| *Ambulyx wukong* **sp. nov.** AWX01 | Weixi County, Yunnan, China | 2023-V-12 | PQ658368 | — |
| *Ambulyx wukong* **sp. nov.** AWX02 | Weixi County, Yunnan, China | 2023-V-12 | PQ658369 | — |
| *Ambulyx zacharovi* | Borneo, Sabah, Malaysia | 2006-IV-02 | — | BC-Ivsh00525 |
| *Ambulyx zacharovi* | Borneo, Sabah, Malaysia | 2006-IV-02 | — | BC-Ivsh02079 |
| *Ambulyx zhejiangensis* | Wuxi, Chongqing, China | 2022-IV-22 | PO729887 | — |
| *Ambulyx zhejiangensis* | Anji county, Zhejiang, China | 1999-VI-10 | — | BC-RBP-1787 |
| *Amplypterus panopuspanopus* | Sabah, Malaysia | 1992-V-01 | JN677744 | — |
| *Anambulyx elwesi* | Punjab, Pakistan | 2013-VII-16 | KX862714 | — |
| *Anambulyx elwesi* | Punjab, Pakistan | 2013-VII-16 | KX863252 | — |
| *Barbourion lemaii* | Chiang Mai, Thailand | 2000-XI-25 | — | BC-Hax2471 |
